# Supplementary material for: The successful reintroduction of African wild dogs (Lycaon pictus) to Gorongosa National Park, Mozambique
Source: PLoS One. 2021 Apr 22;16(4):e0249860. doi: 10.1371/journal.pone.0249860 (PMC8062010; doi:10.1371/journal.pone.0249860)
Supplement: S1 Table — The number of fixes per wild dog and lion group used in the spatial analyses. The collar coverage dates represent the full time period from group release from enclosure/pack formation except for Cheza pack that formed in March 2019 (Fig 2) but was only GPS collared in August 2019. For the lion data, we only included data from the date of the release of the first pack of wild dogs on 15 June 2018. (DOCX) [file pone.0249860.s001.docx]

**S1 Table.** **Wild dog and lion collars**. The number of fixes per wild dog and lion group used in the spatial analyses. The collar coverage dates represent the full time period from group release from enclosure/pack formation except for Cheza pack that formed in March 2019 (Fig 2) but was only GPS collared in August 2019. For the lion data, we only included data from the date of the release of the first pack of wild dogs on 15 June 2018.

| **Group** | | **ID** | **Collar dates** | **Full dataset** | **Truncated dataset** |
| --- | --- | --- | --- | --- | --- |
| *Wild dogs* | | | | | |
|  | Gorongosa^1^ | Beira | 15 June 18 – 3 May 19 | 2,320 | 940 |
|  |  | Metuchira | 2 June 2019 - 29 Apr 2020 | 733 | 320 |
|  |  | Nhamagaia | 30 Apr 2020 – 30 Sep 2020 | 263 | 102 |
|  | Pwadzi^1^ | Mutiabamba | 11 Dec 2019 – 29 Sep 2020 | 708 | 218 |
|  | Cheza^1^ | Bebedo | 12 Aug 2019 – 19 Sep 2020 | 1,063 | 443 |
|  |  | Nhagutua | 20 Sep 2020 – 30 Sep 2020 | 52 | 28 |
|  | Mopane^1^ | Sapirandzi | 24 Nov 2019 – 29 Sep 2020 | 861 | 314 |
|  | Mucodza^2^ | Ndhapiona | 1 June 2019 – 9 Sep 2020 | 838 | 308 |
|  | Xivulo^2^ | Nhambita | 3 Dec 2019 – 29 Sep 2020 | 1,106 | 375 |
| *Lions* | | | | | |
|  | Sungwe^1^ | Flavia | 15 June 18 – 22 Nov 18 | 911 | 468 |
|  |  |  | 17 Dec 18 – 3 Mar 20 | 2,919 | 482 |
|  |  |  | 9 Mar 20 – 30 Sep 20 | 1,091 | 182 |
|  | Tenda^1^ | | 20 June 18 – 30 Nov 19 | 2,653 | 441 |
|  | Zangwe^1^ | | 6 Nov 18 – 4 Sep 19 | 657 | 113 |
|  | Nhacasena^1^ | | 25 Jul 19 – 27 Aug 20 | 1,200 | 202 |
|  | Mopane^1^ | | 12 Oct 19 – 30 Sep 20 | 1,358 | 218 |
|  | Manondo^3^ | | 15 June 18 – 25 Jul 18 | 223 | 118 |
|  |  |  | 24 Nov 19 – 1 Apr 20 | 673 | 110 |
|  | Senadore^3^ | | 15 Jul 18 – 25 Sep 19 | 2,108 | 1,223 |
|  |  |  | 26 May 20 – 30 Sep 20 | 684 | 123 |
|  | Sangrassa^3^ | | 15 Dec 19 – 30 Sep 20 | 1,398 | 239 |
|  | Neredzi^3^ | | 27 Feb 20 – 30 Sep 20 | 1,049 | 172 |
|  | 2 males^2^ | | 9 June 19 – 25 Jan 20 | 1,027 | 167 |
|  | Lua Cheia^2^ | | 22 June 19 – 2 Mar 20 | 1,225 | 203 |
|  | 3 males^2^ | | 25 Sep 19 – 30 Sep 20 | 1,214 | 205 |
|  | Musicadzi^2^ | | 17 Sep 19 – 30 Sep 20 | 1,846 | 297 |
|  | Sadunjira^2^ | | 25 Sep 19 – 30 Sep 20 | 1,157 | 192 |

^1^pack (wild dog) or pride (lion), ^2^dispersal group, ^3^coalition
