# Supplementary material for: The successful reintroduction of African wild dogs (Lycaon pictus) to Gorongosa National Park, Mozambique
Source: PLoS One. 2021 Apr 22;16(4):e0249860. doi: 10.1371/journal.pone.0249860 (PMC8062010; doi:10.1371/journal.pone.0249860)
Supplement: S2 Table — Pregnancies and confirmed litters for eight female wild dogs in Gorongosa National Park between June 2018 and September 2020. (DOCX) [file pone.0249860.s002.docx]

**S2 Table. Birth details.** Pregnancies and confirmed litters for eight female wild dogs in Gorongosa National Park between June 2018 and September 2020.

| **Pack** | **Breeding female(s) name – dominance status** | **Years** | **Details** |
| --- | --- | --- | --- |
| Gorongosa | Beira – alpha^1^ | 2018 | Alpha became pregnant, went underground to den and confirmed lactating during July. No pups emerged from den. Pack abandoned den in August. Large African rock python observed leaving the den in August shortly after pack left the den. |
| Gorongosa | Beira – alpha  Nhamagaia – beta | 2019 | Alpha and one subordinate female became pregnant. Both females’ litters emerged from dens with females raising them collectively. |
| Cheza | Nhagutua – alpha^2^ | 2019, 2020 | Alpha became pregnant, denned and litters emerged from dens. |
| Gorongosa | Beira – alpha  Nhamagaia – beta^1^  Ndarassica – gamma | 2020 | Alpha and two subordinate females became pregnant and denned. We only observed two females’ litters to emerge from the dens (i.e. beta female’s litter never confirmed despite her obvious signs of pregnancy and denning briefly (2.5km away) as the gamma female). |
| Pwadzi | Matenga – presumed alpha  Mutiabamba – alpha  Munhangana – beta | 2020 | Presumed alpha suspected denning, but found dead in her den. New alpha and one beta female confirmed pregnant and denned. However, the beta left pack during denning (she began denning before the alpha) and the beta is raising the pups alone (S3 Table). Both the alpha and beta females’ litters emerged from their respective dens. |
| Mopane | Sapirandzi – alpha^2^ | 2020 | Alpha became pregnant, denned and litter emerged from den. |

^1^when no pups were confirmed, ^2^only one female in the pack
