# Supplementary material for: The successful reintroduction of African wild dogs (Lycaon pictus) to Gorongosa National Park, Mozambique
Source: PLoS One. 2021 Apr 22;16(4):e0249860. doi: 10.1371/journal.pone.0249860 (PMC8062010; doi:10.1371/journal.pone.0249860)
Supplement: S3 Table — Details of events that resulted in individual founder wild dogs changing their group association in Gorongosa National Park from the time of the first reintroduction in June 2018 until September 2020. AM = adult male, YF = yearling female and AF = adult female. (DOCX) [file pone.0249860.s003.docx]

**S3 Table. Group association dynamics.** Details of events that resulted in individual founder wild dogs changing their group association in Gorongosa National Park from the time of the first reintroduction in June 2018 until September 2020. AM = adult male, YF = yearling female and AF = adult female.

| **Event type** | **Group size, sex and age** | **Event date** | **Comments** |
| --- | --- | --- | --- |
| Split | 3AM, 1YF | 26 March 2019 | Split from Gorongosa pack to form Cheza pack |
| Dispersal | 1AF | 1 July 2019 | Dispersed from Gorongosa pack, still alone at time of writing |
| Dispersal | 2AM | 14 December 2019 | Dispersed from Pwadzi pack four days after release from enclosure. One male killed by lion (Table 3) while the remaining male joined 1AF to form Mopane pack |
| Dispersal | 1AF | 16 December 2019 | Dispersed from Gorongosa pack and joined remaining Pwadzi male disperser to form Mopane pack |
| Dispersal | 3AF | 18 December 2019 | Dispersed from Pwadzi pack, still not with males at time of writing |
| Dispersal* | 1AF | May 2020 | Beta female of Pwadzi pack (pregnant) left pack to give birth on her own (raising three successfully to date). Whether the beta female rejoins Pwadzi pack remains to be seen. |

*Technically a dispersal but accompanied by her remaining three pups
