# Supplementary material for: The successful reintroduction of African wild dogs (Lycaon pictus) to Gorongosa National Park, Mozambique
Source: PLoS One. 2021 Apr 22;16(4):e0249860. doi: 10.1371/journal.pone.0249860 (PMC8062010; doi:10.1371/journal.pone.0249860)
Supplement: S4 Table — Observed number and proportion of total kills made by wild dogs and lions in Gorongosa National Park between June 2018 and September 2020. Jacob’s index of selection is also shown where positive values represent preferred while negative values represent avoided. (DOCX) [file pone.0249860.s004.docx]

**S4 Table. Wild dog and lion kills.** Observed number and proportion of total kills made by wild dogs and lions in Gorongosa National Park between June 2018 and September 2020. Jacob’s index of selection is also shown where positive values represent preferred while negative values represent avoided.

| **Prey species & age class** | **Prey population size in 2018** | **Wild dogs** | | **Lions** | |
| --- | --- | --- | --- | --- | --- |
|  |  | **N kills** | **Proportion kills** | **N kills** | **Proportion kills** |
| Bushbuck | 1,665 | 37 | 0.36 | 0 | 0 |
| *Adult* | - | 34 | 0.33 | NA | NA |
| *Calf* | - | 1 | 0.01 | NA | NA |
| *Subadult* | - | 1 | 0.01 | NA | NA |
| *Unknown* | - | 1 | 0.01 | NA | NA |
| Buffalo | 960 | 0 | 0 | 1 | 0.01 |
| *Yearling* | - | NA | NA | 1 | 0.01 |
| Hartebeest | 578 | 0 | 0 | 1 | 0.01 |
| *Adult* | - | NA | NA | 1 | 0.01 |
| Common reedbuck | 10,220 | 13 | 0.13 | 4 | 0.04 |
| *Adult* | - | 9 | 0.09 | 3 | 0.03 |
| *Subadult* | - | 3 | 0.03 | 0 | NA |
| *Unknown* | - | 1 | 0.01 | 1 | 0.01 |
| Impala | 6,122 | 11 | 0.11 | 3 | 0.03 |
| *Adult* | - | 9 | 0.09 | 3 | 0.03 |
| *Subadult* | - | 1 | 0.01 | 0 | NA |
| *Unknown* | - | 1 | 0.01 | 0 | NA |
| Kudu | 1,928 | 2 | 0.02 | 0 | 0 |
| *Subadult* | - | 1 | 0.01 | NA | NA |
| *Yearling* | - | 1 | 0.01 | NA | NA |
| Nyala | 1,934 | 2 | 0.02 | 2 | 0.02 |
| *Adult* | - | 1 | 0.01 | 2 | 0.02 |
| *Subadult* | - | 1 | 0.01 | 0 | NA |
| Oribi | 3,958 | 5 | 0.05 | 0 | 0 |
| *Adult* | - | 5 | 0.05 | NA | NA |
| Sable | 805 | 0 | 0 | 1 | 0.01 |
| *Calf* | - | NA | NA | 1 | 0.01 |
| Warthog | 10,739 | 3 | 0.03 | 69 | 0.64 |
| *Adult* | - | 0 | NA | 52 | 0.48 |
| *Calf* | - | 1 | 0.01 | 0 | NA |
| *Subadult* | - | 0 | NA | 4 | 0.04 |
| *Unknown* | - | 1 | 0.01 | 12 | 0.11 |
| *Yearling* | - | 1 | 0.01 | 1 | 0.01 |
| Wildebeest | 587 | 0 | 0 | 1 | 0.01 |
| *Adult* | - | NA | NA | 1 | 0.01 |
| Waterbuck | 55,711 | 29 | 0.28 | 27 | 0.25 |
| *Adult* | - | 10 | 0.10 | 18 | 0.17 |
| *Calf* | - | 3 | 0.03 | 1 | 0.01 |
| *Subadult* | - | 7 | 0.07 | 5 | 0.05 |
| *Unknown* | - | 2 | 0.02 | 2 | 0.02 |
| *Yearling* | - | 8 | 0.08 | 1 | 0.01 |
