# Supplementary figures and images for: The successful reintroduction of African wild dogs (Lycaon pictus) to Gorongosa National Park, Mozambique
Source: PLoS One. 2021 Apr 22;16(4):e0249860. doi: 10.1371/journal.pone.0249860 (PMC8062010; doi:10.1371/journal.pone.0249860)

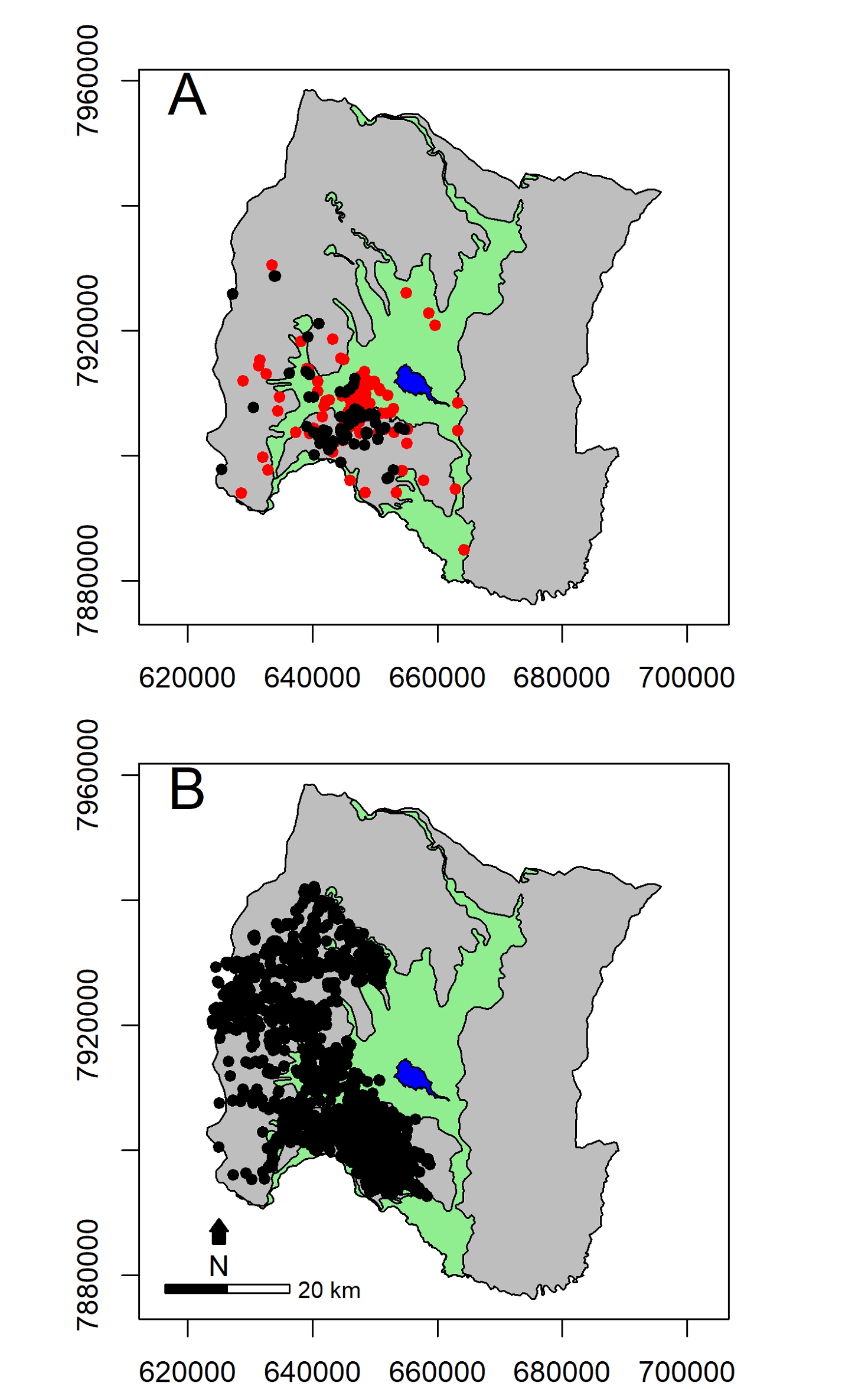

Supplement: S1 Fig — The location of (A) kills for wild dogs (black dots, n = 72) and kills for lions (red dots, n = 89) and (B) wild dog GPS fixes (n = 2,985) in Gorongosa National Park between June 2018 and September 2020, overlaid onto vegetation type (woodland–grey; floodplain–green), also showing Lake Urema (blue). (TIF) [file pone.0249860.s005.tif]

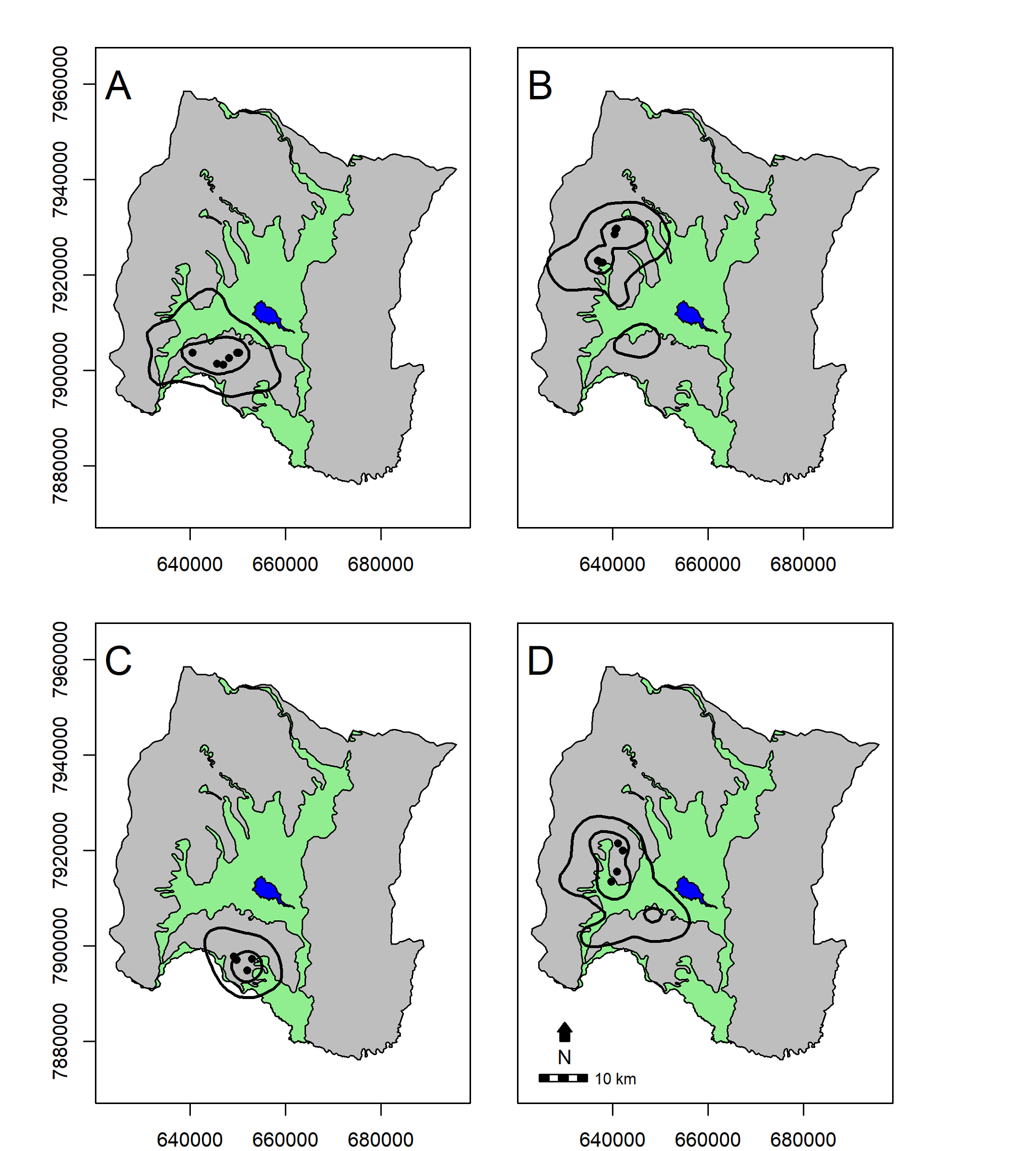

Supplement: S2 Fig — Den sites (black diamonds) used by packs of wild dogs in Gorongosa National Park encompassing the 2018, 2019 and 2020 denning seasons overlaid onto the broad vegetation categories (woodland–grey; floodplain–green) and showing Lake Urema (blue). Panels represent different packs, specifically: (A) Gorongosa pack, (B) Pwadzi pack, (C) Cheza pack and (D) Mopane pack with pack specific territory areas shown as solid black lines, representing the 95% total territory area (outer black lines) and the 50% core territory area (inner black lines). (TIF) [file pone.0249860.s006.tif]

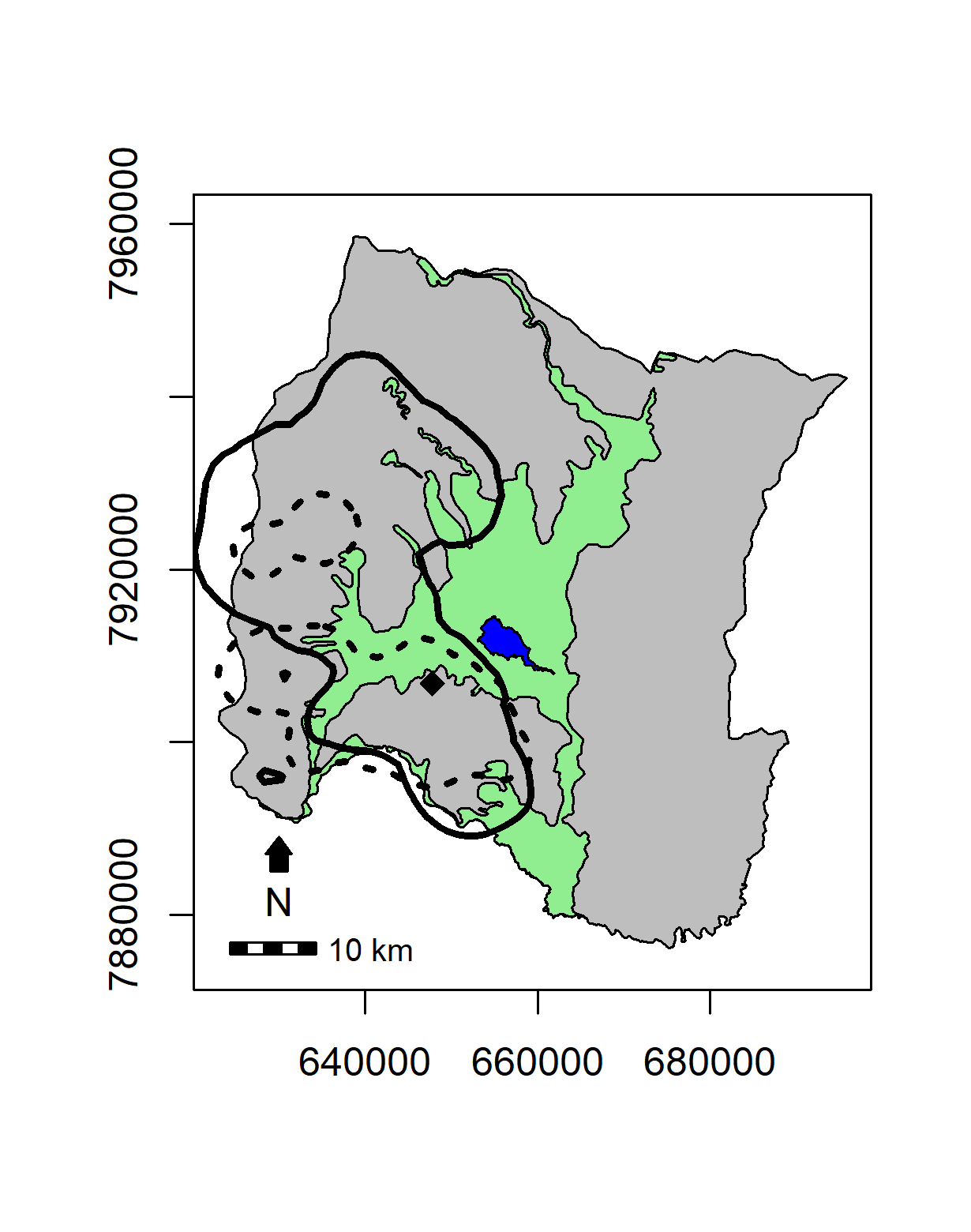

Supplement: S3 Fig — Total areas used by reintroduced wild dogs in Gorongosa National Park during the first 14 months of the study (June 2018 –July 2019, dashed black line) compared to the second 14 months of the study (Aug 2019 –Sep 2020, solid black line). The location of the release enclosure (diamond), Lake Urema (blue), and broad vegetation categories (woodland–grey; floodplain–green) are also shown. The total areas represent the merged 95% kernel UD isopleths from each pack per half of the study period. (TIF) [file pone.0249860.s007.tif]

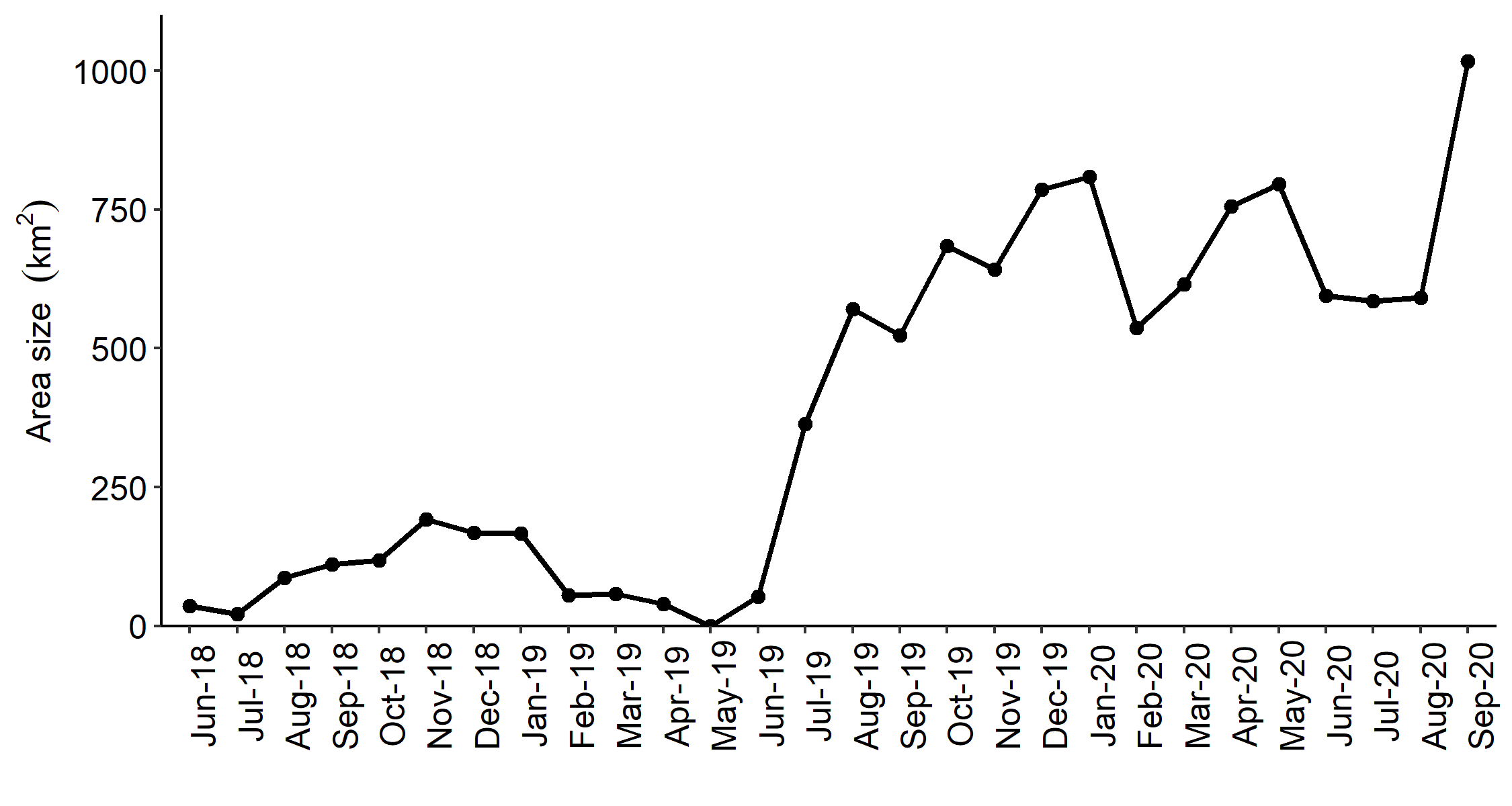

Supplement: S4 Fig — The monthly area size used by the reintroduced wild dogs between June 2018 and September 2020. Area size was delineated by the 95% minimum convex polygon enclosing all GPS fixes for all wild dogs combined. (TIF) [file pone.0249860.s008.tif]
