# Supplementary material for: The successful reintroduction of African wild dogs (Lycaon pictus) to Gorongosa National Park, Mozambique
Source: PLoS One. 2021 Apr 22;16(4):e0249860. doi: 10.1371/journal.pone.0249860 (PMC8062010; doi:10.1371/journal.pone.0249860)
Supplement: S1 Appendix — Interesting observations at wild dog den sites in 2019 and 2020 in Gorongosa National Park. (DOCX) [file pone.0249860.s009.docx]

**S1 Appendix. Den observations.** Interesting observations at wild dog den sites in 2019 and 2020 in Gorongosa National Park.

A skin condition erupted on the beta and gamma females of the Gorongosa pack in mid-June 2020 around the time the beta began denning, sarcoptic manage or Demodex parastites were suspected but could not be verified. It is unclear whether this condition impacted their ability to raise their own pups, but the beta did relocate her pups to the primary alpha’s den 8 days and thereafter she was only observed suckling pups a single time, the alpha was observed allosuckling all pups present routinely. The gamma became increasingly distant from the pack during this same period, and began excavating a den site 2.5km away although retuning daily to the primary den. She was suspected to give birth and desert her pups as they were never seen. Additionally, stiff competition for food between subadults (born in 2019) and new pups (born in 2020) was observed for the Gorongosa pack. While subadults would routinely participate in the twice-daily hunts, once adults returned to the den site to regurgitate food for pups the food was often seen taken by subadults.

Visitations to den sites by adjacent packs or lions impacted pup survival as observed for the Mopane Pack (2 adults) which gave birth to 7 pups. Within days of visitations by other packs (n=6) or lions (n=3) the pack moved den sites, the first time a distance of 1.5km (pups ~ 2mos old) , the second time a distance of 4km (pups ~2.5 mos old), and the third time a distance of 2.6km (pups ~3 mos old). A loss of pups was documented immediately after the 3^rd^ and 4^th^ den moves. By the 4^th^ den move only 4 of the 7 pups had survived. No other lion or neighbouring pack visits were observed on the camera traps at other packs den sites, although we suspect based on postmortem that one adult male from the Cheza pack was killed by a lion close to their first den site in 2020 and just prior to their relocation to their second den site ~1km away, yet all pups (~1.5 mos old) survived this den relocation. Other dangerous wildlife observed at the entrance to or entering various dens included snakes (python, mamba) and warthogs.

Some notable observations of den-site dynamics were made. In 2019, the Gorongosa pack utilized two den sites within 5m of each other and shared by both the alpha- and beta- females that sired pups simultaneously that year. No lions or other disturbances at the den sites were observed. In 2020, the alpha- and beta’s females dens were situated approximately 350m apart, the beta- later moved her pups to the alpha’s primary den. The third pregnant female in the pack began excavating a den site ~2.5km from the primary den where she was presumed to give birth, but she returned to the primary denning area within days and her pups were never seen.

In 2020, the Mopane Pack utilized a total of 3 den sites, and were clearly driven from both the the first and the second den site by visitation by the adjacent Pwadzi pack (denning 3.3km away) and also an adult male lion in the area.
