# Supplementary material for: The successful reintroduction of African wild dogs (Lycaon pictus) to Gorongosa National Park, Mozambique
Source: PLoS One. 2021 Apr 22;16(4):e0249860. doi: 10.1371/journal.pone.0249860 (PMC8062010; doi:10.1371/journal.pone.0249860)
Supplement: Attachment — Submitted filename: PLOS ONE-D-20-35800_Review17Dec2020.pdf [file pone.0249860.s010.pdf]

PLOS ONE

Call of the wild: the successful reintroduction of African wild dogs to Gorongosa National Park, Mozambique

--Manuscript Draft--

**Manuscript Number:**	PONE-D-20-35800
**Article Type:**	Research Article
**Full Title:**	Call of the wild: the successful reintroduction of African wild dogs to Gorongosa National Park, Mozambique
**Short Title:**	Successful reintroduction of African wild dogs (Lycaon pictus) to Gorongosa National Park, Mozambique
**Corresponding Author:**	Paola Bouley<br>Gorongosa National Park<br>Gorongosa, Sofala MOZAMBIQUE
**Keywords:**	Carnivore recovery, Gorongosa National Park, Lycaon pictus, natural population dynamics, rewilding, restoration
**Abstract:**	Large carnivores are important components of healthy ecosystems. However, carnivores are threatened globally, surviving mostly across fragmented ranges because of widespread extirpations. The effect of the loss of large carnivores has been prominent in Gorongosa National Park, Mozambique given most of the large carnivore guild was lost during the 1977-92 civil war. To restore the guild of large carnivores to Gorongosa, reintroductions are now being implemented with a focus recently on endangered African wild dogs (Lycaon pictus). Here, we describe the transboundary translocation and reintroduction of two packs of wild dogs to Gorongosa over a 28-month period and use data on artificial group formation, demography, diet, space use and management to evaluate the success of the reintroduction based on 16 indicators. We artificially formed packs in holding enclosures prior to release which aided in group cohesion. Post-release, we also observed natural pack dynamics with dispersal resulting in new pack formations. Packs bred in two of the three breeding seasons, recruited new individuals, and exhibited a 73% survival rate with no human-related mortalities. Wild dogs also avoided lions and effectively hunted preferred prey. Overall, we show that 15 of the 16 indicators were fully achieved (one partially achieved) and that the reintroduction was successful. This represents the first successful reintroduction of wild dogs into an open landscape. We expect further the population expansion based on extensive vacant areas (68% of Gorongosa unused by wild dogs) and suggest that this expansion could be further stimulated by continued reintroductions over the short-term. Such an expansion has the potential to establish a large and connected wild dog population across the greater Gorongosa-Marromeu landscape which has important regional implications for the species. We also suggest that our study can be used as framework for evaluating future wild dog reintroductions and applied to other carnivore reintroductions.
**Order of Authors:**	Paola Bouley
	David G. Marneweck
	Paulo Antonio
	Mercia Angela
**Additional Information:**	
**Question**	**Response**
**Financial Disclosure**<br><br>Enter a financial disclosure statement that describes the sources of funding for the work included in this submission. Review the submission guidelines for detailed	The author(s) received no specific funding for this work.The author(s) received no specific funding for this work.

Powered by Editorial Manager® and ProduXion Manager® from Aries Systems Corporation

requirements. View published research articles from *PLOS ONE* for specific examples.<br><br>This statement is required for submission and **will appear in the published article** if the submission is accepted. Please make sure it is accurate.<br><br>**Unfunded studies**<br>Enter: *The author(s) received no specific funding for this work.*<br><br>**Funded studies**<br>Enter a statement with the following details:<br>• Initials of the authors who received each award<br>• Grant numbers awarded to each author<br>• The full name of each funder<br>• URL of each funder website<br>• Did the sponsors or funders play any role in the study design, data collection and analysis, decision to publish, or preparation of the manuscript?<br>• **NO** - Include this sentence at the end of your statement: *The funders had no role in study design, data collection and analysis, decision to publish, or preparation of the manuscript.*<br>• **YES** - Specify the role(s) played.<br><br>* typeset	
**Competing Interests**<br><br>Use the instructions below to enter a competing interest statement for this submission. On behalf of all authors, disclose any competing interests that could be perceived to bias this work—acknowledging all financial support and any other relevant financial or non-financial competing interests.<br><br>This statement **will appear in the published article** if the submission is accepted. Please make sure it is accurate. View published research articles from *PLOS ONE* for specific examples.	The authors have declared that no competing interests exist.The authors have declared that no competing interests exist.

NO authors have competing interests

Enter: *The authors have declared that no competing interests exist.*

Authors with competing interests

Enter competing interest details beginning with this statement:

I have read the journal's policy and the authors of this manuscript have the following competing interests: [insert competing interests here]

* typeset

Ethics Statement

Enter an ethics statement for this submission. This statement is required if the study involved:

- Human participants
- Human specimens or tissue
- Vertebrate animals or cephalopods
- Vertebrate embryos or tissues
- Field research

Write "N/A" if the submission does not require an ethics statement.

General guidance is provided below. Consult the submission guidelines for detailed instructions. **Make sure that all information entered here is included in the Methods section of the manuscript.**

All field work was conducted in accordance with the American Society of Mammalogists guidelines (Sikes and Mammalogists, 2016) and approved by the University of Pretoria’s Ethics Committee (EC018-18). The field work was also approved by Gorongosa National Park under the research permit number PNG/DSCi/C108/2018. Transboundary translocations were undertaken for the reintroductions and involved a number of international clearances and permits. The 2018 translocation was permitted under import permits that included a Mozambican Conservation certificate (no. 0004/2018) and a Mozambique State Veterinary certificate (no. 02AB/DEV/2018) as well as exports permits from South Africa including an Ezemvelo Kwazulu Natal Wildlife permit (no. OP 1544/2018) and South Africa State Veterinary permit (no. 045312). Similarly, in 2019 the translocation was permitted under a Mozambican Conservation certificate (no. 00265/2019), a Mozambican State Veterinary certificate (no. 27AB/DEV/2018), a South African North West Province Permit (no. NW 14879/10/2019) and South African State Veterinary permit (no. 018535). All field work was conducted in accordance with the American Society of Mammalogists guidelines (Sikes and Mammalogists, 2016) and approved by the University of Pretoria’s Ethics Committee (EC018-18). The field work was also approved by Gorongosa National Park under the research permit number PNG/DSCi/C108/2018. Transboundary translocations were undertaken for the reintroductions and involved a number of international clearances and permits. The 2018 translocation was permitted under import permits that included a Mozambican Conservation certificate (no. 0004/2018) and a Mozambique State Veterinary certificate (no. 02AB/DEV/2018) as well as exports permits from South Africa including an Ezemvelo Kwazulu Natal Wildlife permit (no. OP 1544/2018) and South Africa State Veterinary permit (no. 045312). Similarly, in 2019 the translocation was permitted under a Mozambican Conservation certificate (no. 00265/2019), a Mozambican State Veterinary certificate (no. 27AB/DEV/2018), a South African North West Province Permit (no. NW 14879/10/2019) and South African State Veterinary permit (no. 018535).

Powered by Editorial Manager® and ProduXion Manager® from Aries Systems Corporation

**Format for specific study types**<br><br>**Human Subject Research (involving human participants and/or tissue)**<br>• Give the name of the institutional review board or ethics committee that approved the study<br>• Include the approval number and/or a statement indicating approval of this research<br>• Indicate the form of consent obtained (written/oral) or the reason that consent was not obtained (e.g. the data were analyzed anonymously)<br><br>**Animal Research (involving vertebrate animals, embryos or tissues)**<br>• Provide the name of the Institutional Animal Care and Use Committee (IACUC) or other relevant ethics board that reviewed the study protocol, and indicate whether they approved this research or granted a formal waiver of ethical approval<br>• Include an approval number if one was obtained<br>• If the study involved *non-human primates*, add *additional details* about animal welfare and steps taken to ameliorate suffering<br>• If anesthesia, euthanasia, or any kind of animal sacrifice is part of the study, include briefly which substances and/or methods were applied<br><br>**Field Research**<br><br>Include the following details if this study involves the collection of plant, animal, or other materials from a natural setting:<br>• Field permit number<br>• Name of the institution or relevant body that granted permission	
**Data Availability**<br><br>Authors are required to make all data underlying the findings described fully available, without restriction, and from the time of publication. PLOS allows rare exceptions to address legal and ethical concerns. See the PLOS Data Policy and FAQ for detailed information.	Yes - all data are fully available without restriction; Yes - all data are fully available without restriction

Powered by Editorial Manager® and ProduXion Manager® from Aries Systems Corporation

A Data Availability Statement describing where the data can be found is required at submission. Your answers to this question constitute the Data Availability Statement and **will be published in the article**, if accepted.<br><br>**Important:** Stating 'data available on request from the author' is not sufficient. If your data are only available upon request, select 'No' for the first question and explain your exceptional situation in the text box.<br><br>Do the authors confirm that all data underlying the findings described in their manuscript are fully available without restriction?	
**Describe where the data may be found in full sentences. If you are copying our sample text, replace any instances of XXX with the appropriate details.**<br><br>• If the data are **held or will be held in a public repository**, include URLs, accession numbers or DOIs. If this information will only be available after acceptance, indicate this by ticking the box below. For example: *All XXX files are available from the XXX database (accession number(s) XXX, XXX.*).<br>• If the data are all contained **within the manuscript and/or Supporting Information files**, enter the following: *All relevant data are within the manuscript and its Supporting Information files.*<br>• If neither of these applies but you are able to provide **details of access elsewhere**, with or without limitations, please do so. For example:<br><br>*Data cannot be shared publicly because of [XXX]. Data are available from the XXX Institutional Data Access / Ethics Committee (contact via XXX) for researchers who meet the criteria for access to confidential data.*<br><br>*The data underlying the results presented in the study are available from (include the name of the third party*	The data underlying the results presented in the study are available from FigShare[illegible]e data underlying the results presented in the study are available from FigShare.

and contact information or URL).<br>• This text is appropriate if the data are owned by a third party and authors do not have permission to share the data.<br><br>* typeset	
Additional data availability information:	Tick here if the URLs/accession numbers/DOIs will be available only after acceptance of the manuscript for publication so that we can ensure their inclusion before publication.; Tick here if the URLs/accession numbers/DOIs will be available only after acceptance of the manuscript for publication so that we can ensure their inclusion before publication.

Powered by Editorial Manager® and ProduXion Manager® from Aries Systems Corporation

Manuscript

Click here to access/download;Manuscript;Manuscript_Final_Submission.do

Call of the wild: the successful reintroduction of African wild dogs (*Lycaon pictus*) to Gorongosa National Park, Mozambique

Paola Bouley[1*¶], Antonio Paulo[1¶], Mercia Angela[1¶], Cole Du Plessis[2¶], David G. Marneweck[3¶]

[1]Department of Conservation, Wildlife Management, Gorongosa National Park, Mozambique

[2]Endangered Wildlife Trust, Johannesburg, South Africa

[3]Wildlife Ecology Lab, School of Natural Resource Management, Nelson Mandela University, George, South Africa

*Corresponding author

Email: seagoose1@protonmail.com

¶These authors contributed equally to the work

Abstract

Large carnivores are important components of healthy ecosystems. However, carnivores are threatened globally, surviving mostly across fragmented ranges because of widespread extirpations. The effect of the loss of large carnivores has been prominent in Gorongosa National Park, Mozambique given most of the large carnivore guild was lost during the 1977-92 civil war. To restore the guild of large carnivores to Gorongosa, reintroductions are now being implemented with a focus recently on endangered African wild dogs (*Lycaon pictus*). Here, we describe the transboundary translocation and reintroduction of two packs of wild dogs to Gorongosa over a 28-month period and use data on artificial group formation, demography, diet, space use and management to evaluate the success of the reintroduction based on 16 indicators. We artificially formed packs in holding enclosures prior to release which aided in group cohesion. Post-release, we also observed natural pack dynamics with dispersal resulting in new pack formations. Packs bred in two of the three breeding seasons, recruited new individuals, and exhibited a 73% survival rate with no human-related mortalities. Wild dogs also avoided lions and effectively hunted preferred prey. Overall, we show that 15 of the 16 indicators were fully achieved (one partially achieved) and that the reintroduction was successful. This represents the first successful reintroduction of wild dogs into an open landscape. We expect further the population expansion based on extensive vacant areas (68% of Gorongosa unused by wild dogs) and suggest that this expansion could be further stimulated by continued reintroductions over the short-term. Such an expansion has the potential to establish a large and connected wild dog population across the greater Gorongosa-Marromeu landscape which has important regional implications for the species. We also suggest that our study can be used as framework for evaluating future wild dog reintroductions and applied to other carnivore reintroductions.

Key words: carnivore recovery, Gorongosa National Park, *Lycaon pictus*, natural population dynamics, rewilding, restoration

Introduction

Large carnivores are crucial to biodiversity in that they aid in the structure and functioning of healthy ecosystems [1-4]. Carnivores directly affect primary consumers through predation [5-8] and indirectly affect herbivores through creating a “landscape of fear” of predation [9, 10] which can filter down to affect primary producers in multiple ways [1]. Carnivores provide essential ecosystem services and help focus sustainable development initiatives surrounding protected areas [11] and also have a high potential to contribute positively to local livelihoods through ecotourism [12-14].

Despite their ecological and socio-economic importance, large carnivores remain highly threatened [2, 15]. Most carnivore species are listed as threatened or endangered with 77% experiencing population declines due to habitat loss [2, 16], depleted prey populations [17], direct killing by humans and human-carnivore conflict [15, 18, 19]. Some of the largest range contractions of carnivores have occurred on the African continent [16], leaving fragmented patches of suitable habitat. A range of proactive conservation strategies can be implemented to mitigate these threats in order to promote population recovery. These are mainly targeted at re-establishing viable habitat corridors between suitable patches through promoting human-carnivore coexistence to ensure connectivity amongst populations. Where extirpations have occurred and where connectivity between populations is unlikely, reintroductions (i.e. conservation translocations) of carnivores remains a viable tool to promote population recovery [20-24].

The African wild dog (*Lycaon pictus*) (aka painted wolf, painted dog, Mabeco) historically occurred across much of sub-Saharan Africa [25, 26] having evolved as an integral

part of ecosystems over ~3.9 million years [27]. Currently the species is restricted to less than seven percent of its historical range [28] and is listed as endangered by the IUCN [26]. Wild dogs have undergone precipitous and sustained declines over the past four-decades driven largely by exponential human population growth, severe habitat loss and fragmentation [26, 29] and being subject to bycatch in an escalating illegal bushmeat trade [30]. Ecologically, wild dogs are considered large carnivores [2, 31] and play important top-down roles in regulating prey populations [32]. As the only true coursing large predator in sub-Saharan Africa, wild dogs play an important role in ensuring fit populations of their prey by foraging on old, young and vulnerable individuals [33]. Consequently, wild dogs play a unique role from within the large carnivore guild and are critically important in healthy ecosystems in Africa. It is therefore imperative to strengthen protection of extant populations wild dogs and where possible to restore and re-connect populations.

The recently restored Gorongosa National Park (GNP), Mozambique's flagship national park, represents one such opportunity for the species' recovery. Historically a renowned stronghold for wildlife, GNP was contemporaneous with the Serengeti and Ngorongoro Crater ecosystems and supported a high biomass of wildlife [34, 35]. Unfortunately, the system underwent a severe perturbation at the epi-center of the 1977-92 civil-war that resulted in a >90% decline in populations of large mammals and carnivores [36, 37]. In 2007, a long-term, public-private ecosystem restoration initiative - "the Gorongosa Restoration Project (GRP)" - was established with the goals of restoration of wildlife populations, post-war recovery of the region's park-based economy, and improving the quality of life for human communities surrounding GNP [38]. Large herbivore populations have since recovered with a 95% recovery in biomass [34] and the indigenous lion (*Panthera leo*) population is also recovering [39]. This multi-trophic level recovery paired with strengthened management efforts ensuring close monitoring and security for wildlife in GNP, increased

interconnectivity of habitat across the contiguous 20,000 km of the greater Gorongosa-Marromeu landscape which includes the park, and a sustained human development and human-wildlife coexistence strategy laid the foundation for a step-wise program to reintroduce extirpated carnivores, commencing with wild dogs in 2018.

The primary aim of this study was to document the first two-years of the reintroduction of wild dogs to GNP. We focused on describing and analyzing the effectiveness of artificial group formation in pre-release enclosures and their subsequent post-release demography, diet and space use. Our secondary aim was to use these data to evaluate if the reintroduction had been successful at a short-term scale [40] and to formalize the baseline against which to monitor progress over future years. We defined broad goals of success that included artificial formation of packs, natural pack formation dynamics, reproduction and recruitment, survival, diet, packs establishing home-ranges, hunting, and a limited impact of humans on individual survival. We outline a range of key biological, ecological and management indicators of short-term reintroduction success (Table 1) [40, 41] and discuss if the reintroduction of wild dogs to GNP has been successful.

Table 1. Framework for evaluating short-term success of the wild dog reintroduction to Gorongosa National Park. Indicators and their references are taken from multiple species accounts in the in the IUCN Global Reintroduction Perspectives Reports [42-47] while we also include carnivore and/or wild dog specific references for indicators.

**Category**	**Indicator [References]**
1. Artificial group formation	1A. Founder males and founder females bond as a new pack/s and the majority of these founders remain together post-release [22, 48]
	1B. Alpha pair established in each pack [49]
	2A. Dispersal cohorts from founder packs [22, 40, 50, 53, 54]

2. Natural pack formation	2B. Dispersal results in formation of new packs reflecting natural population dynamics [40, 50, 56, 67, 68, 73]
3. Breeding	3A. Packs breed annually as evidenced through seasonal denning and siring of at least one litter per pack per season [21, 24, 51, 53, 55, 68-70, 73, 74]
4. Survival	4A. Packs recruit members through raising pups to one year old [24, 51, 53, 55, 66, 73, 74]
	4B. Survival of 50% of founders (i.e. positive population growth) [23, 24, 53, 55, 64, 65, 73, 74]
5. Population size	5A. Population size increases significantly over time [23, 24, 53, 73, 74]
6. Diet	6A. Packs select prey of small-medium weight range (i.e. 16-32 kg and 120-140 kg) [61]
7. Space use	7A. Population expands its range use over the study period [23, 24, 55, 65]
	7B. Packs form distinct territories made up of small cores of intensive use and outer areas of infrequent use [24, 50, 65, 68, 71, 72]
8. Lion co-existence	Packs do at least one of these:
	8A. Packs avoid lion preferred prey [61, 63] AND/OR
	8B. Packs avoid areas intensively used by lions [50, 57-59]
9. Human	9A. Packs avoid high human-density areas [60]
	9B. Minimal human-induced mortality events in GNP, e.g. snares, livestock predation, disease, road-kills, direct killing, poisoning [40, 52, 55]
10. Management	10A. Monitoring and evaluation of biological data is collected locally to inform adaptive management decisions [24, 40, 41, 62]
	10B. Trained wildlife management personnel and wildlife veterinarians of national origin overseeing the reintroduction and recovery process [24, 41, 62]

Materials and methods

Study Site

The Gorongosa-Marromeu landscape located in Central Mozambique (Fig 1) is composed of a highly diverse mosaic of habitats [75, 76]. This landscape is managed as a

contiguous 20,000 km^2 complex of national park, national reserve, buffer-zones, forest reserves and hunting concessions or "coutadas" (Fig 1). Designated as a potential stronghold for lions [77, 78], the Gorongosa-Marromeu landscape, is also habitat for leopard (*Panthera pardus*), spotted hyenas (*Crocuta crocuta*) and wild dogs which were all present pre-civil war [35] but are currently ephemeral, absent or occurring at very low densities.

The unfenced, 4,067 km^2 GNP is situated in the core of a contiguous landscape of protected areas known as "Gorongosa-Marromeu" (Fig 1; -18.97898°, 34.35190°) and positioned at the southern edge of the Great Rift Valley. The park's vegetation types (not including the separate Gorongosa Mountain) can be broadly classified into woodland and floodplain with the 18 km^2 Lake Urema at its center. GNP is sub-tropical and characterized by wet summers (November-April) and dry winters (May-October). The current herbivore biomass density in GNP is currently ~8,800 $kg.km^{-2}$ or approximately 95% of pre-war levels. Of 20 focal small- to mega-sized herbivores species such as oribi (*Ourebia ourebi*), warthog (*Phacochoerus africanus*), bushbuck (T*ragelaphuss ylvaticus),* nyala (*T. angasii*), kudu (*T. strepsiceros*), buffalo (*Syncerus caffer*) and others, composition is currently dominated by a meso-herbivore, the waterbuck (*Kobus ellipsiprymnus*) [34]. Pre- the 1977-92 civil war, a range of carnivores were documented in GNP including *C. Crocuta*, *P. pardus*, *P. leo* and *L. pictus* [35]. Beginning in 2012, GNP launched a long-term, intensive survey of extant carnivore populations. With the exception of ephemeral sightings of a lone spotted hyaena (2012) and leopard (2018/19), an indigenous population of lions was all that remained in GNP [39].

Fig 1. The location of the Gorongosa-Marromeu landscape (bottom panel) within the Sofala province of central Mozambique (black shading in top right panel) on the east coast of Africa (black shading in top left panel). In the bottom panel, the extent of different protected areas are shown with solid lines indicating official protected area status and dashed lines for buffer-zones

and adjacent forestry areas overlaid on human population density (number per 1 km^2). Letters refer to (A1) Mountain section of Gorongosa National Park, (A2) core area of Gorongosa National Park, (B) buffer area of Gorongosa National Park, (C) forestry areas, (D) Coutada 12, (E) Coutada 11, (F) Coutada 14, (H) Marromeu National reserve and (G) Coutada 10.

Determining the status of wild dogs in GNP

Wild dogs occurred in GNP prior to the civil war between 1977 and 1992 [35] but were completely extirpated by 1992 likely driven by poaching (snaring, gin-trapping), prey depletion, and instability in governance [79]. An intensive survey of large carnivores commencing in 2012 [39] as well as WildCam Gorongosa camera-trapping surveys (https://www.zooniverse.org/projects/zooniverse/wildcam-gorongosa), and the daily tracking of eco-tourism guides in the area, did not yield a single confirmed sighting of wild dogs in GNP between 2012 and 2018. It was concluded that wild dogs were effectively extinct in GNP and had not naturally recolonized from other resident populations in the region.

GNP has a full-time, locally-staffed wildlife management team that includes wildlife rangers, veterinarians, carnivore specialists and data analysts who rigorously track and monitor resources and relevant anthropogenic factors, e.g. snaring pressure and human-wildlife conflict. In 2015, GNP implemented a rigorous restructuring of anti-poaching patrols across the landscape, by 2018 poaching pressure (snares and traps) had reduced by 65% and poaching of lions by 95% (R. Branco, pers comm). GNP managers are therefore confident that threats that the lead to the historical extirpation of wild dogs in GNP have been alleviated.

Reintroduction suitability assessment

Considering that the last wild dogs disappeared during the civil war and that no recolonizations occurred post-civil war despite extensive effort to protect the landscape,

conservation translocations (i.e. reintroductions) were identified as a key conservation strategy [25]. To evaluate if GNP was ecologically suitable and whether it was a viable site for a reintroduction, we implemented a number of data-driven desktop assessments. Firstly, GNP was among a number of sites assessed in 2015 by the Range Wide Conservation Programme for cheetahs and wild dog undertaken to investigate, identify and update resident, possible, transient, connection, recoverable, extirpated and unknown sites across southern Africa [25].

Secondly, a wild dog feasibility assessment designed by the Wild Dog Advisory Group-South Africa (WAG-SA) to guide reintroductions was used to assess the suitability of potential reintroduction sites. Feasibility assessments for the reintroduction of wild dogs in South Africa have been used for over 20 years with success in recovering wild dog range and population density [80]. The WAG-SA assessment incorporates ecosystem size, preferred prey densities, competitor predator densities, disease history, fencing conditions, potential for human-wildlife conflict and sustainable financial support to support the recovery process in to the future (Endangered Wildlife Trust (EWT), unpublished data).

Through these two assessments, GNP was identified as ‘recoverable’ area by the RWCP and a conservation translocation was considered the best option of recovering the population given the unlikely possibility of reconnecting GNP to any other confirmed and no resident populations within the Gorongosa-Marromeu landscape [25]. GNP scored 76 (out of a possible 100) in the WAG-SA Feasibility Assessment (EWT, unpublished data). As GNP is not fenced, the subsequent potential for exposure to disease at the human-wildlife interface being permeable was where GNP was deemed most vulnerable. However, with an average score of 68 among other protected areas in South Africa (EWT, unpublished data) conditions in GNP strongly suggested a reintroduction was feasible. GNP supports abundant herbivore populations [34] and based on the herbivore biomass in GNP, the Hayward carrying capacity model [81] estimated the park could sustain a minimum of ~82 wild dogs or approximately

eight packs assuming average pack size of 10 individuals. Overall, the feasibility assessment suggested that GNP could support a population of wild dogs and a conservation management decision was made to implement a coordinated reintroduction of wild dogs into GNP to recover this population.

Sourcing of founder packs

Due to the success of the metapopulation in South Africa [80], multiple wild dogs were available as surplus packs and individuals. Considering the availability of these individuals and packs and their high levels of genetic diversity [82], wild dogs were sourced from the metapopulation in South Africa.

We conducted the reintroduction in two phases, with each comprising the reintroduction of a single pack. We initiated phase one in 2018 which involved the translocation of nine adult males from the uMkhuze section of iSimangaliso Wetland Park in KwaZulu-Natal (-27.64026°, 32.15859°) and one adult and five yearling females from a free roaming pack in the Magudu area in KwaZulu-Natal (-27.50140°, 31.55088°). The two groups were unrelated [82] and were deemed ideal for phase one for artificial bonding in the pre-release enclosure.

We initiated phase two in 2019 which involved the translocation of a recently naturally formed pack group of nine adult males and six adult females from Khamab Kalahari Game Reserve (-27.33249°, 31.84555°). This pack was genetically distinct from the pack in phase one, comprising lineages from Zimanga Game Reserve in KwaZulu-Natal (-27.5741°, 32.0030°), Madikwe Game Reserve in the North West (-24.7604°, 26.2777°) and from individuals that immigrated into Khamab, assumed to have originated from south-west Botswana.

Translocations

Wild dog reintroductions are more successful with soft-release from holding enclosures than hard releases [21, 24, 40]. Moreover, when artificially forming new packs, unrelated groups of males and females need to undergo a period of pre-release bonding to one another to enhance pack formation and increase short-term and long-term success [48]. Using small (~5,000m^2) and electrified holding areas and an olfactory acclimatization technique [49], bonding between unrelated opposite sex groups can be enhanced. Thus, we constructed a permanent single compartment 5,000 m^2 enclosure with ample shade-cover, a shallow swimming area and a water trough to receive, hold, acclimatize and socially integrate groups prior to their soft-release in to the core wilderness area. The enclosure included robust electric fencing to keep the pack inside the fence safe from external predators such as lion, and to prevent translocated individuals from escaping the enclosure prematurely.

Phase one – 2018

We captured the six adult females and nine adult males and transported them into adjacent compartments within the same enclosure at Phongola Nature Reserve, South Africa (-27.33291°, 31.84462°) on the 29th of March 2018. We deliberately placed the unrelated and opposite sex groups adjacent to one another to allow for a controlled group integration [83, 84]. On the 16th of April 2018, we sedated (using Zoletil) and translocated the two groups via air-charter to GNP, a distance of ~1,000km. On arrival at the GNP airfield, we drove the sedated individuals 15 km to the holding enclosure. We collared every wild dog in the pack, comprising two satellite collars and 12 VHF collars. To accelerate the bonding process, we physically rubbed sedated male and female wild dogs to exchange individual scents and build familiarity between the two sex groups with the goal of reducing aggression and to help promote pack cohesion [49]. Unfortunately, one of the adult males died in the enclosure on the arrival day of the 16th of April 2018, cause unknown.

Phase two – 2019

We captured and translocated the pack of nine adult males and six females from Khamab Kalahari Reserve on the 5th of November 2019. We translocated the sedated pack via air-charter to GNP and transferred them upon arrival to the 5,000 m^2 enclosure where 14 of the 15 individuals were fitted with either satellite- or VHF-collars.

During both translocation phases, we compiled individual photographic identification kits that comprised information on each individual's origin, age, current pack and status, collar type, unique identification number and photographs of the left- and right-side coat patterns and distinguishing marks. This allowed all subsequent observations to be related to unique individuals. Additionally, for both phases, we administered rabies and canine distemper virus (CDV) vaccinations to all wild dogs while they were sedated during air-travel.

Captive bonding, monitoring and release

The acclimatization period during which the packs were captive in the enclosure was crucial to facilitate bonding (especially for phase one) and to allow them time to adjust to their new surroundings. We implemented a daily schedule of monitoring and maintenance procedures at the enclosure. During this bonding in the enclosure, we monitored wild dogs twice daily; at first light for two hours and again for two hours prior to sunset, corresponding to their crepuscular periods of activity [49, 83, 84]. During each monitoring session, we recorded the number of individuals in the holding enclosure, group association behavior, aggressive interactions, mating events and hoo-calling while performing maintenance checks at enclosure to reduce the likelihood of escape. We fed the wild dogs every 2-3 days with freshly culled natural prey (most often as impala and waterbuck) and replaced water in the trough every 1-3 days. Importantly, no wild dogs escaped from the enclosure during this period. From the behavioral monitoring sessions at the enclosure, we estimated pack cohesion and acclimatization in order to identify timing of release and were guided by reintroduction time

frames that optimize short-term and long-term success [48]. In the week prior to release, we administered booster vaccinations for CDV via drop-out darts. Packs were passively released from the enclosure via opening the main gate and leaving a distance of 100 m. The first pack (Gorongosa pack) was released on the 15th of June 2018 after a period of eight weeks in their enclosure, while we released the second pack (Pwadzi pack) on the 10th of December 2018 after a period of five weeks. Due to the Pwadzi pack already being formed prior to translocation, they were kept in the enclosure for a shorter period.

Wild dog data collection post-release

We collected and utilized data from our intensive post-release monitoring that covered a total of 28 consecutive months spanning from June 2018 to September 2020. This represents a short-term that covered three consecutive denning seasons and allowed packs enough time to attempt to recruit individuals through reproduction and pup survival. It also allowed enough time for packs to settle into their new area and form territories.

Post-release monitoring

While we attempted to locate the packs daily, this was reliant area on accessibility constrained by flooding, weather and personnel availability. We used the fitted GPS and VHF collars (see Collars and GPS data section below) to locate the reintroduced founders. Once located, wild dogs were observed from a 4x4 vehicle with 1-2 observers recording data. At each sighting, we recorded (1) number, identity, sex and age of individuals, (2) health condition of individuals and any injuries, (3) dominance (known from overmarking, mate-guarding and scent marking), (4) pregnancies, (5) hunting success, and (7) any behavioural interactions with conspecifics and lions. We also relied on opportunistic field ranger and tourist reports to supplement our dataset. As each wild dog is identifiable from unique coat patterns, we utilized photographs and videos from sightings to confirm individuals present. We verified all identities

against the photographic identification kits constructed during translocation phases. At the worst resolution, each individual was seen and recorded at least once per month. Consequently, we built individual and group-level life histories for wild dogs in GNP at a monthly resolution from June 2018 until 30 September 2020, encompassing 28 consecutive months since the first pack was released. We then used the demographic data to assess monthly (1) group composition, (2) group size, (3) reproduction and recruitment, (4) inter-group movement dynamics, (5) survival, (6) mortality, (7) GPS location and (8) kills.

Breeding

We used VHF collars, GPS collars and direct observations to determine the timing and location of denning. Birth females were known from monitoring prior to denning based on direct observations of alpha pairing, mating, visible signs of pregnancy, disappearance underground, not joining pack hunting events and packs returning to the same site twice a day (indicative denning behaviour). Thus, we were able to confidently assign birth to these females while also recording the birth month and coordinates of dens. We estimated litter size by placing camera traps at identified den sites (location determined from GPS collar clustering and direct observations) to record images and videos. We rotated cameras on a weekly to biweekly schedule as batteries permitted. This also allowed us to add photographs from pups (i.e. non-founders) into our individual life history database and in our identification kits where we assigned age and sex based off camera trap images and subsequent direct observations of pups at dens. We generally recorded pups emerging from dens approximately 3-4 weeks after females went underground. This is the age when pups first emerge from underground [68] and we are confident that this represents actual litter size as per other wild dog studies [53, 69, 70, 85, 86]. We also counted the number of surviving pups for each litter to three, six, nine and 12 months as important age milestones to determine the proportion of pups raised. We defined recruitment as the number of pups raised to 12 months old (i.e. yearling) as a proportion of the

population size (adults and yearlings) in the focal month. The proportion of pups surviving to six, nine and 12 months does not include the six 2020 litters because we could not determine survival to these ages at the end of this study.

Population size, survival and mortality

We used three age class categories for wild dogs including pup (< one year old), yearling (1 – 2 years old) and adult (> 2 years old). We defined a pack as a group of wild dogs with at least one adult male and one adult female, dispersal group as a cohort of a single-sex and splits as a mixed sex cohort that permanently left their most recent pack. We generally classified pack and population size as the sum of adults and yearlings unless otherwise stated where the count also included pups. We determined two types of population density; one as the population size per 100 km^2 and the other as the number of packs per 100 km^2 to allow for individual and group-level comparisons with other populations. Through the field data collection, we assigned alive or dead to each individual at the end of each month. From these monthly data of individual fate, we were able to estimate survival as the number of individuals alive at the end of the study relative to the total number of individuals recorded during the 28 months. This method included both founders and non-founders (i.e. pups). For individuals that were not alive at the end of the study, we assigned mortality and the month of mortality as any individual observed to have died and the carcass or collar recovered. If an individual was not confirmed to have died and was not alive at the end of the study, we assigned it as disappeared and estimated disappearance month as one month after last confirmed observation. For confirmed mortalities, we coded the cause as human (road accident, indirect snaring, direct shooting), natural (predation, intra-specific, hunting) or disease (rabies, distemper) as defined by Woodroffe, Davies-Mostert (52). The sex and age class of dead and disappeared individuals were also recorded. We also recorded changes in group association as evidenced by successful

dispersal (i.e. natural pack formation with opposite sex group), unsuccessful dispersal (have yet to form new pack) and splits.

Diet

We recorded all successful hunts or located carcasses killed by wild dogs. At each kill or carcass, we recorded the prey species and age class (where possible). We used the GNP biennial herbivore census data to determine the amount of available prey biomass [87]. We then applied Jacob's Index of selection [88] to assess prey selection by wild dogs. We separated waterbuck biomass into two distinct categories related to their distribution in GNP: (i) grassland/woodland waterbuck and (ii) floodplain waterbuck because of the large difference in waterbuck biomass relative to each habitat type [87]. We also used lion kill data between 2012 and 2020 (available from the GNP Carnivore Recovery Unit) to assess lion prey selection using Jacob's Index of selection which allowed us to contrast prey selection between wild dogs and lions.

Collars and GPS data

We fitted 42 collars on 30 different individual wild dogs during this study. Individuals were fitted with collars while sedated during the translocation phase while individuals were collared post-release by free-darting from 4x4 vehicles at a range of 20- 35-m and sedated using a combination of Metedomidine and Ketamine. Overall, we fitted 28 GPS (24x African Wildlife Tracking; 2x Sirtrack Iridium; 2x Wildtrack) and 14 VHF collars (6x African Wildlife Tracking; 8x Animal TrackEm). Thus, multiple individuals per pack were collared simultaneously. However, we only used GPS data from one individual per pack at any time to reduce pseudoreplication. We also attempted not to select collar data from breeding females to avoid small and non-representative territories during denning as breeding females tend to stay near the den for large portions of the denning season [68]. Consequently, we included data from nine of the 30 collared individuals (S1 Table). GPS collar fix schedule varied per collar

recording optimally six fixes per day. This full dataset contained 7,944 GPS fixes. We truncated this dataset, to include a maximum of four fixes per group per day, representing two resting periods and two movement periods, each separated by at least six hours. We considered these temporal intervals as adequate to avoid spatio-temporal auto-correlation between consecutive locations without losing relevant information on the ecology of the animals [68]. This truncated dataset included 3,048 fixes with a mean number of 339 fixes per group (S1 Table).

Lion spatial data

Lions pose the largest natural threat to wild dogs through direct interference competition [52, 68, 89, 90] that can limit wild dog populations [68, 91] or even contribute to local extirpation [92, 93]. GNP has a recovering and growing population of lions [39]. Thus estimating how the top competitors for wild dogs are using space is key to understanding wild dog space use that centres on lion avoidance while optimising resource selection for enhancing fitness [57-59]. To estimate lion spatial extent in GNP, we used lion GPS collar data collected by the GNP's Carnivore Recovery Unit between June 2018 and September 2020. We used GPS data from 14 groups of lions (5 prides, 4 male coalitions and 5 dispersal groups), all fitted with African Wildlife Tracking satellite collars (S1 Table). These collared lions account for approximately 10% of the population and 68% of the extent of area used by lions in the park (P. Bouley, unpublished data) and we acknowledge there are uncollared individuals/groups within GNP over this study period. Lion collars were programmed to record fixes every 2-4 hours and we truncated the lion GPS dataset in the same way we did for the wild dogs to reduce pseudoreplication and ensure sampling in the same time periods as the wild dog population to enable meaningful analyses.

Territory estimation

KUDs and territory size

We used the truncated spatial dataset for wild dogs and lions (S1 Table) to estimate territories using Kernel Utilisation Distributions (KUDs). We defined KUDs with a pixel size of 1 km^2 based on bivariate kernels using the *kernelUD* function from *adehabitatHR* package in R. We estimated our smoothing parameter as h = 2,100 based on other wild dog studies that selected h = 2,400 m [71, 72] based on the Silverman (94) suggestion of 0.5 X h, with h = 4,800 m for GPS data. We used this same smoothing parameter value for the lion KUDs. For wild dogs, we estimated group-level KUDs for the first 14 months (June 2018-July 2019), for the second 14 months (August 2019-Sepetmeber 2020) and for the entire 28-month study period. We used the *getverticeshr* function in *adehabitatHR* package in R and specified the WGS84 geographic projection to extract the area size (km^2) enclosed by the 95% and 50% isopleths extracted from KUDs for each half of the study and for the entire study period for wild dogs. For lions, we only created KUDs for the entire study period and the extracted the 95% and 50% isopleths. The 95% isopleths represented the total territory area (excluding 5% of outliers) and the 50% isopleth represented the core territory area of intensive use.

Range expansion

To determine if the wild dog population increased its range over time, we merged the extent of the group-level 95% isopleths for the first 14 months of the study period and for the second 14 months of the study period. We also estimated the proportion of the park used by wild dogs using the merged 95% isopleths for each group covering the entire study period. We also developed monthly 95% minimum convex polygons (MCPs) for packs using the *mcp* function from the *adehabitatHR* package v0.4.15 [95]. We did this to represent the total extent of the area covered by wild dogs without worrying about use.

Overlap area

We assessed spatial interactions between neighbouring wild dog dyads using overlap as an index for territoriality [96, 97]. We estimating overlap area (km^2) between a pack's range for each neighbouring dyadic association to determine what proportion of each focal range overlapped with a neighbour's. We estimated this for both the 95% and 50% isopleths extracted from the KUDs created over the whole study period. We used the merged 95% and 50% isopleths for wild dogs and the merged 95% and 50% isopleths for lions to estimate the amount of overlap between these two carnivores. We did this by relating the proportion of overlap in area size between the 95% and 50% wild dog territories to that of the 95% and 50% lion territories.

Habitat use

We determined the number of fixes per wild dog and lion group from the truncated dataset that fell within each of the two broad vegetation categories (woodland or floodplain). From this, we determined the proportion of fixes for each carnivore population that fell within each vegetation type. We were thus able to contrast species-specific habitat use. We excluded fixes that fell outside the official protected area boundary of the park for the habitat use analysis. We also determined the number of wild dog dens in each of the two broad vegetation types. Some female wild dogs denned in the same site as other females in the pack (S2 Table) and we did not include these duplicates in this analysis (i.e. n = 23 unique den locations).

Ethics and permit numbers

All field work was conducted in accordance with the American Society of Mammalogists guidelines [98] and approved by the University of Pretoria's Ethics Committee (EC018-18). The field work was also approved by Gorongosa National Park under the research permit number PNG/DSCi/C108/2018. Transboundary translocations were undertaken for the reintroductions and involved a number of international clearances and permits. The 2018

translocation was permitted under import permits that included a Mozambican Conservation certificate (no. 0004/2018) and a Mozambique State Veterinary certificate (no. 02AB/DEV/2018) as well as exports permits from South Africa including an Ezemvelo Kwazulu Natal Wildlife permit (no. OP 1544/2018) and South Africa State Veterinary permit (no. 045312). Similarly, in 2019 the translocation was permitted under a Mozambican Conservation certificate (no. 00265/2019), a Mozambican State Veterinary certificate (no. 27AB/DEV/2018), a South African North West Province Permit (no. NW 14879/10/2019) and South African State Veterinary permit (no. 018535).

Statistical analyses

While we generally provide descriptive statistics in our study, we performed non-parametric tests to evaluate some relationships. We used linear regression models to test (1) the effect of year (2018, 2019 and 2020) on population size (adults, yearlings and pups combined), (2) the effect of year (2018, 2019 and 2020) on the number of packs, and (3) the effect of month-year on the MCP size for all dogs combined (to see if there was an asymptote or not). We constructed and evaluated these models using the *lm* function in RStudio.

We used Chi-squared tests to investigate (1) the effect of month on the number of litters observed, (2) the proportion of pups surviving to 3, 6, 9 and 12 months and (3) the effect of group type (founder vs non-founder) on the proportion of animals alive at the end of the study. We also used Chi-squared tests to investigate the effect of vegetation type (woodland versus floodplain) on (4) the number of wild dog GPS fixes in each habitat type, (5) number of wild dog kills in each habitat type and (6) the number of lion GPS fixes in each habitat type. We then used Chi-squared tests to see determine if there were differences in wild dog versus lion use of (7) woodland vegetation and (8) floodplain vegetation and (9) the effect of vegetation type (woodland versus floodplain) on den site location.

We performed all statistical analyses and constructed all figures in the RStudio GUI, desktop version 1.1.456 [99] that used R version 3.5.1 [100] for Windows, using functions in the *stats v3.5.1* package [99], *ggplot2 v3.1.0* package [99] and we plotted maps using the *plot* function from *graphics* base package [99].

Results

Artificial group formation

Gorongosa pack

We observed a female born in 2015 (Beira) mating with a male born in 2015 (Mutondo) within two weeks of being together in the enclosure (i.e. at the end of April 2018). These were the two oldest individuals in the enclosure. These two continued mating into the first 10 days of May 2018. Additionally, two males born in 2016 (Mussapassua and Mini-mini) were observed mating with the same 2015 female (Beira) on the 9th, 10th and 12th-14th May. The males thereafter were observed to separate in two groups within the enclosure (one of five and the other of three) with the larger group containing the older 2015 male (Mutondo); this larger group also contained the females. One fight ensued on 11 May 2018 between the older and younger groups of males appearing to battle for dominance, but little aggression was observed thereafter. Subsequently, we observed the close association of the 2015 female (Beira) and the 2015 male (Mutondo) with consistent overmarking of each other’s scent marks and resting together. This indicated the formation of an alpha pair (indicator 1B, Table 1). It was noted during the week preceding release that the 2015 female (Beira) was pregnant. The eight males and six females remained together after their release from the enclosure (15 June 2018), forming the Gorongosa pack (indicator 1A, Table 1). Despite a subset of individuals that split from the Gorongosa pack months after their release (see Natural pack formation section below), three females and four males of the original founders have remained together in the original Gorongosa pack.

Pwadzi pack

We did not observe signs of aggression during their entire period in the enclosure and we observed close association between one female (Matenga) and one male (Nhamaguena) that suggested the formation of an alpha pair (indicator 1B, Table 1). We did not observe mating during this group's enclosure period. In contrast to the Gorongosa pack, post-release the 15 individuals only stayed together for four days before the first of two dispersal events occurred (see Natural pack formation section below). Despite the dispersals, by the end of this study period, one female and seven males of the original founders have remained together in the Pwadzi pack (indicator 1A, Table 1).

Natural pack formation

We documented 12 of the 29 founder individuals change their group association (Fig 2; S3 Table). This was in the form of dispersals (n = 4 events) (indicator 2A, Table 1) and a split (n = 1 event) from artificially formed packs that resulted in the formation of two new packs (Fig 2; S3 Table) (indicator 2B, Table 1). We observed one subordinate female from the Pwadzi pack to leave the pack when she started denning in May 2020 (Fig 2; S2 and S3 Tables). While this is technically a dispersal, this female is currently only accompanied by her remaining pups and it remains to be seen if she rejoins Pwadzi pack (S2 and S3 Tables)

Fig 2. Group association dynamics of the reintroduced wild dog founders in Gorongosa National Park between June 2018 and September 2020. The reintroduction of two founder packs (Gorongosa and Pwadzi) resulted in two further pack formations, two successful dispersals and three ongoing dispersal events (one of these is a female with her pups she had after dispersing). The number of males and females in Gorongosa and Pwadzi packs show the pack size at the time of release.

Breeding

We recorded 11 pregnancies from seven different females but we only confirmed nine litters. For the two unconfirmed litters, the pups never emerged from underground dens. We recorded these 11 pregnancies in each of the denning seasons in 2018, 2019 and 2020, but only recorded [illegible]ers emerging in the 2019 and 2020 seasons (indicator 3A, Table 1). Mean litter size for the nine confirmed lit[illegible] was 9.11 ± SE1.06 (range 5 – 15). Birth was highly seasonal, with all litters reco[illegible]d between May and July ($\chi^2_{(11)}$ = 51.07, p < 0.001). Multiple maternities per pack was standard for this population with seven of the nine confirmed litters being born when more than one female per pack gave birth (S3 Table).

Survival

We observed 82 pups born (i.e. non-founders) in the park over two consecutive breeding seasons in 2019 and 2020 (indicator 3A, Table 1). Of the 82 pups born, we observed 62 to survive to three months of age (i.e. 76% survival), 23 survive to six months of age (i.e. 28% survival), 23 survive to nine months of age (i.e. 28% sur[illegible]l) and 23 survive to one year old (i.e. 28% survival). Only considering the litters born in 2019 (n = 29 pups), then 26 survived to three months (i.e. 90% survival) and 23 survived to six, nine and twelve months (i.e. 79% survival). There is no discernible difference in pup survival relative to the three, six, nine and twelve month age milestones ($\chi^2_{(3)}$ = 0.03, p = 0.99) and the population had successfully recruited 23 dogs by September 2020, giving a recruitment rate (relative to population size) of 0.47 (indicator 4A, Table 1).

Of the 111 individuals marked and counted during the study, 81 were still alive at the end of the 28 months, resulting in a survival of 73% (indicator 4B, Table 1). When breaking this down to founders versus non-founders, then there is a higher survival rate for founders (25

of 29 individuals, i.e. 86% survival) than non-founders (56 of 82 individuals, i.e. 68% survival; $\chi^2_{(1)}$ = 4.02, p = 0.04).

We observed 30 of the 111 individuals to have died or disappeared from the population (27% of the population missing or dead). The majority of these were pups (90%) and it is likely that these were all mortalities although only one pup carcass was found. Of the three confirmed adult mortalities we documented, one male was killed by lions, a second suspected killed by lions (based on post-mortem and observed signs of trauma on front leg-bones, although inconclusive given the time that had elapsed) and one female was found dead in a den. One male disappeared during the study and we could not confirm if he had died or dispersed. Overall, we found no human-induced mortality on this wild dog population (indicator 9B, Table 1).

Population size

The wild dog population in GNP has grow[illegible]nce their reintroduction in June 2018 (Fig 3). Over the 28 month study period, we identified 111 unique individuals i[illegible]e population (29 founders, 82 non-founders) with population size peaking in June 2020 at 102 individuals (49 adults and yearlings, 53 pups) in four packs and three dispersal groups (Table 2). The wild dog population size in GNP has increased significantly since their reintroduction in June 2018 (adult, yearlings and pup linear model: $F_{(1,2)}$ = 33.50, p = 0.04, R^2 = 0.92; number of packs linear model: $F_{(1,2)}$ = 1.30, p = 0.02, R^2 = 0.97; Fig 3) (indicator 5A, Table 1). By the end of September 2020, there were 81 individuals (48 adults and yearlings, 33 pups) in four packs and three dispersal groups in GNP.

Fig 3. Annual time series of the wild dog population in Gorongosa National Park from one year prior to their reintroduction (in 2017) until 2020. Counts were taken in June of each year

representing the midpoint in the annual breeding season. The bars represent the total number of individuals in the population (primary y-axis) with the solid line representing the number of packs (secondary y-axis).

Table 2. Descriptive patterns of annual population dynamics for the reintroduced wild dog population in Gorongosa National Park between June 2018 and September 2020. AY = adults & yearlings combined, AYP = adults, yearlings & pups combined.

Variable	Mean ± SE	Range
AY count	25.67 ± 11.67	14 – 49
Pup count	27.33 ± 15.32	0 – 53
AYP count	53.00 ± 25.89	14 – 102
Population density (AY/100km^2)	0.69 ± 0.31	0.38 – 1.32
Population density (AYP/100km^2)	1.42 ± 0.70	0.38 – 2.74
Pack size (AY)	10.04 ± 0.63	6.5 – 14
Pack size (AYP)	15.88 ± 0.86	7 – 25.5
Number of packs	2.33 ± 0.88	1 – 4
Pack density (/100km^2)	0.06 ± 0.02	0.03 – 0.11
Number dispersal groups	1.00 ± 1.00	0 – 3

Diet

We observed 103 kills from nine prey species to have been killed by wild dogs. The majority of kills comprised bushbuck (40%) and waterbuck (30%) but wild dogs only positively selected bushbuck while avoiding all other species (Fig. 4, S4 Table) (indicator 6A, Table 1). We also recorded 118 lion kills but with marked differences to those of the 102 wild dog kills regarding species and age class (Fig. 4, S4 Table). For example, lions did not predate

upon bushbuck whereas bushbuck makes up 40% of the wild dogs diet and is their preferred prey (Fig. 4, S4 Table) (indicator 8A, Table 1). Additionally, wild dogs rarely predated on warthog, while warthog comp[illegible]s 58% of observed lion diet (Fig. 4, S4 Table) (indicator 8A, Table 1). Of the total adult waterbuck predated by each species, 81% of those taken by lion were male and 15% female (n = 26) compared to wild dogs that killed 50% male waterbuck and 50% female waterbuck (Fig. 4, S4 Table) (indicator 8A, Table 1). Of the 23 confirmed locations for waterbuck kills made by wild dogs, 10% occurred on the floodplain (versus woodland habitats), compared to 67% of the 27 confirmed waterbuck kills made by lions (S4 Table).

Fig 4. Comparison of Jacob's electivity index values between wild dogs (n = 102 kills) and lions (n = 108 kills) in Gorongosa National Park between June 2018 and September 2020. The order of prey species is based on most preferred (top of figure) to least preferred (bottom of figure) for wild dogs.

Space use

Range expansion

The population expanded their range over two-fold (217% increase) from the first half of the study period (501km^2; 13% of park; June 2018 – July 2019) to the second half (1,089km^2; 29% of park; Aug 2019 – Sep 2020) (S1 Fig). Over the total 28 month study period, wild dogs in Gorongosa used 32% of the park. The total monthly area used by packs in the park has increased over the 28 month study period ($F_{(1,26)} = 75.11$, $p < 0.001$, $R^2 = 0.74$) (indicator 7A, Table 1) and does not appear to have reached an asymptote (Fig 5).

Fig 5. The monthly area size used by the reintroduced wild dogs between June 2018 and September 2020. Area size was delineated by the 95% minimum convex polygon enclosing all GPS fixes for all wild dogs combined.

Territory size and overlap

Wild dogs in GNP formed distinct territories, concentrating mainly in the western part of the park and appeared to avoid high human density areas (Fig 6) (indicator 9A, Table 1). Wild dogs incorporated the release enclosure at some stage in their early release as part of their territories, but all groups eventually established adjacent core ranges excluding the enclosure, with the exception of the first pack released in 2018 (Fig 6). Territory size for the four packs was 356 ± 62 km^2 (mean ± SE) and core territory size was 71 ± 14 km^2 (mean ± SE). Area used for the two dispersal groups was 613 ± 92 km^2 (mean ± SE) and their core areas were 118 ± 12 km^2 (mean ± SE). The mean ratio of core to total territory size was 0.20 ± 0.01 km (mean ± SE, range 0.18 – 0.22) for all six groups of wild dogs in GNP (Fig 6) (indicator 7B, Table 1). Wild dogs in GNP overlapped with conspecifics and thus did not maintain exclusive territories. The mean proportion of overlap between pack territories was 0.21 ± 0.02 km^2 (mean ± SE) while overlap in core territories was 0.07 ± 0.03 km^2 (mean ± SE). The two dispersal groups overlap by 48% in the total territory area and 22% in the core.

Fig 6. The 95% territory area (light grey) and the 50% core territory area (dark grey) for wild dogs in Gorongosa National Park (solid black line) between June 2018 and September 2020 overlayed on the human population density raster for the Gorongosa-Marromeu Landscape. Panels represent different groups of wild dogs: (A) Gorongosa pack, (B) Pwadzi pack, (C) Cheza pack, (D) Mopane pack, (E) Mucodza dispersal group and (F) Xivulo dispersal group.

The diamond symbol is the enclosure release site for the two founder packs (Gorongosa and Pwadzi packs).

Habitat use

Wild dogs used broad vegetation types differently in the park, with significantly higher use of woodland compared to floodplain areas ($\chi^2_{(1)}$ = 2922.6, p < 0.001; Fig 7; S2 Fig). Additionally, wild dogs made significantly more kills in woodland than floodplain areas ($\chi^2_{(1)}$ = 59.84, p < 0.001; S2 Fig). Lions exhibit the same pattern of habitat use, where they used woodland areas more than floodplain areas ($\chi^2_{(1)}$ = 39.37, p < 0.001; Fig 7). However, wild dogs used woodland areas significantly more than lions did ($\chi^2_{(1)}$ = 91.75, p < 0.001; Fig 7) while using floodplain areas significantly less than lions did ($\chi^2_{(1)}$ = 759.3, p < 0.001; Fig 7) (indicator 8B, Table 1). Broad vegetation type affected den site location, with significantly more den sites in woodland (n = 28) than floodplain (n = 1) ($\chi^2_{(1)}$ = 34.78, p < 0.001; S3 Fig). Additionally, all breeding dens were located in core areas for the four packs, except one den for Cheza just outside of core (S3 Fig) and we note interesting den site observations in S1 Appendix.

Fig 7. The difference in woodland and floodplain habitat use by wild dogs and lions in Gorongosa National Park between June 2018 and September 2020. Error bars represent intergroup variation in the proportion of GPS fixes while three stars indicate highly significant differences (i.e. p < 0.001) in habitat use both within and between these two carnivore species.

Lion co-existence

Wild dog territories in GNP overlapped with those of collared lion prides (n = 5), coalitions (n = 4) and dispersers (n = 5) (Fig 8). Overlap between wild dogs and lions was

higher in the total territory areas (56% overlap in the 95% isopleth; Fig 8A) while core territory areas were more exclusive between wild dogs and lions (17% overlap in the 50% isopleths; Fig 8B) (indicator 8A, Table 1).

Fig 8. Territories of wild dogs (solid line) and lions (dotted line) in Gorongosa National Park between June 2018 and September 2020 for the (A) 95% territory areas and (B) the 50% core territory areas. The boundary of GNP is shown as thin black line with the maps overlaid on that of human population density for the Gorongosa-Marromeu landscape.

Discussion

Our study highlights that understanding the successes and challenges of a conservation intervention such as the reintroduction of an endangered species demands rigorous monitoring at all stages of the process [24, 40]. Using a combination of GPS collars, camera traps at den sites, and field observations provided a high-resolution understanding of the dynamics of this recovering species during these first two years. The ongoing studies and the success of reintroduction has also been contingent on expert personnel from GNP routinely collaborating and sharing data with wild dog reintroduction experts from the South African metapopulation (indicator 10B, Table 1). Additionally, local vets and on-the-ground monitoring teams have provided oversight of the recovering population and a wealth of data that has been essential in the identification of challenges and towards evaluating successes/failures (indicator 10A, Table 1).

Overall, the reintroduction of wild dogs to GNP has been highly successful. Of our 16 indicators of reintroduction success, 15 of these were completely achieved while one (annual breeding, indicator 3A) was partially achieved. With the two reintroduced packs having had

dispersal events that resulted in natural pack formations, this is a key determinant for short-term success [40]. Although wild dogs did not breed in the 2018 denning season, they successfully raised pups in both 2019 and 2020. The Gorongosa pack was released in mid-June 2018 after a suggested period of eight weeks of bonding [40, 48], although the alpha female was visibly pregnant. This timing is normally mid-way through the denning season for seasonally breeding wild dog populations [51, 68, 70]. This could explain the unsuccessful 2018 denning season as the released pack may not have had enough time to select a suitable den site, which is essential in ensuring pup production and survival [101]. When considering all the indicators together, it is notable that packs formed both artificially and naturally, individuals had survival rates equal to the highest ever documented in the wild (Selous Game Reserve – Creel and Creel (68); Hluhluwe-iMfolozi Park – Marneweck (50)), breeding occurred in all packs, pups were recruited into the population, no human-induced mortalities occurred and wild dogs effectively avoided lions through territory establishment and habitat use. Consequently, we conclude that over this short-term period since the first reintroduction (i.e. 28 months), this population of wild dogs has been successfully reintroduced and is still within the expansion phase (continued increase in space use, variable population size etc.). This highlights how quickly wild dogs that were naïve to GNP adapted to the local conditions [24]. Density-dependent regulation of vital rates and the emergent population size does not appear to be influencing this population yet, although anecdotally, competition at dens and between pups seemed to have limited survival of the 2020 litters.

Wild dogs in GNP displayed two notable patterns that we suggest aided in rapid population expansion. Firstly, we observed multiple maternities per pack more often than alpha-only litters. Normally, breeding is monopolized by an alpha pair whereas shared maternity is very rare [50, 102, 103]. While we acknowledge this is a small sample size of multiple maternities (five females in two packs, across two years), without subordinate females

breeding this would have resulted in 19 fewer pups born which could have pack and population size effects. We suggest that the multiple maternities per pack was a key factor in the rapid population growth at these early stages. Secondly, the reintroduction of the second pack (i.e. Pwadzi pack) appeared to stimulate dispersal from the Gorongosa pack which in-turn stimulated dispersal within the Pwadzi pack. This resulted in the formation of a fourth pack in GNP. In small fenced areas like the metapopulation in South Africa, single packs and their dispersing cohorts struggle to find mates (mate-finding allee effect) which can constrain population growth [40, 53, 73]. Thus, by introducing a second pack, this resulted in three quick dispersal events (Fig 2), which more closely simulated natural population dynamics. This mimicry of natural dynamics is a key factor in the intensive management of a metapopulation that uses translocation and reintroduction to mimic natural dynamics [21, 22]. We suggest that the long-term objective of the project is to phase out intensive interventions (such as reintroduction) with ideally all dynamics being natural and the population being self-sustaining [40, 41].

GNP can sustain more wild dogs as current packs and dispersal groups only used 32% of the park. We estimate that based on the possibility of wild dogs using 100% of the park, that GNP could sustain a total of 12 packs and six dispersal groups. This assumes the same level of resource and competition distribution and territory size and use in the currently vacant 68% of the park. Analytical examination of multiple carrying capacity models should be used to evaluate our suggestion. For example, the Hayward, Obrien (81) carrying capacity model and the EWT feasibility criteria assessment could provide baselines from which to build biologically relevant carrying capacity models that include aspects of observed habitat use, space use and prey selection. Regardless of carrying capacity figures produced, GNP will need to decide on whether to fill vacant habitat with wild dogs through either, (1) natural colonization of the current reintroduced wild dogs, (2) reintroducing more wild dogs or (3) a

combination of both. Next-steps will be to develop population projections for GNP that account for uncertainties relating to demographic stochasticity (i.e. chance fates for individuals) and environmental stochasticity (i.e. large environmental fluctuations) to guide GNP management on what the optimal population size is that warrants no further reintroduction effort. This, in combination with carrying capacity models are key next steps to help guide medium-term objective for the reintroduced wild dog population in GNP to occupy as much suitable habitat as possible. If further reintroductions in GNP are to occur then focus should be on the eastern and northern parts of GNP because: (1) these are further from high human population density areas, (2) currently vacant areas with regards to wild dogs and (3) occupation of these areas could stimulate range-expansion eastwards into the greater Gorongosa-Marromeu landscape. We also suggest a need to monitor genetic diversity [41] and if further reintroductions are deemed appropriate then sourcing individuals from geographically closer to extant range is viable [25, 28].

The reintroduction of wild dogs to GNP is the first successful reintroduction of wild dogs into an open system documented in the literature [24]. All prior published wild dog reintroduction efforts have occurred within the small fenced reserves in the South African metapopulation [21, 22]. While large portions of GNP are vacant for wild dog expansion and/or reintroduction, there exists a great opportunity for expansion of this species into the larger Gorongosa-Marromeu landscape. Despite wild dogs being documented historically in more eastern reaches of Gorongosa-Marromeu (Coutada 12, Fig 1 area D) in past years (J. Andre, unpublished report), recent camera-trap surveys and field sightings by GNP have indicated only a single remnant pack of five individuals (1 female and 4 males) remain there. This pack potentially represents the last extant group in that area (P. Bouley, unpublished data) and is therefore highly endangered. In 2018, GNP took over management of Coutada 12 with the goal of helping to secure the greater Gorongosa-Marromeu landscape, thus expanding

reintroduction and population expansion potential into a much larger area that could connect the reintroduced population in GNP with the extant pack in Coutada 12. This large-scale connectivity work is critical to the species in this region. Our long-term goal to connect populations across the Gorongosa-Marromeu landscape, and to significantly increase the area and population size of wild dogs in the region, is in stark contrast against the global paradigm of declining wild dog populations and ranges across Africa [16]. With multiple recoverable and open sites in the region [25], this project could also represent a framework for success in future conservation efforts for wild dogs, especially with regards to reintroductions in open systems. With an ever increasing strain on natural areas and resources, reintroductions and active management interventions remain a viable tools for the species' conservation [41]. Our workflow of a phased reintroduction, establishing and monitoring indicators of success could thus provide an important framework for future reintroductions of wild dogs that can also be broadly applied to a range of other similar carnivore species.

Acknowledgements

We want to thank management of Gorongosa National Park for approving this reintroduction and the subsequent research. We also thank Ezemvelo KZN Wildlife, Khamab Kalahari Game Reserve and the North West Provincial Government in South Africa for donating the founder wild dogs to the project. We also thank WildlifeACT in South Africa for logistical, technical and veterinary support in this project and the Bateleurs for providing the charter plane and pilots to fly the wild dogs in both reintroduction phases. We thank the various GNP field rangers, guides and tourists for submitting valuable wild dog sightings to the project.

References

1. Beschta RL, Ripple WJ. Large predators and trophic cascades in terrestrial ecosystems of the western United States. Biological Conservation. 2009; 142: 2401-14.

2. Ripple WJ, Estes JA, Beschta RL, Wilmers CC, Ritchie EG, Hebblewhite M, et al. Status and ecological effects of the world’s largest carnivores. Science. 2014; 343: 1241484.

3. Schmitz OJ, Hawlena D, Trussell GC. Predator control of ecosystem nutrient dynamics. Ecology Letters. 2010; 13: 1199-209.

4. Ritchie EG, Elmhagen B, Glen AS, Letnic M, Ludwig G, McDonald RA. Ecosystem restoration with teeth: what role for predators? Trends in Ecology & Evolution. 2012; 27: 265-71.

5. Terborgh J. The big things that run the world-a sequel to EO Wilson. Conservation Biology. 1988; 2: 402-3.

6. Terborgh J, Lopez L, Tello J, Yu D, Bruni AR. Transitory states in relaxing land bridge islands. In: Laurance WF, Bierregaard ROJ, editors. Tropical Forest Remnants: Ecology, Management, and Conservation of Fragmented Communities: University of Chicago Press, Chicago; 1997.

7. Estes JA, Tinker MT, Williams TM, Doak DF. Killer whale predation on sea otters linking oceanic and nearshore ecosystems. Science. 1998; 282: 473-6.

8. Schoener TW, Spiller DA. Indirect effects in an experimentally staged invasion by a major predator. The American Naturalist. 1999; 153: 347-58.

9. Laundré JW, Hernández L, Altendorf KB. Wolves, elk, and bison: reestablishing the" landscape of fear" in Yellowstone National Park, USA. Canadian Journal of Zoology. 2001; 79: 1401-9.

10. Laundré JW, Hernández L, Medina PL, Campanella A, López- Portillo J, González-Romero A, et al. The landscape of fear: The missing link to understand top- down and bottom-up controls of prey abundance. Ecology. 2014; 95: 1141-52.

11. Stolton S, Dudley N. The New Lion Economy. Unlocking the value of lions and their landscapes. Bristol, UK: 2019.

12. Chambers CM, Whitehead JC. A contingent valuation estimate of the benefits of wolves in Minnesota. Environmental and Resource Economics. 2003; 26: 249-67.

13. Macdonald C, Gallagher AJ, Barnett A, Brunnschweiler J, Shiffman DS, Hammerschlag N. Conservation potential of apex predator tourism. Biological Conservation. 2017; 215: 132-41.

14. Richardson L, Loomis J. The total economic value of threatened, endangered and rare species: an updated meta-analysis. Ecological Economics. 2009; 68: 1535-48.

15. Estes JA, Terborgh J, Brashares JS, Power ME, Berger J, Bond WJ, et al. Trophic downgrading of planet Earth. Science. 2011; 333: 301-6.

16. Wolf C, Ripple WJ. Range contractions of the world's large carnivores. Royal Society Open Science. 2017; 4: 170052.

17. Wolf C, Ripple WJ. Prey depletion as a threat to the world's large carnivores. Royal Society Open Science. 2016; 3: 160252.

18. Ceballos G, Ehrlich PR. Mammal population losses and the extinction crisis. Science. 2002; 296: 904-7.

19. Morrison JC, Sechrest W, Dinerstein E, Wilcove DS, Lamoreux JF. Persistence of large mammal faunas as indicators of global human impacts. Journal of Mammalogy. 2007; 88: 1363-80.

20. Macdonald DW, Sillero-Zubiri C, editors. Large carnivores and conflict: lion conservation in context. Lion conservation research Workshop; 2002.

21. Gusset M, Ryan SJ, Hofmeyr M, Van Dyk G, Davies-Mostert HT, Graf JA, et al. Efforts going to the dogs? Evaluating attempts to re-introduce endangered wild dogs in South Africa. Journal of Applied Ecology. 2008; 45: 100-8. doi: 10.1111/j.1365-2664.2007.01357.x.

22. Davies-Mostert HT, Mills MGL, Macdonald DW. A critical assessment of South Africa's managed metapopulation recovery strategy for African wild dogs. In: Hayward MW, Somers MJ, editors. Reintroduction of top-order predators. Oxford, UK: Blackwell Publishing; 2009. pp. 10-42.

23. Ausband DE, Foresman KR. Swift fox reintroductions on the Blackfeet Indian Reservation, Montana, USA. Biological conservation. 2007; 136: 423-30.

24. Moehrenschlager A, Somers MJ. Canid reintroductions and metapopulation management. 2004.

25. IUCN/SSC. Review of the Regional Conservation Strategy for the Cheetah and African Wild Dogs in Southern Africa. IUCN/SSC Gland, Switzerland: IUCN/SSC Gland, Switzerland and Range Wide Conservation Program for Cheetah and African Wild Dogs, 2015.

26. Woodroffe R, Sillero-Zubiri C. *Lycaon pictus*. Gland, Switzerland: IUCN, 2012.

27. Chavez DE, Gronau I, Hains T, Kliver S, Koepfli K-P, Wayne RK. Comparative genomics provides new insights into the remarkable adaptations of the African wild dog (*Lycaon pictus*). Scientific reports. 2019; 9: 1-14.

28. Marsden CD, Woodroffe R, Mills MGL, McNutt JW, Creel S, Groom R, et al. Spatial and temporal patterns of neutral and adaptive genetic variation in the endangered African wild dog (*Lycaon pictus*). Molecular Ecology. 2012; 21: 1379-93.

29. Woodroffe R, Ginsberg JR. Edge effects and the extinction of populations inside protected areas. Science. 1998; 280: 2126-8.

30. Becker M, McRobb R, Watson F, Droge E, Kanyembo B, Murdoch J, et al. Evaluating wire-snare poaching trends and the impacts of by-catch on elephants and large carnivores. Biological conservation. 2013; 158: 26-36.

31. Wallach AD, Izhaki I, Toms JD, Ripple WJ, Shanas U. What is an apex predator? Oikos. 2015; 124: 1453–61.

32. le Roux E, Marneweck DG, Clinning G, Druce DJ, Kerley GIH, Cromsigt JPGM. Top–down limits on prey populations may be more severe in larger prey species, despite having fewer predators. Ecography. 2019; 42: 1-9. doi: 10.1111/ecog.03791.

33. Pole A, Gordon IJ, Gorman ML, MacAskill M. Prey selection by African wild dogs (Lycaon pictus) in southern Zimbabwe. Journal of Zoology. 2004; 262: 207-15. doi: 10.1017/s0952836903004576.

34. Stalmans ME, Massad TJ, Peel MJS, Tarnita CE, Pringle RM. War-induced collapse and asymmetric recovery of large-mammal populations in Gorongosa National Park, Mozambique. PloS one. 2019; 14: e0212864.

35. Tinley KL. Framework of the Gorongosa ecosystem, Mozambique. Sc.(Ecology). University of Pretoria. 1977. Pretoria South Africa.

36. Daskin JH, Stalmans M, Pringle RM. Ecological legacies of civil war: 35- year increase in savanna tree cover following wholesale large- mammal declines. Journal of Ecology. 2016; 104: 79-89.

37. Stalmans M, Peel M, Massad T. Aerial wildlife count of the Parque Nacional da Gorongosa, Mozambique, October 2014: Approach, results and discussion. 2014.

38. Pringle RM. Upgrading protected areas to conserve wild biodiversity. Nature. 2017; 546: 91-9.

39. Bouley P, Poulos M, Branco R, Carter NH. Post-war recovery of the African lion in response to large-scale ecosystem restoration. Biological Conservation. 2018; 227: 233-42.

40. Gusset M. A framework for evaluating reintroduction success in carnivores: lessons from African wild dogs. In: Hayward MW, Somers MJ, editors. Reintroduction of top-order predators. Oxford, UK: Blackwell Publishing; 2009. pp. 307-20.

41. IUCN/SSC. Guidelines for Reintroductions and Other Conservation Translocations, Version 1.0. Gland, Switzerland: IUCN Species Survival Commission, 2013.

42. IUCN/SSC. GLOBAL RE-INTRODUCTION PERSPECTIVES: re-introduction case-studies from around the globe. Abu Dhabi, UAE.: 2008.

43. IUCN/SSC. GLOBAL RE-INTRODUCTION PERSPECTIVES: Additional case-studies from around the globe. Abu Dhabi, UAE: 2010.

44. IUCN/SSC. Global Re-introduction Perspectives: 2011: More case studies from around the globe. Gland, Switzerland: 2011.

45. IUCN/SSC. Global Re-introduction Perspectives: 2013. Further case studies from around the globe. . Gland, Switzerland and Abu Dhabi, UAE: 2013.

46. IUCN/SSC. Global Re-introduction Perspectives: 2016. Case-studies from around the globe. Gland, Switzerland & Abu Dhabi, UAE: 2016.

47. IUCN/SSC. Global Reintroduction Perspectives: 2018. Case studies from around the globe. Gland, Switzerland & Abu Dhabi, UAE.: 2018.

48. Marneweck C, Becker PA, Beverley G, Davies- Mostert HT, du Plessis C, Forssman K, et al. Factors affecting the success of artificial pack formation in an endangered, social carnivore: the African wild dog. Animal Conservation. 2019; 22: 493-502. doi: 10.1111/acv.12490.

49. Marneweck CJ, Marchal AFJ, Marneweck DG, Beverley G, Davies-Mostert HT, Parker DM. A novel technique for artificial pack formation in African wild dogs using odour familiarity. African Journal of Wildlife Research. 2019; 49: 116-20.

50. Marneweck DG. The ecology of African wild dogs (*Lycaon pictus*), Temminck 1820, in Hluhluwe-iMfolozi Park, South Africa. PhD Thesis. University of Pretoria. 2020. Pretoria, South Africa.

51. Buettner UK, Davies-Mostert HT, du Toit JT, Mills MGL. Factors affecting juvenile survival in African wild dogs (*Lycaon pictus*) in Kruger National Park, South Africa. Journal of Zoology. 2007; 272: 10-9.

52. Woodroffe R, Davies-Mostert H, Ginsberg J, Graf J, Leigh K, McCreery K, et al. Rates and causes of mortality in endangered African wild dogs *Lycaon pictus*: lessons for management and monitoring. Oryx. 2007; 41: 215-23. doi: 10.1017/s0030605307001809.

53. Davies-Mostert HT, Mills MGL, Macdonald DW. The demography and dynamics of an expanding, managed African wild dog metapopulation. African Journal of Wildlife Research. 2015; 45: 258-73.

54. Behr DM, McNutt JW, Ozgul A, Cozzi G. When to stay and when to leave? Proximate causes of dispersal in an endangered social carnivore. Journal of Animal Ecology. 2020. doi: 10.1111/1365- 2656.13300.

55. Jenkins E, Silva-Opps M, Opps SB, Perrin MR. Home range and habitat selection of a reintroduced African wild dog (*Lycaon pictus*) pack in a small South African game reserve. African Journal of Wildlife Research. 2015; 45: 233-46.

56. Woodroffe R, O'Neill HMK, Rabaiotti D. Within- and between-group dynamics in an obligate cooperative breeder. Journal of Animal Ecology. 2019; 00: 1-11. doi: 10.1111/1365-2656.13102.

57. Marneweck C, Marneweck DG, van Schalkwyk OL, Beverley G, Davies-Mostert HT, Parker DM. Spatial partitioning by a subordinate carnivore is mediated by conspecific overlap. Oecologia. 2019; 191: 531-40.

58. Mills MGL, Gorman ML. Factors Affecting the Density and Distribution of Wild Dogs in the Kruger National Park. Conservation Biology. 1997; 11: 1397-406.

59. Darnell AM, Graf JA, Somers MJ, Slotow R, Szykman Gunther M. Space use of African wild dogs in relation to other large carnivores. PLoS One. 2014; 9: e98846. doi: 10.1371/journal.pone.0098846.

60. Creel S, Merkle J, Mweetwa T, Becker MS, Mwape H, Simpamba T, et al. Hidden Markov Models reveal a clear human footprint on the movements of highly mobile African wild dogs. Scientific reports. 2020; 10: 17908-.

61. Hayward MW, O'Brien J, Hofmeyr M, Kerley GIH. Prey preferences of the African wild dog *Lycaon pictus* (Canidae: Carnivora): ecological requirements for conservation. Journal of Mammology. 2006; 87: 1122-31.

62. Somers MJ, Becker PA, Druce DJ, Graf JA, Szykman Gunther M, Marneweck DG, et al. Reassembly of the large predator guild into Hluhluwe-iMfolozi Park. In: Cromsigt JPGM, Archibald S, Owen-Smith N, editors. Conserving Africa's Mega-Diversity in the Anthropocene The Hluhluwe-iMfolozi Park Story. Ecology, Biodiversity and Conservation. Cambridge: Cambridge University Press; 2017. pp. 286-310.

63. Vogel JT, Somers MJ, Venter JA. Niche overlap and dietary resource partitioning in an African large carnivore guild. Journal of Zoology. 2019. doi: 10.1111/jzo.12706.

64. Cukor J, Havránek F, Linda R, Bukovjan K, Painter MS, Hart V. First findings of brown hare (*Lepus europaeus*) reintroduction in relation to seasonal impact. PloS one. 2018; 13: e0205078.

65. Mignet F, Gendre T, Reudet D, Malgoire F, Cheylan M, Besnard A. Short-term evaluation of the success of a reintroduction program of the European Pond Turtle: the contribution of space-use modeling. Chelonian Conservation and Biology. 2014; 13: 72-80.

66. Moehrenschlager A, Macdonald DW. Movement and survival parameters of translocated and resident swift foxes *Vulpes velox*. Animal conservation. 2003; 6: 199-206.

67. McCreery EK, Robbins RL. Proximate Explanations for Failed Pack Formation in *Lycaon pictus*. Behaviour. 2001; 138: 1467-79.

68. Creel S, Creel NM. The African Wild Dog: Behavior, Ecology and Evolution. Krebs JR, Clutton-Brock T, editors. Princeton, NJ, USA: Princeton University Press; 2002. 341 p.

69. Creel S, Mills MGL, McNutt JW. African wild dogs: Demography and population dynamics of African wild dogs in three critical populations. In: Macdonald DW, Sillero-Zubiri C, editors. The Biology and Conservation of Wild Canids. New York: Oxford University Press; 2004. pp. 337-50.

70. Marneweck DG, Druce DJ, Somers MJ. Food, family and female age affect reproduction and pup survival of African wild dogs. Behavioral Ecology and Sociobiology. 2019; 73: 1-15. doi: 10.1007/s00265-019-2676-x.

71. Woodroffe R. Ranging behaviour of African wild dog packs in a human-dominated landscape. Journal of Zoology. 2011; 283: 88-97. doi: 10.1111/j.1469-7998.2010.00747.x.

72. Parker MN. Territoriality and scent marking behavior of African wild dogs in northern Botswana. PhD Thesis. University of Montana. 2010. Missoula, Montana , United States of America.

73. Somers MJ, Graf JA, Szykman M, Slotow R, Gusset M. Dynamics of a small re-introduced population of wild dogs over 25 years: Allee effects and the implications of sociality for endangered species' recovery. Oecologia. 2008; 158: 239-47.

74. Sarrazin F, Barbault R. Reintroduction: challenges and lessons for basic ecology. Trends in ecology & evolution. 1996; 11: 474-8.

75. Trettin CC, Stringer CE, Zarnoch SJ. Composition, biomass and structure of mangroves within the Zambezi River Delta. Wetlands ecology and management. 2016; 24: 173-86.

76. Stalmans M, Beilfuss R. Landscapes of the Gorongosa National Park. Gorongosa National Park, Mozambique: 2008.

77. Riggio J, Jacobson A, Dollar L, Bauer H, Becker M, Dickman A, et al. The size of savannah Africa: a lion's (*Panthera leo*) view. Biodiversity and Conservation. 2013; 22: 17-35.

78. Fusari A, Cumbi R, Chardonnet P, Begg C. Conservation Strategy and Action Plan for the African Lion (*Panthera leo leo*) in Mozambique. 2010.

79. Daskin JH, Pringle RM. Warfare and wildlife declines in Africa's protected areas. Nature. 2018; 553: 328-32.

80. Nicholson SK, Marneweck DG, Lindsey PA, Marnewick K, Davies-Mostert HT. A 20-year review of the status and distribution of African wild dogs (*Lycaon pictus*) in South Africa. African Journal of Wildlife Research. 2020; 50: 8-19. doi: 10.3957/056.050.0008.

81. Hayward M, Obrien J, Kerley G. Carrying capacity of large African predators: Predictions and tests. Biological Conservation. 2007; 139: 219-29. doi: 10.1016/j.biocon.2007.06.018.

82. Tensen L, van Vuuren BJ, du Plessis C, Marneweck DG. African wild dogs: Genetic viability of translocated populations across South Africa. Biological Conservation. 2019; 234: 131-9.

83. Graf JA, Gusset M, Reid C, van Rensburg SJ, Slotow R, Somers MJ. Evolutionary ecology meets wildlife management: artificial group augmentation in the re-introduction of endangered African wild dogs (Lycaon pictus). Animal Conservation. 2006; 9: 398-403. doi: 10.1111/j.1469-1795.2006.00048.x.

84. Potgieter KR, O'Riain MJ, Davies-Mostert HT. Behavioural cues can be used to predict the outcome of artificial pack formation in African wild dogs (*Lycaon pictus*). South African Journal of Wildlife Research. 2015; 45: 215-22. doi: 10.3957/056.045.0215.

85. McNutt JW, Silk JB. Pup production, sex ratios, and survivorship in African wild dogs, *Lycaon pictus*. Behavioral Ecology and Sociobiology. 2008; 62: 1061–7.

86. Woodroffe R. Demography of a recovering African wild dog (*Lycaon pictus*) population. Journal of Mammalogy. 2011; 92: 305-15.

87. Stalmans M, Peel M, Gonçalves D. Aerial wildlife count of the Parque Nacional da Gorongosa, Mozambique. 2018.

88. Jacobs J. Quantitative measurement of food selection. Oecologia. 1974; 14: 413-7.

89. Creel S, Creel NM. Limitation of African Wild Dogs by Competition with Larger Carnivores. Conservation Biology. 1996; 10: 526-38.

90. Creel S, Creel NM. Six ecological factors that may limit African wild dogs, *Lycaon pictus*. Animal Conservation. 1998; 1: 1-9.

91. Swanson A, Caro T, Davies-Mostert H, Mills MGL, Macdonald DW, Borner M, et al. Cheetahs and wild dogs show contrasting patterns of suppression by lions. Journal of Animal Ecology. 2014; 83: 1418-27. doi: 10.1111/1365-2656.12231.

92. Estes RD, Goddard J. Prey Selection and Hunting Behavior of the African Wild Dog. The Journal of Wildlife Management. 1967; 31: 52-70.

93. Dröge E, Creel S, Becker M, M'soka J. Spatial and temporal avoidance of risk within a large carnivore guild. Ecology and Evolution. 2017; 7: 189-99. doi: 10.1002/ece3.2616.

94. Silverman BW. Density estimation for statistics and data analysis. London, UK: Chapman and Hall; 1986.

95. Calenge C. Home Range Estimation in R: the adehabitatHR Package. R package version 1952019.

96. Nemtzov SC. Intraspecific variation in home range exclusivity by female green razorfish, Xyrichtys splendens (family Labridae), in different habitats. Environ Biol Fishes. 1997; 50: 371-81. doi: 10.1023/a:1007319622028.

97. Macdonald DW, Ball FG, Hough NG. The evaluation of home range size and configuration using radiotracking data. In: Amlaner CJ, Macdonald DW, editors. A Handbook on biotelemetry and radiotracking. Oxford, UK: Pergamon Press; 1980. pp. 405-24.

98. Sikes RS. 2016 Guidelines of the American Society of Mammalogists for the use of wild mammals in research and education. Journal of mammalogy. 2016; 97: 663-88.

99. RStudio Team. RStudio: Integrated Development for R. 0.99.879 ed. Boston, MA: RStudio Inc.; 2015.

100. R Core Team. R: A language and environment for statistical computing. R Foundation for Statistical Computing. 2.5.1 ed. Vienna, Austria2018.

101. Davies AB, Marneweck DG, Druce DJ, Asner GP. Den site selection, pack composition, and reproductive success in endangered African wild dogs. Behavioral Ecology. 2016; 27: 1869-79.

102. Malcolm JR, Marten K. Natural selection and the communal rearing of pups in African wild dogs (*Lycaon pictus*). Behavioral Ecology and Sociobiology. 1982; 10: 1-13.

103. Creel S, Creel NM, Mills MGL, Monfort SL. Rank and reproduction in cooperatively breeding African wild dogs: Behavioral and endocrine correlates. Behavioral Ecology. 1997; 8: 298-306.

Supporting information captions

S1 Table. Wild dog and lion collars. The number of fixes per wild dog and lion group used in the spatial analyses. The collar coverage dates represent the full time period from group release from enclosure/pack formation except for Cheza pack that formed in March 2019 (Fig 2) but was only GPS collared in August 2019. For the lion data, we only included data from the date of the release of the first pack of wild dogs on 15 June 2018.

S2 Table. Birth details. Pregnancies and confirmed litters for eight female wild dogs in Gorongosa National Park between June 2018 and September 2020.

S3 Table. Group association dynamics. Details of events that resulted in individual founder wild dogs changing their group association in Gorongosa National Park from the time of the first reintroduction in June 2018 until September 2020. AM = adult male, YF = yearling female and AF = adult female.

S4 Table. Wild dog and lion kills. Observed number and proportion of total kills made by wild dogs and lions in Gorongosa National Park between June 2018 and September 2020. Jacob's index of selection is also shown where positive values represent preferred while negative values represent avoided.

S1 Fig. Wild dog range expansion. Total areas used by reintroduced wild dogs in Gorongosa National Park during the first 14 months of the study (June 2018 – July 2019, dashed black line) compared to the second 14 months of the study (Aug 2019 – Sep 2020, solid black line). The location of the release enclosure (diamond), Lake Urema (blue), and broad vegetation categories (woodland – grey; floodplain – green) are also shown. The total areas represent the merged 95% kernel UD isopleths from each pack per half of the study period.

S2 Fig. Kills and wild dog GPS fixes. The location of (A) kills for wild dogs (black dots, n = 72) and kills for lions (red dots, n = 89) and (B) wild dog GPS fixes (n = 2,985) in Gorongosa National Park between June 2018 and September 2020, overlaid onto vegetation type (woodland – grey; floodplain – green), also showing Lake Urema (blue).

S3 Fig. Pack den sites. Den sites (black diamonds) used by packs of wild dogs in Gorongosa National encompassing the 2018, 2019 and 2020 denning seasons overlaid onto the broad vegetation categories (woodland – grey; floodplain – green) and showing Lake Urema (blue). Panels represent different packs, specifically: (A) Gorongosa pack, (B) Pwadzi pack, (C) Cheza pack and (D) Mopane pack with pack specific territory areas shown as solid black lines, representing the 95% territory area (outer black lines) and the 50% core territory area (inner black lines).

S1 Appendix. Den observations. Interesting observations at wild dog den sites in 2019 and 2020 in Gorongosa National Park.

Figure1 Click here to access/download;Figure;Fig1.tif

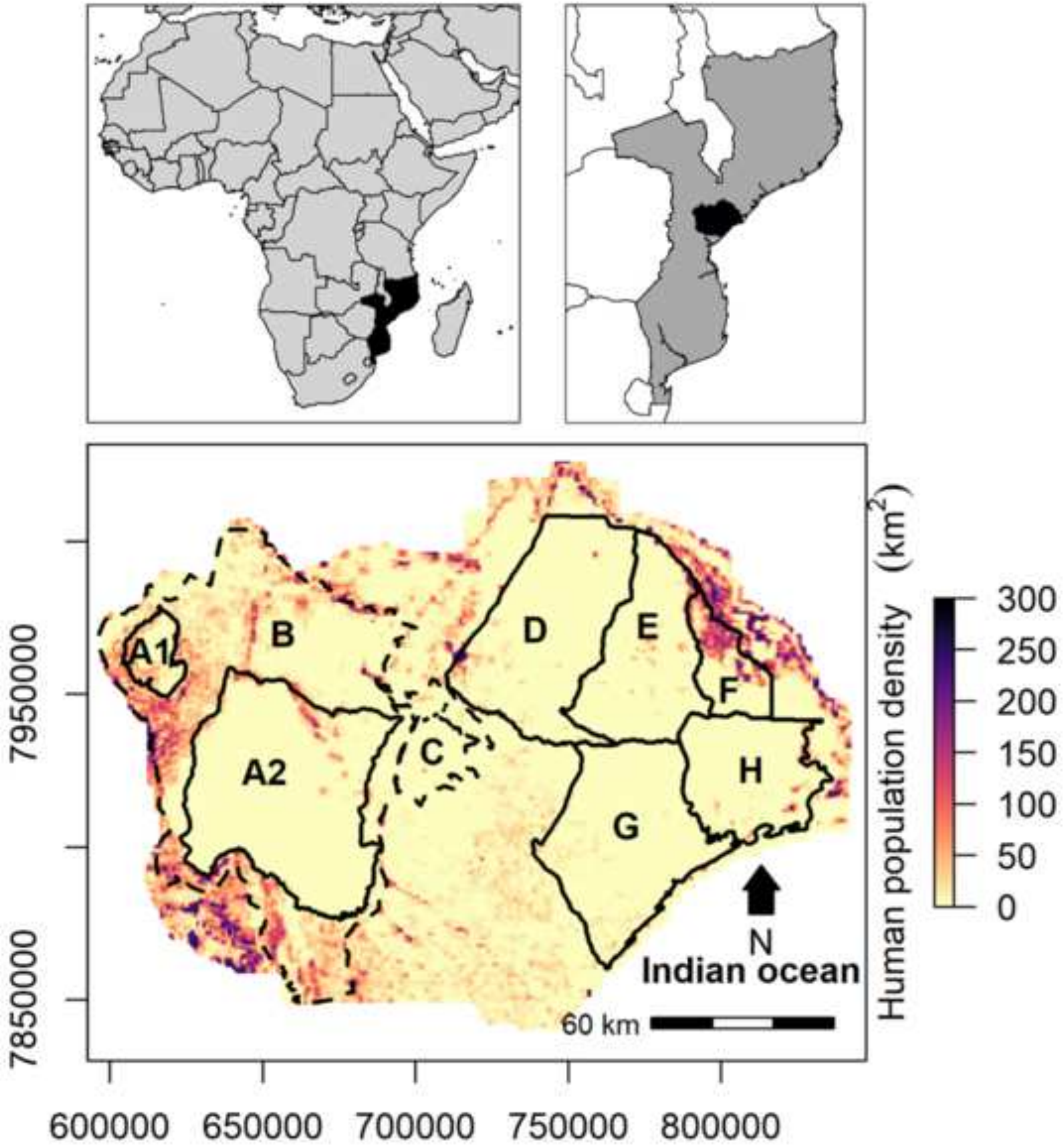

Figure2

Click here to access/download;Figure;Fig2.tif

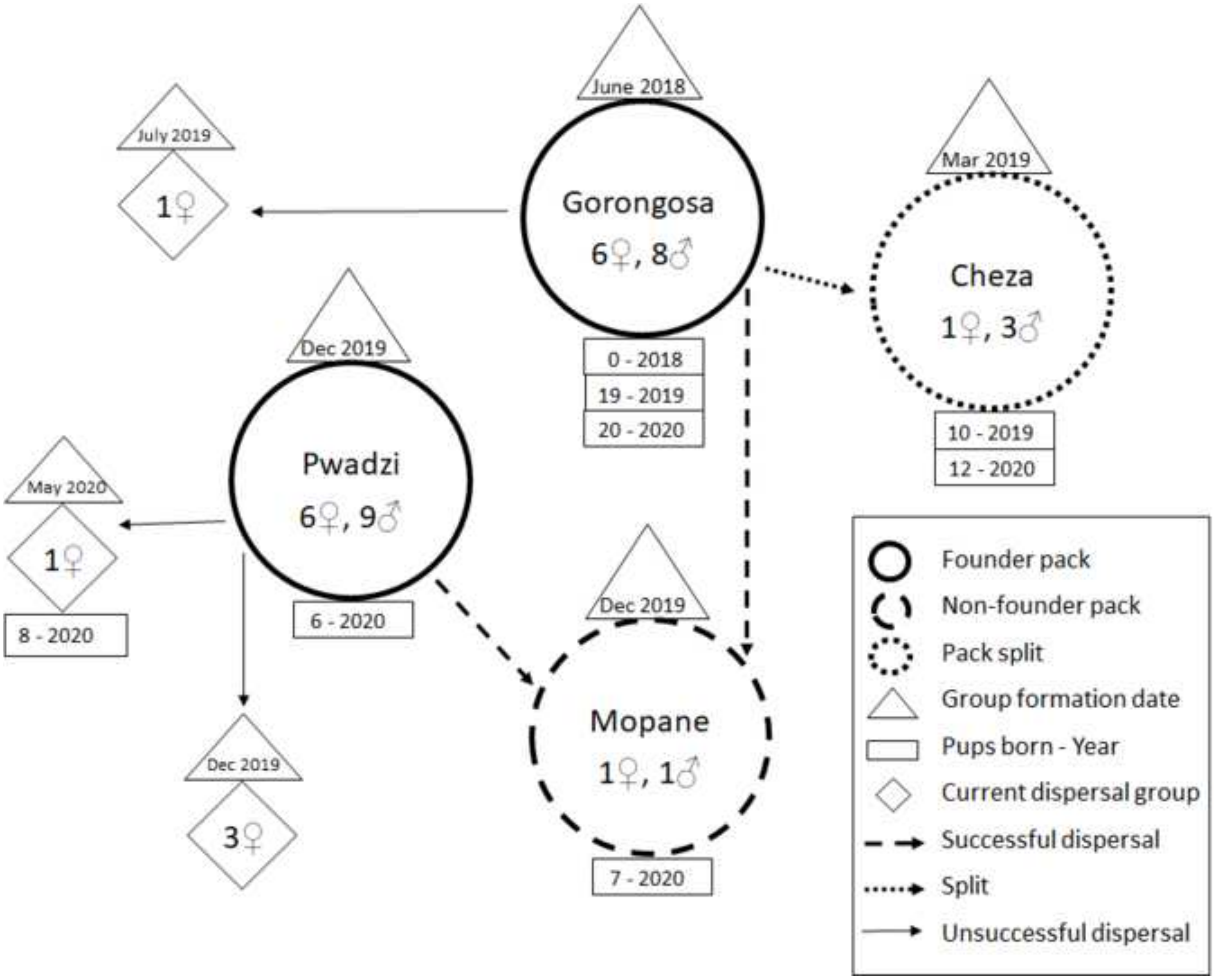

Figure3

Click here to access/download;Figure;Fig3.tif

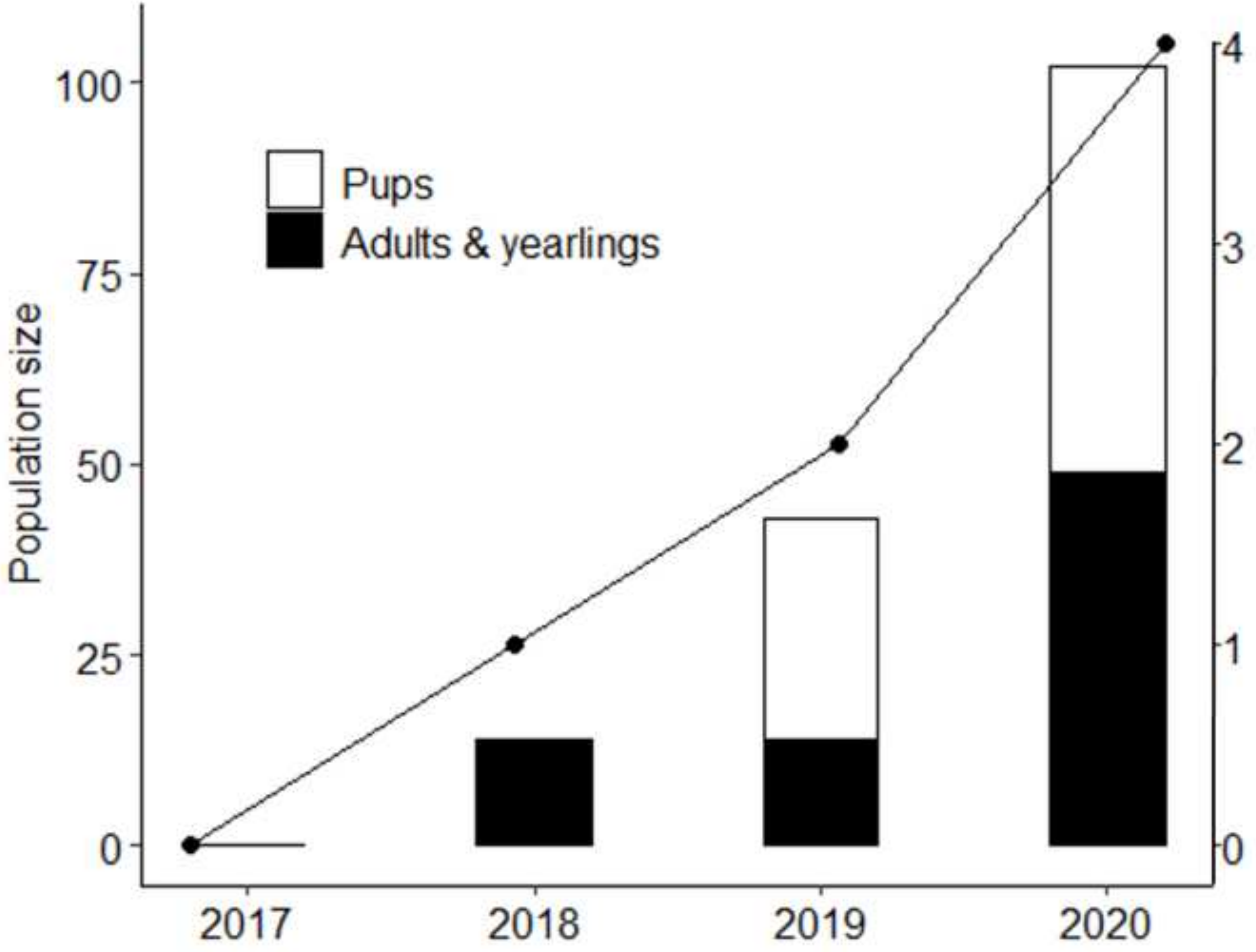

 Click here to access/download;Figure;Fig4.tif

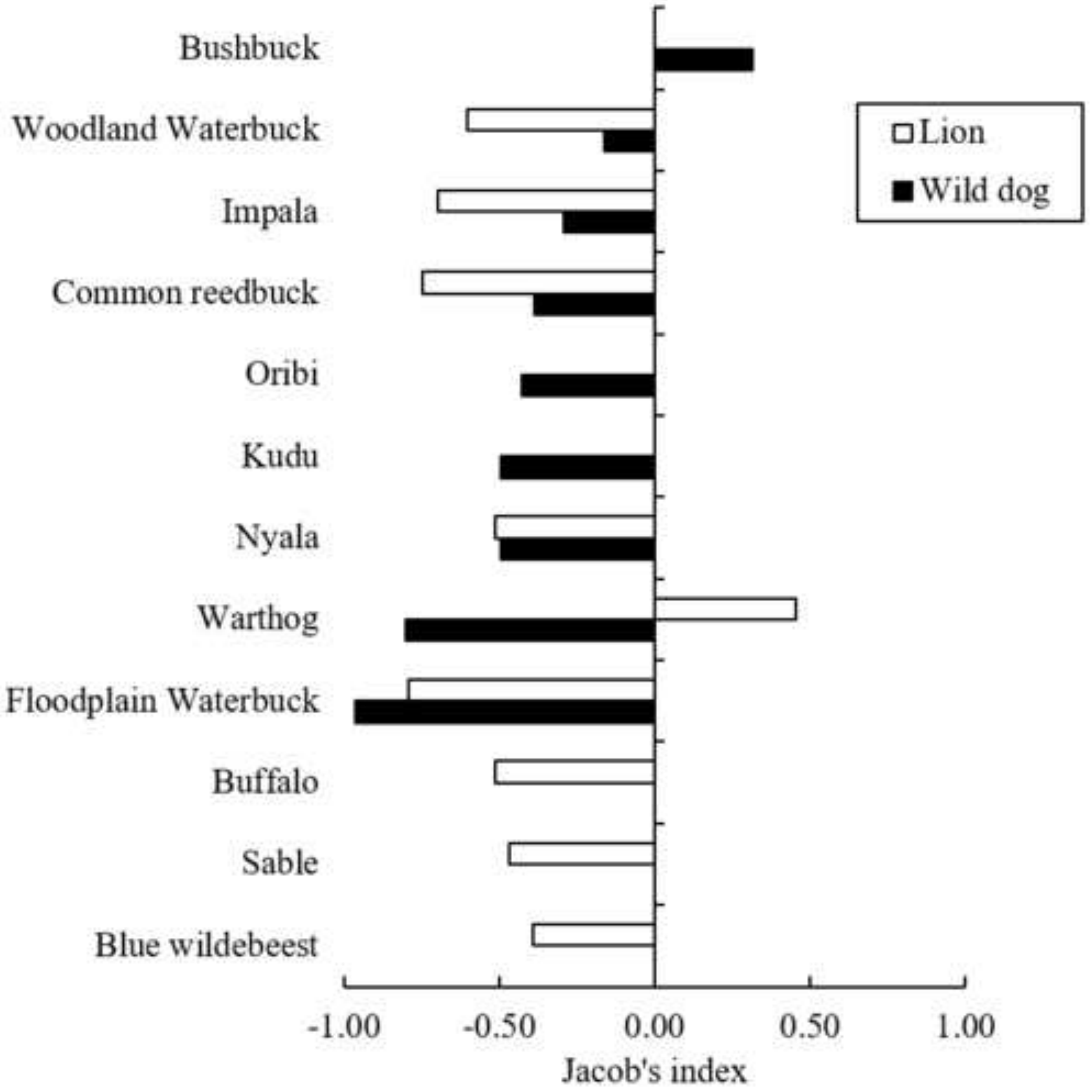

Figure5

Click here to access/download;Figure;Fig5.tiff

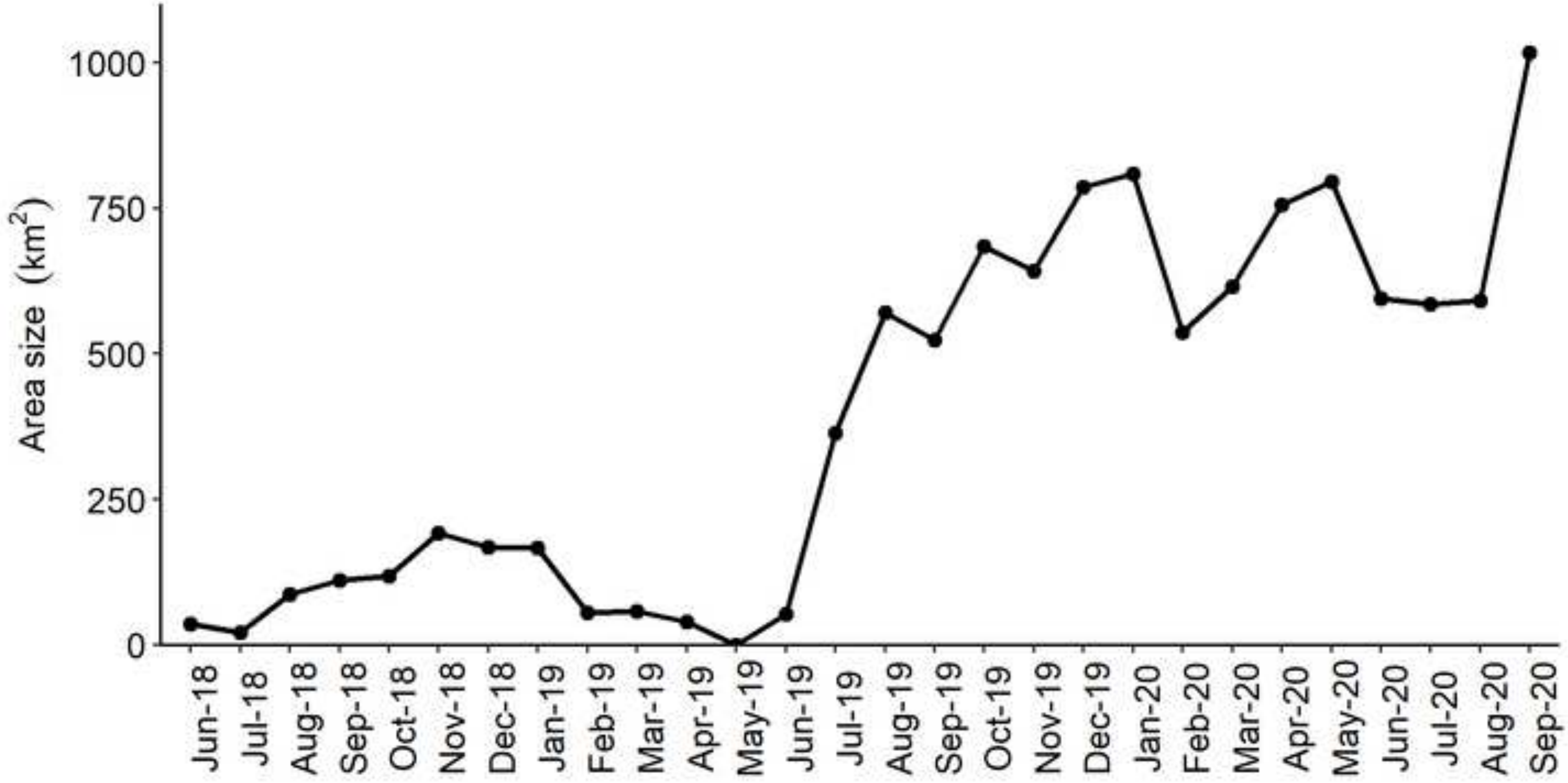

Figure6

Click here to access/download;Figure;Fig6.tiff

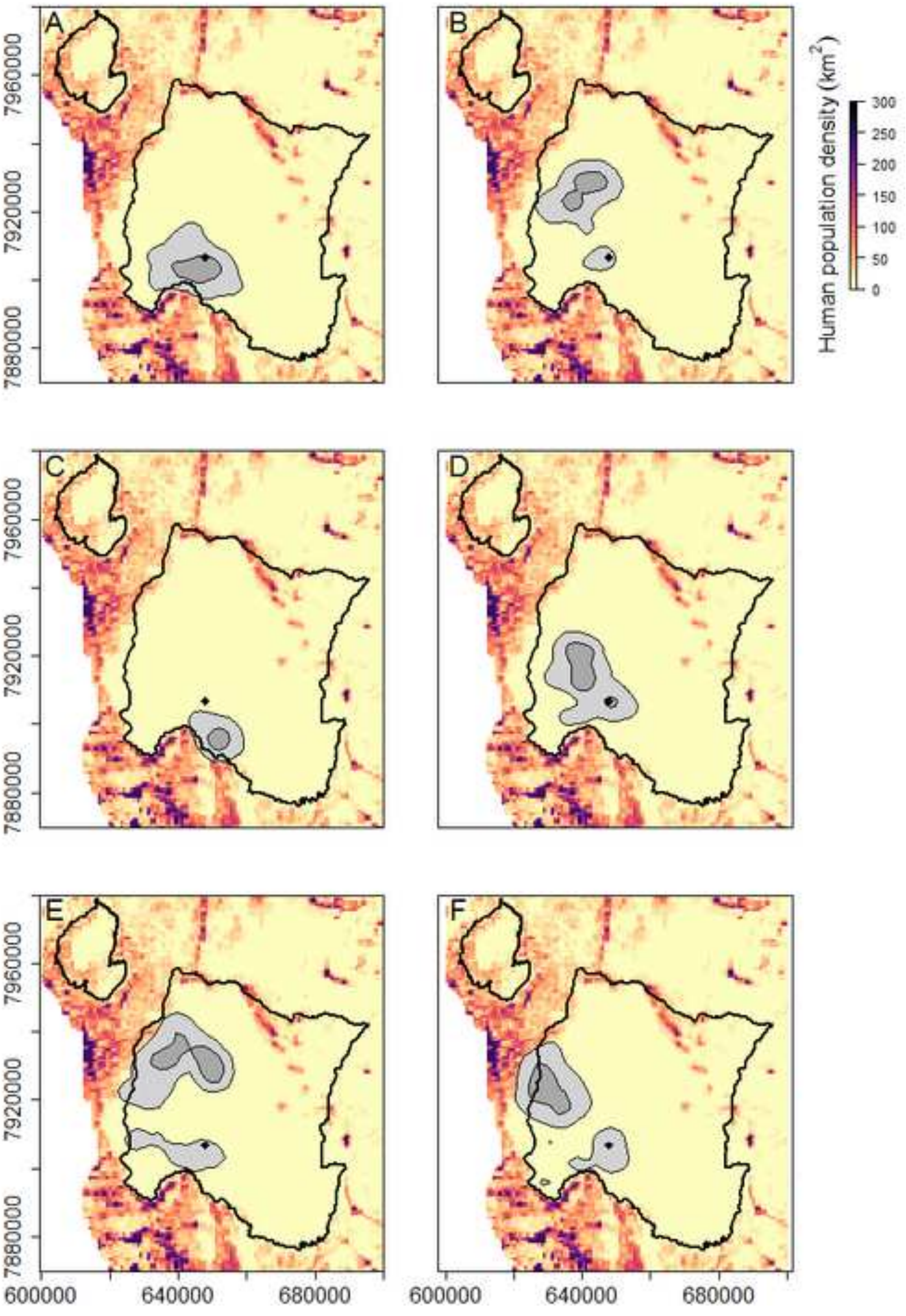

Click here to access/download;Figure;Fig7.tiff

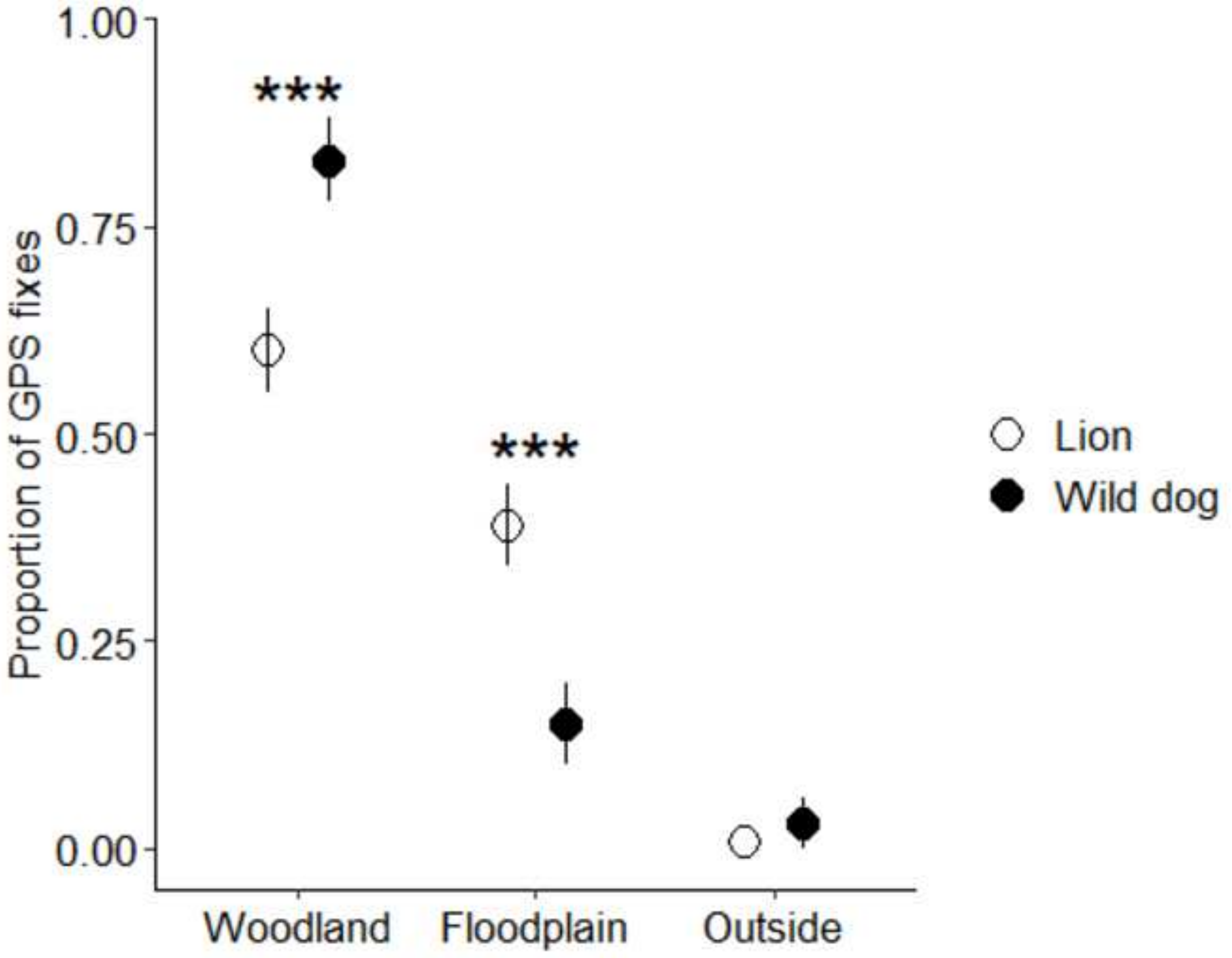

Figure8

Click here to access/download;Figure;Fig8.tiff

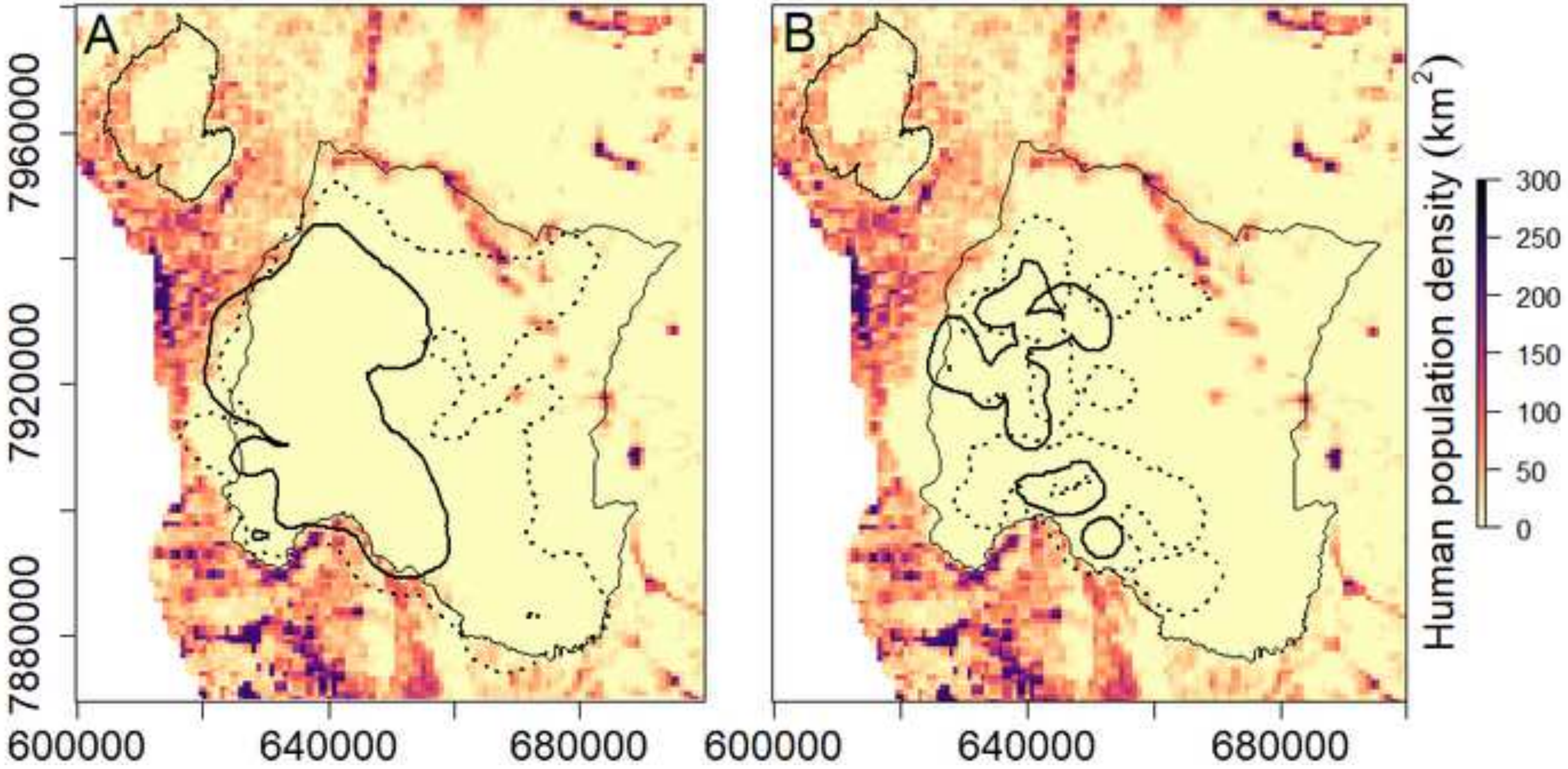

FigureS1
Click here to access/download;Figure;S1Fig.tiff

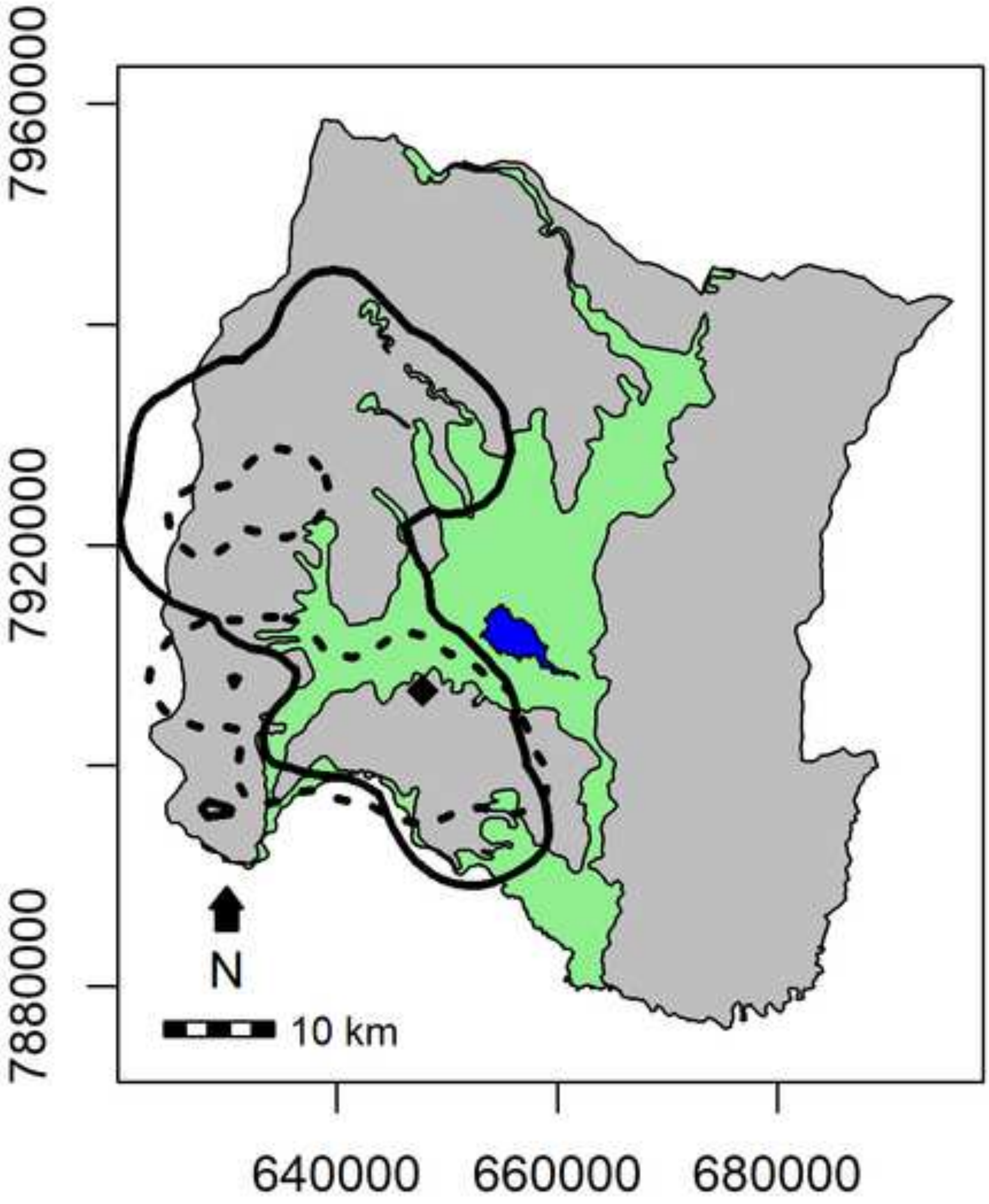

FigureS2

Click here to access/download;Figure;S2Fig.tiff

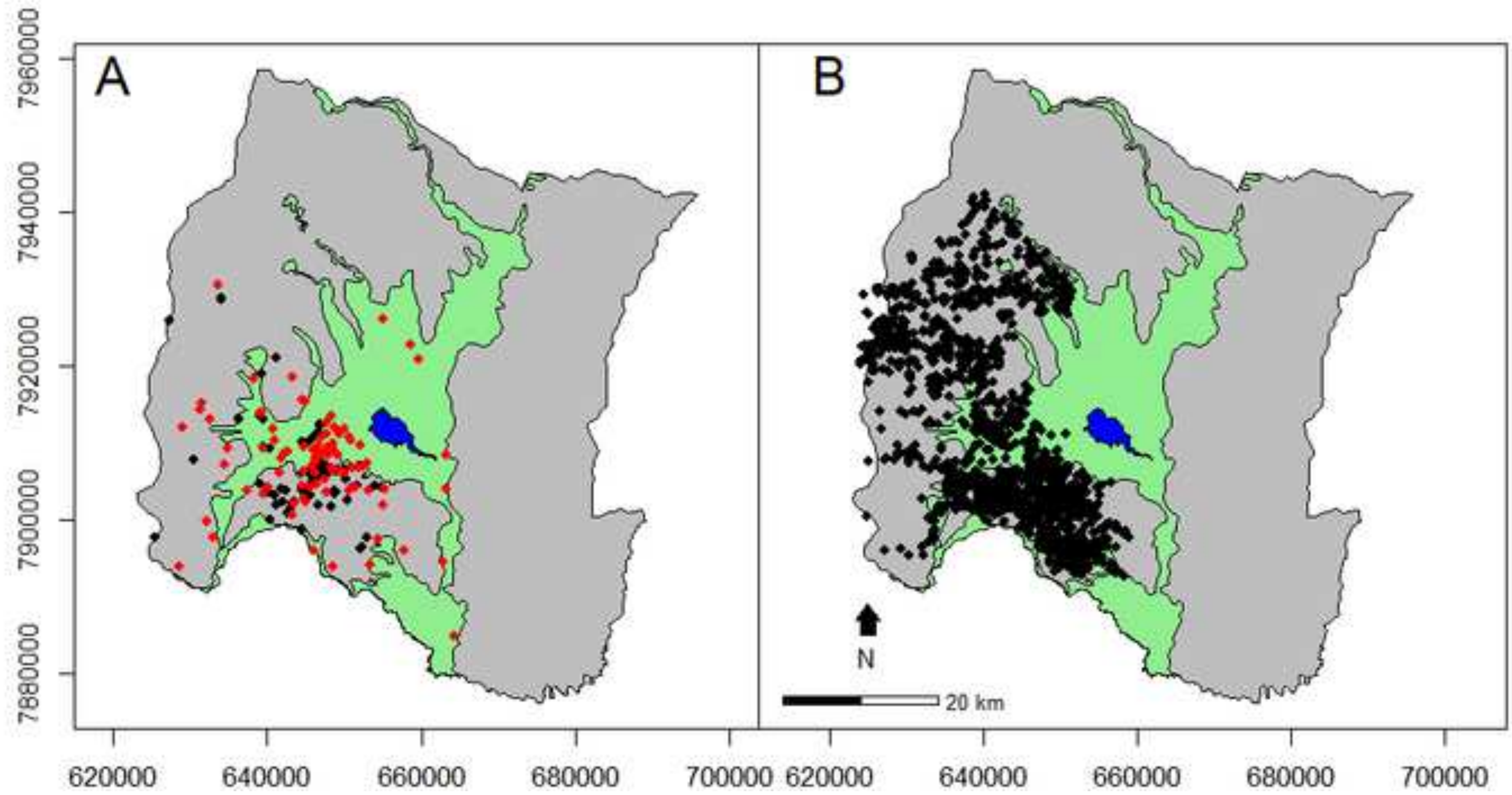

Click here to access/download;Figure;S3Fig.tiff

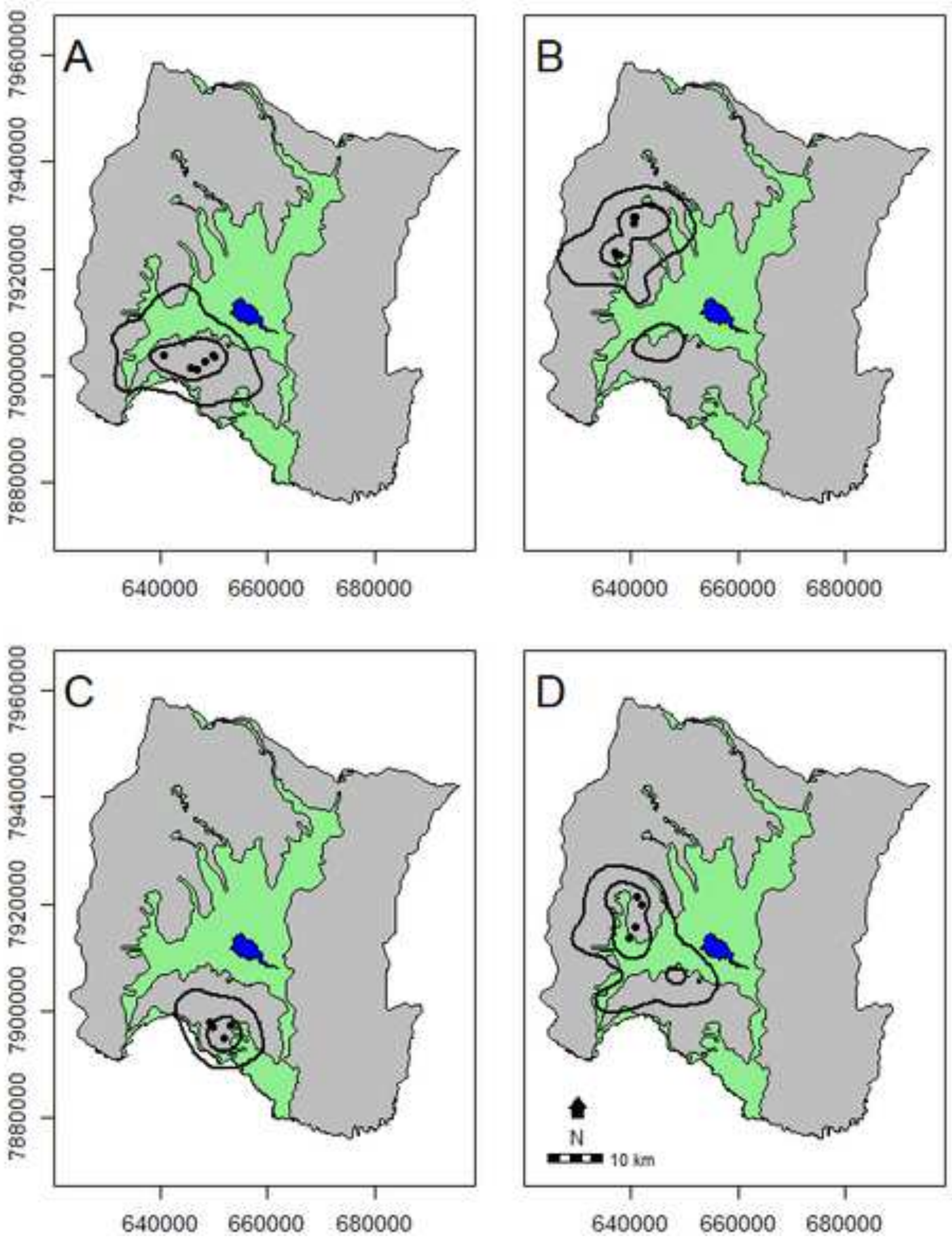

Click here to access/download
Supporting Information
Supplementary material_Final_Submission.docx
