# Supplementary material for: The successful reintroduction of African wild dogs (Lycaon pictus) to Gorongosa National Park, Mozambique
Source: PLoS One. 2021 Apr 22;16(4):e0249860. doi: 10.1371/journal.pone.0249860 (PMC8062010; doi:10.1371/journal.pone.0249860)
Supplement: Attachment — Submitted filename: Response to Reviewers _Bouley et al March 15 2021.docx [file pone.0249860.s011.docx]

Dear Dr. Bouley,

Thank you for submitting your manuscript to PLOS ONE. After careful consideration, we feel that it has merit but does not fully meet PLOS ONE’s publication criteria as it currently stands. Therefore, we invite you to submit a revised version of the manuscript that addresses the points raised during the review process.

**We very much appreciate the positive reviews and highly constructive feedback received from yourself and the team of reviewers. We are very pleased to be resubmitting a much improved and highly revised version of our manuscript, with revisions mainly centered on the introduction and discussion. We sincerely hope that this body of work as presented can help contribute to the science of reintroductions and also garner momentum for recovery of the species across the region. We hope that projects embarking on such work elsewhere are able to benefit from the lessons learned in Gorongosa.**

**Please note, we have uploaded a revised Manuscript (track changes and clean versions), and a revised Supplementary materials. Also, we removed a figure (the old Fig 5) from the main ms and moved it to Supplementary. Given we modified Fig 4 also, the order and label of figures has modified between figure #s 4 and 7. So all figures with their new respective assignments/numbering are uploaded and labelled as revised. Similarly for Supplementary materials figures.**

**Below, we have structured our responses to the Editor and three reviewers by including specific responses to each comment listed (our responses are in blue font color throughout). Line numbers refer to the line numbers in the track changed manuscript so that you are able to see where we have made the suggested changes.**

Your manuscript has the potential to make an excellent contribution to the literature of carnivore ecology and conservation generally and more specifically on their reintroductions. Your submission has received positive feedback as well as helpful suggestions for improvement from three reviewers with expertise in carnivore ecology, wild dog ecology and conservation, and the recovery of Mozambique's wildlife. The major area of divergence in the reviews surrounds the utility and presentation of the recovery indicators. Although I agree with Reviewer #3 that the manuscript is lengthy and could be more focused, I think an indicator or success criteria table could be useful for future reintroduction efforts and therefore may be worth keeping, but perhaps following revisions based on suggestions from the reviewers, including the minor detail of more clearly enumerating the criteria used. Below (and attached) you will find helpful comments from three reviewers to assist you with revisions.

**We thank you and especially reviewer 3 for the valuable suggestions made regarding the indicators we presented in Table 1. We have decided to retain the indicator table (see Table 1 in the manuscript) because assessments of reintroduction success are too often based on qualitative rather than quantitative measures (Reading & Clark, 1996; Breitenmoser et al., 2001) and there is little consensus about what constitutes a successful reintroduction (Seddon 1999). Thus, there is good reason for presenting the framework we have identified for evaluating reintroduction success (Table 1) that we have bolstered with relevant sources and examples from the literature. We strongly believe that explicitly stated indicators of success are critical to any recovery process and for guiding future interventions. However, and in accordance with the suggestions from reviewer 3, we have streamlined the indicators down to five categories and nine indicators (previously, we had presented 10 categories and 17 indicators). The comments from reviewer 3 were thus critical to reframing our approach by presenting baseline data to evaluate these main five indicator categories. We have also now included detailed support from the literature for establishing a criteria/framework for assessing reintroduction success and for determining which specific indicators are relevant to evaluating a reintroduction for wild dogs (L156-175). Essentially, these now specifically relate to (1) artificial group formation, (2) natural pack formation, (3) breeding, (4) survival and (5) population growth. The other indicator categories we used in the original MS have been excluded as indicators. However, we have retained some of these as potential mechanisms that could have influenced overall success. These specifically relate to lion avoidance and prey preference (L1076-1107) and human avoidance (L1108-1141). We think the indicators as now presented will be a useful reference tool for other studies wishing to develop their own indicators rather than deleting the table and having the indicators explained in the text of the introduction (as per Reviewer 3’s suggestion). We think that this approach will be agreeable with yourself and reviewers 1 and 2 as you seemed to be in favour of this indicator table. We now believe that the revised indicators are more concise, supported in the literature, are relevant to wild dog reintroduction and is a useful tool for other reintroduction studies.**

When submitting your revision, we need you to address these additional requirements.

1. Please ensure that your manuscript meets PLOS ONE's style requirements, including those for file naming.

**We have compiled the MS in accordance with PLOS ONE's style requirements as suggested.**

2. We note that you have stated that you will provide repository information for your data at acceptance. Should your manuscript be accepted for publication, we will hold it until you provide the relevant accession numbers or DOIs necessary to access your data. If you wish to make changes to your Data Availability statement, please describe these changes in your cover letter and we will update your Data Availability statement to reflect the information you provide.

**We confirm that we will be sharing data via FigShare once the MS is formally accepted. For the time being, the repository is stored in this private link:** [**https://figshare.com/s/a2eb4abc69381b301353**](https://figshare.com/s/a2eb4abc69381b301353)**. We have also reserved a DOI through figshare (10.6084/m9.figshare.13214699) that contains all the data (wild dog spatial, lion spatial, wild dog kills, lion kills, and R script) needed to complete the repository. We will “publish” this (i.e. the DOI will go live) if and when the MS is accepted. However, to fall in line with PLOS’ requirements, we have added the following statement in the MS (1215): “Data availability statement: Data are available from Figshare (DOI :10.6084/m9.figshare.13214699).”.**

3. We note that Figures 1, 6 and S1-S3 in your submission contain map/satellite images which may be copyrighted. All PLOS content is published under the Creative Commons Attribution License (CC BY 4.0), which means that the manuscript, images, and Supporting Information files will be freely available online, and any third party is permitted to access, download, copy, distribute, and use these materials in any way, even commercially, with proper attribution. For these reasons, we cannot publish previously copyrighted maps or satellite images created using proprietary data, such as Google software (Google Maps, Street View, and Earth). For more information, see our copyright guidelines: <http://journals.plos.org/plosone/s/licenses-and-copyright>. We require you to either (a) present written permission from the copyright holder to publish these figures specifically under the CC BY 4.0 license, or (b) remove the figures from your submission:
   a. You may seek permission from the original copyright holder of Figures 1, 6 and S1-S3 to publish the content specifically under the CC BY 4.0 license. We recommend that you contact the original copyright holder with the Content Permission Form (http://journals.plos.org/plosone/s/file?id=7c09/content-permission-form.pdf) and the following text. “I request permission for the open-access journal PLOS ONE to publish XXX under the Creative Commons Attribution License (CCAL) CC BY 4.0 (http://creativecommons.org/licenses/by/4.0/). Please be aware that this license allows unrestricted use and distribution, even commercially, by third parties. Please reply and provide explicit written permission to publish XXX under a CC BY license and complete the attached form.” Please upload the completed Content Permission Form or other proof of granted permissions as an "Other" file with your submission.  In the figure caption of the copyrighted figure, please include the following text: “Reprinted from [ref] under a CC BY license, with permission from [name of publisher], original copyright [original copyright year].”
   b. If you are unable to obtain permission from the original copyright holder to publish these figures under the CC BY 4.0 license or if the copyright holder’s requirements are incompatible with the CC BY 4.0 license, please either i) remove the figure or ii) supply a replacement figure that complies with the CC BY 4.0 license. Please check copyright information on all replacement figures and update the figure caption with source information. If applicable, please specify in the figure caption text when a figure is similar but not identical to the original image and is therefore for illustrative purposes only. The following resources for replacing copyrighted map figures may be helpful:
      i. USGS National Map Viewer (public domain): <http://viewer.nationalmap.gov/viewer/>
      ii. The Gateway to Astronaut Photography of Earth (public domain): <http://eol.jsc.nasa.gov/sseop/clickmap/>
      iii. Maps at the CIA (public domain): https://www.cia.gov/library/publications/the-world-factbook/index.html and <https://www.cia.gov/library/publications/cia-maps-publications/index.html>
      iv. NASA Earth Observatory (public domain): <http://earthobservatory.nasa.gov/>
      v. Landsat: <http://landsat.visibleearth.nasa.gov/>
      vi. USGS EROS (Earth Resources Observatory and Science (EROS) Center) (public domain): [http://eros.usgs.gov/#](http://eros.usgs.gov/)
      vii. Natural Earth (public domain): <http://www.naturalearthdata.com/>

**We assure you that the satellite image of Fig 1 and 6 (that depict human population density) (in the revised MS these are Fig 1, 5 and 7) have been derived for the Gorongosa-Marromeu landscape by two staff members of the Department of Scientific Services of the Gorongosa Project, based in Gorongosa National Park. These maps represent (1) original information that was not previously available, (2) that is not copyrighted and (3) that has not been published elsewhere. Additionally, we consulted with the two staff members from the Department of Scientific Services of the Gorongosa project to ensure there were no copyrights, and we have now specifically included this information in the legend for Figure 1 (L235). The map layers (i.e. shapefiles) of Africa and Mozambique (Fig 1) we obtained from this publicly available link:** [**https://open.africa/dataset/africa-shapefiles**](https://open.africa/dataset/africa-shapefiles) **that are not copyrighted. For the various boundary shapefiles of GNP and vegetation layers (Figures 1, 5, 7, S1-S3), these were constructed and made available by the Department of Scientific Services of the Gorongosa Project for this study. Thus, we believe we are not in violation of copyrights for the presentation of these maps and do not require any further permissions and/or details.**

4. Please amend either the title on the online submission form (via Edit Submission) or the title in the manuscript so that they are identical.

**We have now amended the online submission form to reflect the corrected title of the MS.**

5. PLOS requires an ORCID iD for the corresponding author in Editorial Manager on papers submitted after December 6th, 2016. Please ensure that you have an ORCID iD and that it is validated in Editorial Manager. To do this, go to ‘Update my Information’ (in the upper left-hand corner of the main menu), and click on the Fetch/Validate link next to the ORCID field. This will take you to the ORCID site and allow you to create a new iD or authenticate a pre-existing iD in Editorial Manager. Please see the following video for instructions on linking an ORCID iD to your Editorial Manager account: <https://www.youtube.com/watch?v=_xcclfuvtxQ>

**We have also amended online information to match the ms, and included a valid ORCID ID for Ms. Paola Bouley, the corresponding author.**

Additional Editor Comments:
- Given that you intend for others to be able to utilize the information you present for future translocations, it would be helpful for you to include a brief statement in your Ethics section on how translocations were conducted (e.g., in the presence of a vet, use of blindfolds, ear muffs), and perhaps include additional details (e.g., collar weight, drug combinations) as Supplementary material.

**We agree that these details would be beneficial, although these details are all captured in the multiple permits themselves (and these permits explicitly address ethics). However, we have described a brief version of these procedures on the translocation (L363-415). We have retained these details in the main text of the MS and not in the supplementary material.**

- L 591 and 595: "predate" should be corrected to "prey"

**Corrected.**

**Response to Reviewers**

Reviewer#1: This study by Bouley et al. is a thorough accounting of the successful reintroduction of endangered African wild dogs into Gongorosa National Park (GNP), Mozambique. GNP is rapidly becoming a paradigm for the large-scale, whole-ecosystem restoration, and carnivores remain the critical “missing link” in restoring a natural regime of ecosystem processes. As such, I consider this study is a very important contribution to the emerging literature on rewilding; this work serves both as an essential puzzle piece in the larger story of a large-scale rewilding project with few if any parallels worldwide AND as a step-by-step procedural guide to translocation and monitoring that will be practically useful for other estates that seek to (re)introduce wild dogs (or wolves, or other large carnivores for that matter). The authors outline a 16 (or 17, see below) point framework by which they benchmarked the success of the initiative, which is helpfully systematic and generalizable to other circumstances. On the whole, the work seems to have been carefully and rigorously done and is thoroughly described in the manuscript; I have some specific suggestions for improvement below, but pending the authors responses to these, I think that this study meet’s PLOS ONE’s criteria for publication and I would strongly advocate its publication once these and other reviewers’ comments have been addressed.

**We thank the reviewer for the comment on the applicability of the MS and have addressed each of your comments below.**

28: Instead of "carnivores", I would go with "these species", because the statement ("are threatened globally") really applies to large ("top") mammalian carnivores, as opposed e.g. other members of Carnivora (e.g., raccoons, skunks, domestic cats and dogs, etc.) or predatory animals more broadly.

**Because we deleted the first paragraph of the introduction (according to reviewer 3’s suggestion, this necessitated editing the abstract too. As such, this is no longer relevant**.

42-43: "First successful reintroduction of wild dogs into an open landscape" -- there have been many translocated dogs and an extensive literature on it, so this statement seems to hinge on the definition of "open" -- do you mean unfenced?

**We thank the reviewer for pointing this out. Yes, we are referring to unfenced systems and we have clarified the language to reflect this specific distinction (L154). We were directed to an additional study by reviewer 3 that was done by Jackson et al (2012) where a pack of wild dogs was reintroduced in Botswana. Thus, we have also amended our statement here and rather state that this is the first ever in Mozambique and the 2^nd^ on the continent (L162).**

73: I would add citation to Seddon et al. 2014. Reversing defaunation: Restoring species in a changing world. Science 345:406-412.

**Included (L161).**

79-80: Direct persecution of wild dogs by ranchers and pastoralists has been common throughout much of the continent, and seems like a curious omission here where listing drivers of population decline.

**We thank the reviewer for highlighting this obvious omission and have now included it in the list of threats with important references included (L93).**

99: In the context of this study, I think it's important to note here that recent work shows that the incompleteness of the carnivore guild has left an enduring ecological imprint on Gongorosa NP, per the recent study by Atkins et al. 2019. Cascading impacts of large-carnivore extirpation in an African ecosystem Science 364:173-177).

**Great point, this is now included (L117).**

Table 1: It is a little confusing that the Abstract refers to 16 indicators, but the Table shows 10 categories comprising what appear to be 17 specific indicators (if I count correctly).

**We thank the reviewer for picking up on this. Considering reviewer 3’s suggestions for a reduction in the number of indicators, we have now corrected this to nine indicators within five categories (Table 1, L205).**

Also, although a bit premature in that the results have not yet been presented, I think it would be helpful to include a column indicating if the indicator was achieved (which is yes for almost all of them, but would be helpful to have this as a quick look-up guide for those who might be skimming the paper).

**Originally, we had considered this and added this as a column when developing the introduction and conceptual framework for the study. However, because each indicator of success was met (even in our revised version of Table 1), we removed this column as we feel it did not add anything further to Table 1 (i.e. a column of all “ticks” or “yes” seems a bit redundant). Had there been more variation in the outcome of the indicators (i.e. maybe only half were met), then we would have included this column. Also, if we included a column for indicators met we would be presenting results prior to the methods and feel that would be premature. Considering these, we have decided not to include this column with a yes/no for each indicator and hope the reviewer will find this agreeable.**

137: "current" -- I would specify what year you mean by this, as this is otherwise ambiguous. A reference for this sentence would be helpful.

**Corrected and reference added.**

165: What other resident populations in the region are known?

**We have now included a summary (L258-269) of what is known regarding the extant population of wild dogs in the larger Gorongosa-Maromeu landscape, and across Mozambique.**

170-171: This is important information, but what is the reference point for these reductions in poaching? I.e., in 2018 as compared with what?

**We are comparing data between 2015 and 2018 and have clarified this in the MS, see L315.**

193-194: There is some grammatical hiccup in this sentence "reconnecting GNP to any other confirmed and no resident populations". Cf. my comment on line 165.

**Corrected (L258).**

273: A few more details on husbandry would be helpful, at least from my perspective. Were whole or butchered prey items offered? Do you know approximately what fraction of the prey were impala vs. waterbuck? Did dogs feed equally well on all foods offered? These details are not strictly essential but may help guide others attempting to follow your example.

**We have updated this section (L436). Briefly, we used whole waterbuck that were freshly culled and allowed the dogs to feed naturally on the carcasses (i.e. we did not butcher the carcass up).**

354: Can you be certain you detected *all* successful hunts? Seems unlikely to me.

**You are correct, and we apologise for this confusion. We have now amended this sentence (L539).**

356: How was "prey biomass" defined? E.g., did this include all of the species included in the aerial counts, including e.g. elephant and hippo that wild dog rarely if ever take down as well as species that might be too infrequent in GNP to be common prey? Or did you define a subset of counted species that are likely (in terms of size and relative abundance) to be eaten by wild dogs? Or did you only include the species that were actually observed to have been killed by wild dogs during this study as potential prey? These details are important for interpreting the selectivity measurements described in the next sentence. From Fig. 4, it seems that you only deemed a subset of counted species as potential prey, but specifying your criteria here is important. From Table S4, the dogs never ate a hartebeest, wildebeest, sable, or buffalo (Table S4), but only the latter three species are shown in Fig. 4. Which species were defined as potential prey or not will influence estimates of selectivity by influencing the inferred relative abundances of potential prey spp.

**We have clarified our definition of preferred prey for wild dogs in (L547). Specifically, we only used preferred prey based on the weight of those species (e.g. bushbuck, impala, reedbuck, oribi, kudu, nyala) and we also included any species we observed to be successfully predated upon by wild dogs (e.g. waterbuck and warthog). The inclusion of buffalo, sable and blue wildebeest in Fig 4 is because we also include lions in this figure (and they have predated these species).**

357-363: Similar to previous comment, a bit more detail about this selectivity analysis would be helpful -- most notably how you defined availability, but also how you converted your kill observations into data on utilization by dogs. It's not obvious to me that these calculations could be independently replicated without a bit more supporting information.

**We have now clarified this section as per our previous response (L552) and further edited this paragraph.**

460-465: These were non-parametric regressions? I think the test statistics output from lm() in R are all based on parametric assumptions. Can you clarify?

**We have now checked and can confirm that the test statistics from the *lm* output in R are based on parametric assumptions while chi-squared on non-parametric. Thus, we have now amended this sentence to accurately reflect this (L684) and thank the reviewer for pointing this out.**

491: Did the fight result in injury?

**Yes, this resulted in facial scars/injury on the younger male, but nothing serious. We have included this detail in the MS (L724).**

589: As noted above, I find it hard to evaluate the selectivity results based on the methods provided. More generally, although the selectivity data are important and interesting, they do not tell a complete story owing to the heavily skewed relative abundances of the prey species. I would include a 2nd panel in Fig. 4 that just shows the breakdown of prey by species in the form of a bar graph or pie chart; these data are presented in full in Table S4, but a graphical summary would be a helpful addition to the main text. I would also qualify the word "avoiding" on line 589 with "in relation to their availability" just to minimize the potential for confusion. Wild dogs ate a lot of waterbuck, which is interesting and perhaps surprising; that they were avoided (in relation to availability) seems to reflect the disproportionate relative abundance of waterbuck relative to all other prey (as noted elsewhere in the text).

**We thank the reviewer for this comment. We have now added a panel in Fig 4 (this panel b is summarised from data presented in Table S4) and removed the language surrounding “avoiding”. Indeed, waterbuck occur in very high numbers in GNP which is why we wanted to separate them in the Jacob’s index based on which habitat type these kills were observed in (i.e. Fig4a: woodland waterbuck vs floodplain waterbuck).**

Related: Was there any difference between packs in prey utilization (or selectivity)? Some experts believe that wild dogs have a strong sense of "tradition" in their prey preferences, and wondering if you saw any evidence of that as reflected in differences between packs.

**Interesting question but we can’t say that we noticed a traditional sense of prey preference. It probably isn’t occurring (at least for founders) because the founder individuals come from areas with relatively small bushbuck populations (e.g. in northern KZN, wild dogs prey predominantly on nyala and impala [Kruger et al. 1999, Vogel et al. 2019] and in Khamab there are also very few bushbuck). Thus, if there is a sense of tradition, then we would expect these founders to prefer nyala and impala but we can see that they prefer them less than bushbuck. Perhaps as time develops, we can compare founders vs non-founders (i.e. pups) to see if there is a difference there which could potentially be attributed to a sense of tradition/learnt behaviour (this requires a lot more data, especially from non-founding packs that are independent from the original founders).**

587-600 and throughout: I would use past tense consistently in describing results.

**Done**

609: This is a 117% increase (100% increase being a doubling), not a 217% increase.

**Corrected (L940).**

647: Descriptive stats on absolute number of kills in each habitat type would be helpful as a complement to (and arguably more important than) the statistical test. In Fig. S2, the red dots for lions eclipse the black dots for wild dogs, making it hard to discern where the wild dog kills occurred. As the focus of this paper is on wild dogs, I think it would be more helpful to bring the black dots to the foreground. Similarly, making this figure larger (e.g., by stacking the panels vertically instead of horizontally and expanding to fill the page margins) would be helpful.

**We thank the reviewer for this suggestion and have made the suggested correction regarding wild dog kill locations on top of lion kills and re-orientating S2 Fig (which is now S1 Fig in the revised MS). We have also added in the sample size of kills observed per habitat type (L1530).**

701-702: The prey data suggest that wild dog and lions are also avoiding each other through differential foraging patterns/prey selection (cf. parts of introduction about interference competition).

**Yes, indeed. We have now included dietary niche partitioning as a potential explanation for the coexistence of wild dogs with lions in GNP to date (L1076).**

707-708: A few more words about what this competition at dens looked like? Any observations of interactions between packs (or between WD and lion)? Ah -- never mind, I see this information in S1 Appendix. You should call that out here.

**We have called this out more directly here and referred to the appendix too.**

Discussion: Some brief comment on patterns of prey use/selection in relation to the larger literature on wild dog foraging would be helpful. It is surprising (to me, at least) that although wild dog feast on impala in much of their range, impala are abundant in GNP, and impala were among the feeds used in the boma, the dogs ate very few impala after being released.

**The vast majority of the carcasses we used in the boma were actually waterbuck (waterbuck males are much easier to cull and this has the least impact on wild populations too, see L437). Regardless, a few weeks after release impala and bushbuck were the dominant kills, and beginning after the denning document we began to document a shift towards waterbuck – maybe driven by the larger pack size? We will keep a close eye on this over time to determine what may be driving prey selection but it is worth a more intensive study (perhaps by collecting scat) to investigate the intricacies in wild dog prey selection in GNP. Because of our focus is presenting baseline data with the objective of relating this to reintroduction success, we have decided to not go into much more detail than what is already in the discussion relating wild dog prey preference in GNP to other studies. Because reviewer 3 raises issues of the length of the MS, we did not want to add further details in considering we have already added a further two paragraphs in the introduction. We hope that the reviewer will find this agreeable.**

Data Availability: I do not see a Data Availability Statement. Authors should include a statement affirming their adherence to PLOS data availability requirements (<https://journals.plos.org/plosone/s/data-availability>) and deposit these data within a repository or as supporting information files. This is important for maximizing reproducibility and ultimate scientific and conservation impact of the research.

**Same response that we provide to the editor earlier on in this response document: We confirm that we will be sharing data via FigShare if and once the MS is accepted. For the time being, the repository is stored in this private link:** [**https://figshare.com/s/a2eb4abc69381b301353**](https://figshare.com/s/a2eb4abc69381b301353)**. We have also reserved a DOI through figshare (10.6084/m9.figshare.13214699) that contains all the data (wild dog spatial, lion spatial, wild dog kills, lion kills, and R script) needed to complete the repository. We will “publish” this (i.e. the DOI will go live) if and when the MS is accepted. However, to fall in line with PLOS’ requirements, we have added the following statement in the MS (L1214): “Data availability statement: Data are available from Figshare (DOI :10.6084/m9.figshare.13214699).”**

**Reviewer #2:** This paper has largely been a pleasure to read. It’s well written and informative, and gives sufficient detail without being overwhelmingly bogged down in unnecessary statistics, which makes it very accessible to readers. It also represents a short-term success story for reintroduction of African wild dogs into an open ecosystem which is a valuable contribution to the literature (and to conservation). I have recommended it for publication with a few minor changes.

**Thank you, we very much appreciate these comments.**

Most of my comments are on the PDF. A few key points are expanded below.

**To assist in our response, we have extracted those comments and suggestions from the PDF and placed them in this response document so that the editor and reviewer can see that we have addressed all the points:**

- Online ethics statement - text below is a direct duplicate of text above. Remove repetition.

**We have ensured that the online upload only has one version and isn’t duplicated.**

- Describe where data found - Also duplicate. Perhaps just a quirk in the system!

**We have ensured that the online upload only has one version and isn’t duplicated.**

- L8 - This is a longer list of authors in a different order from that listed in the summary at the start of this pdf

**We have now corrected this to reflect the five authors and the corrected order. Note that the author list here (L10) is the correct list and order.**

- L16 - This is also different to the corresponding author info given in the summary

**We have now corrected this in the online upload. Note that this is the email address for Paola Bouley who is the first author.**

- L32 - recent focus?

**Deleted following edits to the abstract.**

- L33 - hereafter 'wild dogs' (since you no longer use African wild dogs).

**Done.**

- L43 - remove 'the'

**Done.**

- L52 - I may be wrong, but usually you don't need to include words that are already in your title in the key words (they are already picked up by search engines).

**Thank you for pointing this out and we think you are right. We have now edited the key words to include conservation translocation while we have deleted Gorongosa National Park and *Lycaon pictus* (as these appear in the title and abstract text) (L78).**

- L86 – add “of”

**Done.**

- L165 - Please somewhere include a very brief summary of where other resident populations are in Mozambique (including distance from GNP).

**As done for reviewer 1, we have now included a summary (L258-) of what is known regarding remaining population in the larger Gorongosa-Maromeu landscape.**

- L172 - that the threats that lead to

**Done – note that this section has been moved here from the old section (Determining the status of wild dogs in GNP) to this section (Reintroduction suitability assessment).**

- L180 - The correct program name is: Range Wide Conservation Program for Cheetah and African Wild Dogs. (This section might be a good place to include the info on other resident populations and their proximity to GNP, to clearly show that natural recolonization was extremely unlikely, despite the vast distances the species is capable of moving).

**Corrected the full name (L284). We have included the section on other resident populations and their proximity to GNP in the earlier section in L258.**

- L181 - (RWCP) since you then use this acronym below.

**Done.**

- L211 - Please explain your due diligence in ensuring these were appropriate from a genetic standpoint (not just their genetic diversity - but their genetic suitability for Mozambique).

**This is a very important point and we thank the reviewer for highlighting that this needs to be much clearer in the MS. We have now provided a detailed accounting on how wild dogs originating from the “S” genotype from southern Africa falls within a large admixture zone between the S and E genotypes that likely occur in Mozambique (L324-). Thus, if any extant wild dogs are present in the larger Gorongosa-Marromeu landscape, then the introduced founders from South Africa should not have any deleterious effects on the genetics of extant wild dog packs.**

- L220 - Why was a naturally formed pack taken out of a game reserve for this translocation? Explain why they couldn't they stay in Khamab Kalahari GR.

**Based on a wild dog carrying capacity assessment conducted by reserve management and the Endangered Wildlife Trust, it was concluded that Khamab had an excess number of wild dogs within the fenced perimeter of the reserve. Additionally, these individuals in the identified pack for removal were very likely unrelated to those already taken to GNP. Thus, they were deemed as the most viable pack from the metapopulation in South Africa for reintroduction to GNP which was boosted by the pack comprised of 10 individuals that would likely be able to adapt to the new environment in GNP. We have added details on this at L356-.**

- L237 - And presumably where the animals could be fed and watered without any contact with people? Please add / explain.

**We have added an extra sentence to clarify (L370-). Zero contact with people is not possible (the wild dogs in the boma need to be fed and water trough emptied and resupplied regularly) but all effort was made to ensure minimal contact and as little noise was made as possible during daily enclosure monitoring and maintenance checks.**

- L244 - Please give more detail on doses and top ups etc. Also give the time of the flight (more relevant than the distance).

**Added to each translocation’s details (L387 and 407).**

- L261 - My understanding is the rabies vaccines need a booster dose to be effective in wild dogs. How did you do that and if you didn't, why not? (I see below you mention booster for CDV - is that no longer necessary for rabies)?

**We followed veterinary advice from the vets from South Africa and Mozambique and standard operating procedures from the Wild Dog Advisory Group of South Africa (WAG-SA) whereby rabies vaccines are a once off whereas CDV requires a top-up/booster vaccine.**

- L274 - Remove 'Importantly'

**Done.**

- L278 - And rabies?

**As explained two comments above, no rabies booster was given following veterinary and WAG-SA advice. Dogs are vaccinated once per year only.**

- L333 - dogs / individuals

**Done.**

- L336 - We used two measures for population density

**Done.**

- L338 - and because the pack is the unit of reproduction

**Done.**

- L349 – date?

**No longer relevant due to rewriting of this sentence.**

- L354 - 'We recorded details for all successful hunts observed, or carcasses located that were confirmed to have been killed by wild dogs (you cannot have recorded every hunt all the dogs ever did, so you cannot say you recorded 'all successful hunts')

**We apologise for the confusion and ambiguity because we certainly did not record all successful hunts. Reviewer 1 raised this same point and we have now corrected in L539-.**

- L368 - give average doses

**Done.**

- L395 - This last sentence better suited to discussion (or perhaps results) than methods.

**Moved to results.**

- L401 - Don't abbreviate in title (and at first mention)

**We have now removed the abbreviation of KUD and replaced with Kernel Utilisation Distribution.**

- L413 – then

**Done.**

- L426 – estimated

**Done.**

- L427 - what are you using to define focal range? If that's the 50% isopleth, you have previously called it 'core territory' so perhaps use the same terminology to avoid confusion. Or explain. Otherwise you need to define focal range and explain how you calculated it.

**Thank you for highlighting this confusion. This “focal range” actually refers to the territory of the specific pack in question when evaluating its level of overlap with all its conspecific neighbouring groups – we have now clarified this in the text (L614). In the following sentence, we also explicitly state that we evaluated overlap at both the 50% and 95% territory areas. However, to avoid further confusion, we have added the definitions for the total territory area (i.e. 95% isopleth) and the core territory area (i.e. the 50% isopleth) in L614.**

- L433 - Presumably over the whole 28 months (since you didn't split the data for lions). But worth adding that.

**Yes, that’s correct. We have now added this.**

- L438 - at the broadest possible scale!!

**Not necessarily the broadest possible scale as this would then relate to habitat use across the entire range of the species (i.e. Johnson’s 1^st^ order selection), whereas in our study this level of habitat use corresponds to Johnson’s 2^nd^ order of selection (i.e. home range of the individual or group of individuals). We have clarified this in the text (L623).**

- L441 - Is this referring to multiple litters within a single pack in the same year? If yes, perhaps just briefly note that. Otherwise it could be referring to packs using the same den site in different years.

**Done (L784).**

- L484 - Perhaps start with a brief reminder of how many dogs they were to start with (of each gender). (For both founder packs)

**We considered this but decided not to incorporate here because this is repetition of what has been detailed in L387-417 for both packs and this is also clear in Fig 2.**

- L493 - and the 8 males became part of the same group again?

**Yes they did. Now clarified in ms.**

L500 - including the dominant pair? (Obviously it's the alpha female that determines if it is considered the same pack)

**Only the original alpha female (clarified in the ms) but the alpha male changed pack (moved to the Cheza pack in March 2020 - see the split from Gorongosa pack in Fig 2 and Table S3).**

- L511 - again, was this female the original alpha?

**No, and we have clarified this now in the ms.**

- L518 – remove ‘to’

**Deleted.**

- L525 – remove ‘her’

**Deleted.**

- L531 - in how many packs over how many pack years?

**Completely rewritten the “Breeding” results paragraph for clarity as per this reviewer’s comments) L768.**

- L533 - this sounds like you just didn't bother to record 2018 litters (!) but I suspect there weren't any litters then. Can you just rephrase to clarify. It also sounds like you recorded 11 pregnancies in each year... which is clearly not possible, so please re-phrase! (Not everyone will look at the table)

**Completely rewritten the “Breeding” results paragraph for clarity as per this reviewer’s comments. L768.**

- L534 – over both years?

**Completely rewritten the “Breeding” results paragraph for clarity as per this reviewer’s comments. L768.**

- L535 - emerging or being born?? That's important, because that's usually about a 2.5 / 3 week difference.

**Completely rewritten the “Breeding” results paragraph for clarity as per this reviewer’s comments. L768.**

- L537 - This should be S2 not S3. **S2 Table. Birth details.** Pregnancies and confirmed litters for eight female wild dogs in Gorongosa National Park between June 2018 and September 2020. **S3 Table. Group association dynamics.** Details of events that resulted in individual founder wild dogs changing their group association in Gorongosa National Park from the time of the first reintroduction in June 2018 until September 2020. AM = adult male, YF = yearling female and AF = adult female.

**Thank you for picking this up and yes we did mean it to be S2 Table, now corrected in the MS.**

- L530-537 - I think this whole paragraph could do with re-writing. It's hard to follow and not all that clear how many females actually had litters in which years and what your statistics refer to. Not everyone will be able to look at supplementary info, so this needs to be clear without reference to the table.

**Completely rewritten the “Breeding” results paragraph for clarity as per this reviewer’s comments L768.**

- L542 - remove i.e. from all of these

**Removed throughout this section**

- L543 - this is probably superfluous. It's an uncommon measure anyway (to nine months) and unnecessary since it was the same at six months and one year.

**Agree and we have deleted now**

- L566 - This sentence is a (less informative) repetition of the sentence on lines 570/571

**Deleted**

- L567 - assume this is including pups, in which case perhaps just make that clear, as you say previously numbers will only reflect adults and yearlings unless specifically mentioned to include pups.

**Yes, in this particular instance, this count does include pups which we have now clarified**

- L594 – comprised (past tense)

**Done**

L623 - remove 'at some stage in their early release'??

**Deleted**

- L628 - how are you defining 'core area' vs 'core territory'?

**As stated earlier, and to avoid further confusion, we have added the definitions for the total territory (i.e. 95% isopleth) and the core territory (i.e. the 50% isopleth). In this example, we have removed the word “areas” and replaced with “territories” so that our terminology is consistent across the MS relating to territory definitions. See L604-**

- L639 - I think this is the first time these dispersal group names have been mentioned - perhaps they should be mentioned in the text if they are used in a figure? (And likewise the other pack names which I think are only previously mentioned in an earlier figure).

**We have now added their names here (L887), like we originally had for the packs (also Fig 2 and S3 Table).**

- L690 - if a pregnancy (and denning behaviour) were observed as you said above, they did breed - just unsuccessfully if no litter emerged.

**The discussion has been largely rewritten as a natural consequence of rethinking the indicators as per Reviewer 3’s suggestions. We did attempt to address this issue in L1046.**

- L695 - As could the python you observed...! (Likely to have been a small litter as a first time alpha female and not unlikely that a python could have taken 3 / 4 pups). On which note - was she a first time alpha? I dont think it is mentioned anywhere whether these females had previously bred before, and that would be important information to include in the relevant place.

**The discussion has been largely rewritten as a natural consequence of rethinking the indicators as per Reviewer 3’s suggestions. We do not know if the females in either founding pack were first time mothers and to prevent speculation we have decided not to address this directly in the revised discussion.**

- L702 – and prey selection?

**The discussion has been largely rewritten as a natural consequence of rethinking the indicators as per Reviewer 3’s suggestions. However, we have made specific reference to dietary niche partitioning between wild dogs and lions as a potential mechanism that influenced success of the reintroduction (L1082).**

- L716 - Please explain reasons for these multiple pregnancies (i.e. siblings / dominance hierarchy not yet firmly established / reduced pheremonal expression of subordinate breeding (why) etc?

**While we can only hazard a very rough guess (high prey availability and low inter- and intra-specific competition, this is something that needs to be explicitly tested and evaluated (maybe across multiple populations as this has been recorded elsewhere, albeit quite rarely).**

- L743 – the / one

**We have rewritten these sentences and this edit is no longer relevant.**

- L747 – they are

**We have rewritten this sentence and this edit is no longer relevant.**

- L751 - this sentence isn't clear? Change ending to 'may be viable' / 'could be preferred' ? And if it is likely to be viable to take individuals from closer by, why wasn't this done in the first place?

**The discussion has been rewritten so this is no included here but we have provided details earlier as to the due diligence in founder animal selection as it relates to genetics, distance from GNP and availability (L324).**

- L762 - consider replacing endangered with threatened or vulnerable?

**We have rewritten this sentence and this edit is no longer relevant.**

- L768 - not really a paradigm. Global trend?

**We have rewritten this sentence and this edit is no longer relevant.**

- L772 - Please include in this discussion that the priority for restoration of wild dogs should always be on natural colonization (promoting site security in areas of connectivity / community conservation work etc) and physical translocation of individuals should be a last resort where natural supplementation isnt possible (as in this case).

**We thank the reviewer for pointing out this omission and have now added this L1183**

I would like to see a little more explanation of the due diligence conducted to ensure the genetic suitability of the reintroduced stock (i.e. your process of determining that it was suitable to bring dogs from SA into Mozambique).

**In line with our earlier response to this concern, we reiterate it here: This is a very important point and we thank the reviewer for highlighting that this needs to be much clearer in the MS. We have now provided a detailed accounting on how wild dogs originating from the “S” genotype from southern Africa falls within a large admixture zone between the S and E genotypes that likely occur in Mozambique (L324). Thus, if any extant wild dogs are present in the larger Gorongosa-Marromeu landscape, then the introduced founders from South Africa should not have any deleterious effects on the genetics of extant wild dog packs.**

The Breeding section in the Results needs re-writing slightly. Please see my comments. It’s not incorrect, just very confusing (I spent a while wondering how there could have been 11 pregnancies in each year!!).

**Understood, and additional reviewers found this section confusing too. We have re-written this section (L768) and hope that it is now clear.**

I think the discussion could be a bit fuller. You observe some very interesting things but given them no space in the discussion. This is very focused on the success of the reintroduction, which is valid, being the purpose of the paper, but please see my comments in the attached PDF asking for a little more interpretation of some of the more interesting / important findings.

**Where requested we have now provided further details. We have still retained the key focus on how these patterns of pack formation, breeding, survival, and population growth relate to reintroduction success as this is the focus of the MS.**

**Reviewer #3:** Comment to manuscript PONE-D-20-35800 “Call of the wild: the successful reintroduction of African wild dogs to Gorongosa National Park” by Bouley and colleagues.

The authors report of a successful reintroduction attempt of African wild dogs to Gorongosa National Park, from where wild dogs have been absent since the civil war. The authors identify 16 criteria that they deemed appropriate to assess reintroduction success. The manuscript is well written and this is a success story that needs to be told. There is an incredible amount of very meticulous work that was put during fieldwork and in the preparation of the manuscript.
**Thank you for your in-depth and thoughtful review of the MS.**

The manuscripts mainly describes observations and data collected in a rather detailed and reliable manner and I do not have major comments. This meticulousness in describing the events is, however, a double-edged sward:
1) The manuscript is incredibly long, instead of identifying a couple key processes that are really relevant to assess reintroduction success. In its present form the manuscript is not focused. Please refer to comment below re some of the 16 criteria.

**We thank the reviewer and agree that it is on the longer side. While we have tried to be more concise throughout the MS (see all the tracked changes that have been made in the resubmission and the reduced number of indicators – see Table 1), we have also been careful to see to the addition of two new paragraphs in the introduction to explain the rationale for the indicators (as per this reviewer’s comment further below) and still unpack the results in a more meaningful way. This relates to this reviewer’s 4^th^ comment below (relating to why the reintroduction was successful) where for each revised paragraph in the discussion, we have also provided a “lesson” on what this means for future wild dog reintroduction attempts. We also consolidate our results of the soft-release approach in pre-release enclosures with recommendations for future reintroductions. Considering these important additions as per this reviewer’s suggestions, we hope that you will find this agreeable and the length of the MS justified. We believe this MS does reflect the highly complex and intensive process of reintroducing an endangered species (wild dogs specifically) and that closely monitoring the population outcomes is important. A clear understanding of process (from start to finish) as well as local context (in this case GNP) is needed.**

2) This is one of the few successful reintroduction events (despite not the first; see for example Jackson et al. 2012 Wildlife Research) in an unfenced area. This makes it even more important to discuss why this attempt was successful and why others were not. Differences in release method? Differences in presence/abundance of other carnivores? Differences in habitat characteristics? Differences in prey availability? Unfortunately there is no mentioning of all these in the discussion. On this note, it is important to note that a large unfenced area is probably not very different from an unfenced one in the first phases of reintroduction, of course the situation is different for some fenced parks in SA that are smaller than the size of an average wild dog pack home range.

**We thank the reviewer for pointing out what is now a fairly obvious omission from our side. Considering this, we have now used previously included indicators (i.e. space use, habitat use, diet, humans and management) as possible mechanisms that influenced success rather than as pure indicators themselves. In this regard, we found little support in the literature for these aspects as indicators but rather as behaviors/mechanisms that could facilitate a successful reintroduction. Thus, while we still conclude a successful reintroduction (note, the remaining five indicator categories in Table 1 were all still met), we have now used results on space use, diet and management to explain why the reintroduction has been so obviously successful. See specific examples of this in L949-.**

**Unfortunately, we cannot directly compare the results of our study to the only other wild dog reintroduction to an unfenced area, i.e. Jackson et al. (2012). While Jackson et al. (2012) used a reintroduced pack’s movements for their study, they did not quantify the reintroduction effort itself, giving no information relating to pre-release enclosure bonding, timing of release, dispersal and natural pack formation, breeding, recruitment, survival and population growth. Thus, while we would have used such details to compare to our study, we cannot make valid comparisons with the only other reintroduction into an unfenced system.**

3) In my opinion, few elements are key and stand out and I suggest focusing on these instead of trying to report on everything that has been collected. These are the topic in the result section under “breeding”, survival”, “population size”, and “territory size and overlap”. For instance the high number of subordinate females reproducing is unusual for any well-established natural population**.**

**While we have focused our indicators on these exact topics (except territory size and overlap, see Table 1) we have retained some of the additional elements (space use, habitat use, diet) within the results to offer some insight in the underlying conditions that have influenced reintroduction success. This is important as spatial and dietary niche partitioning could explain the low degree of influence of lions on wild dogs in GNP, despite their obvious threat and we had not previously considered that as an important mechanism for success (in the last MS we thought of them purely as indicators). We believe that after having gone through the literature, we have strong support for the five indicator categories (and their associated nine key indicators) (L205 and Table 1) and behaviors and mechanisms that could influence these indicators (L156--). Consequently, we have retained data related to the five indicator categories (to evaluate success) and aspects of space use, habitat use, diet and prey preference (to provide data on potential mechanisms of success) within the results and discussion sections. Importantly, we have been careful to frame indicators as those only relating to artificial pack formation, natural pack formation, breeding, survival and population growth.**

4) I do not agree with many of the 16 criteria (see below) but I also think that the authors should move away from those. That the reintroduction was successful is irrefutable, the authors do not need 16 (questionable) criteria to prove that. Instead they need to say why this attempt was successful and what we should learn from this. Under which circumstances can we expect reintroduction to work and under which reintroduction may not work? This is the lesson that conservation needs to take home.

**We thank reviewer 3 for the valuable suggestions regarding the indicators we presented in Table 1. This comment (and each pertaining to each indicator) has greatly helped us to narrow our focus around the indicators and obtain support for their inclusion or removal. Thus, fundamentally, we have decided to retain the indicator table (see Table 1 in the manuscript) because assessments of reintroduction success are too often based on qualitative rather than quantitative measures (Reading & Clark, 1996; Breitenmoser et al., 2001) and there is little consensus about what constitutes a successful reintroduction (Seddon 1999). Thus, there is good reason for presenting a quantitative framework used to evaluate reintroduction success (Table 1). Further, we have now bolstered these remaining indicators in Table 1 with relevant sources and examples from the literature.**

**We strongly believe that explicitly stated indicators of success are critical to any recovery process and for guiding future interventions. However, and in accordance with the suggestions from reviewer 3, we have streamlined the indicators down to five categories and nine indicators (previously, we had 10 categories and 17 indicators). The comments from reviewer 3 were thus critical to reframing our approach by presenting baseline data to evaluate these main five indicator categories. We have also now included detailed support from the literature for establishing a criteria/framework for assessing reintroduction and for determining which specific indicators are relevant to evaluating a reintroduction for wild dogs success (L156---) Essentially, these now specifically relate to (1) artificial group formation, (2) natural pack formation, (3) breeding, (4) survival and (5) population growth. The other indicator categories we used in the original MS have been excluded as indicators. However, we have retained some of these as potential mechanisms that could have influenced overall success. These specifically relate to lion avoidance and prey preference ((L1076--), and human avoidance (1108---). We think the indicators as now presented will be a useful reference tool for other studies wishing to develop their own indicators rather than deleting the table and having the indicators explained in the text of the introduction (as per Reviewer 3’s suggestion).**

**We have also ensured that in the discussion, we put forward lessons from the indicators that will be useful for future reintroduction efforts [L1105---]. We believe that the inclusions of these provide important useful lessons for the larger conservation world to take home.**

A few general comments to the introduction and to the 16 categories

- the first paragraph appears inappropriate and too generic. I suggest deleting it completely and starting directly wit the second paragraph, which is more focused and pertinent to the topic of the manuscript.

**Done.**
- The paragraph starting with “The recently restored Gorongosa National Park (GNP)” should then be moved in second position and the paragraph starting with “The African wild dog (Lycaon pictus)” in third position. After this paragraph there is the need to add a paragraph describing the indicators used to assess successful reintroduction (see next comment).

**Done. Description of GNP (is now before wild dog paragraph.**
- There is a total lack of hypothesis. What and why should be expected to be a good measure for reintroduction success? Which parameters should be considered? I see this info is summarized in Table 1, but I suggest a paragraph describing the broad categories used, and why these are important indicators. For instance:

**We thank the reviewer for this and on consideration agree that the rationale for the indicators is missing. We have reviewed the literature again included a paragraph on why indicators for evaluating success are necessary (L176--). Having consulted the reviewer’s comments and the literature relating to each indicator, we have removed more than half the indicators and retained just five of the original 10 indicator categories (L205).We also provide specific hypotheses of what we expected to occur (L183--).**
1A and 1B: how does this apply to phase two since an entire pack has been translocated?

**Despite the second pack being naturally formed, field observations from Khamab suggested it was a recently formed pack. Thus, there is the potential for low pack cohesion and one could expect pack disruption in a new environment. To clarify these points, we have edited the working of indicator 1A (Table 1) to reflect that bonding is important for the first pack while pack cohesion is important post-release (relevant to both founding packs).**
4B: why survival of 50% of founders should be used as indication of successful reintroduction? Is this because this matches survival rate of founder individuals in the wild? Positive pop growth depends on mortality (of founders) and birth, not only on mortality of founders.

**No longer relevant, indicator removed.**

**The 50% survival rate has been removed after consultation with the literature which just defines survival of the release generation (see references for indicator 4A in Table 1 and L157--)**
5A: what shall be understood with “population size increases significantly”, what has to be considered a “significant” increase and why?

**Removed “significantly” from Table 1 for indicator 5A.**
6A: I disagree. Diet has nothing to do with successful reintroduction. What if reintroduced dogs specialize on small rodents (I know, silly example) and still thrive? Would you consider reintroduction to be unsuccessful?

**After consideration, we agree with the reviewer and in line with our previous comments on the streamlined indicators, we have removed diet and prey preference as indicators. This is because dietary niche partitioning could act as a mechanism for success rather than purely an indicator of success.**
7A: is a trivial consequence of 2B, so redundant.

**This is a valid point. We have removed spatial expansion of the population as an indicator but retained the result (L917-923) because we wish to be able to help inform management in GNP on potential for range expansion for the population. While we agree with the reviewer that this is not an indicator of success, it is an important consideration for park management that is considering further reintroductions and potential release locations in GNP.**
8A: Again, there is no rational to use prey/diet as a metric to assess successful reintroduction.

**Agree. Removed diet and prey preferences as indicators but retained their analyses in the MS to help us with assessing potential mechanisms the facilitated a successful reintroduction (i.e. dietary niche partitioning).**
8B: Makes no sense. I know that lions are among the main causes of mortality in wild dogs, but what if the reintroduced dogs, despite using areas used by lions, thrive and adults and pups survive? Would you consider this as a failed reintroduction? Important is to quantify lions density to allow comparison with other areas where reintroduction has also been attempted. But I don’t see the need to calculate overlap or avoidance.

**Agree that this is not a relevant indicator any longer but it is a critical factor that can competitively exclude wild dogs, especially over a time, such as that experienced in Serengeti National Park (Swanson et al. 2014). Thus, we have included lion avoidance strategies as potential mechanistic explanations for why wild dogs have done well in GNP (L192 and L1076). We account and discuss the density of lions as a potential contributing factor in this (L1076) and relate lion density effects on wild dog persistence to that in the Serengeti .**

9A: similar as for 8B.

**Removed as indicator from the MS (see Table 1).**
9B: It is irrelevant of what they die of, as long as 4B and 5A apply. Also, how is “minimal” mortality quantified? Additionally, the authors state that “threats that the lead to the historical extirpation of wild dogs in GNP have been alleviated (l. 172). So this means that one of the criteria to assess successful reintroduction applies is even before reintroduction starts….

**This is a valid point and we have since removed this indicator from Table 1. We do provide data on mortality in the results and discuss effective human avoidance by wild dogs (L1108). We did this because human-induced mortality is likely the main cause for decline in wild dog range across the African continent. Thus, this type of mortality can be additive to their natural mortality rates and could drive populations towards extinction. Hence, the most sustainable populations of wild dogs in Africa are found within protected areas. We therefore believe that discussing the management related influences on reintroduction success is critical not just in alleviating the factors that likely caused the original decline but also in the sustained effort to keep snares off of wild dogs.**
10A and 10B: Makes little sense. For instance, it is up to the Restoration Project managers to decide who should oversee the reintroduction. So basically by deciding if they want to hire “wildlife veterinarians of national origin” or “wildlife veterinarians from abroad” they indirectly decide (according to your categories) whether the reintroduction is successful or not?

**We have removed these from the indicators but we have retained a detailed discussion on the importance of these as potential mechanisms that influenced the success of this reintroduction (L1117---). We think this is the more appropriate context for the inclusion of how management relating to removal of snares and community engagement has likely helped this reintroduced population.**

There is indeed the need to justify the choice of these categories! Or better so, to re-think the structure of the manuscript and move away from the criteria.

**We strongly believe that we have now justified each of our nine indicators across the five categories (Table 1). Without a quantitative approach to evaluating reintroduction success, this leaves lots of room open for subjectivity which does not help our knowledge base of reintroduction biology. This indicator framework can now be used for other wild dog reintroduction efforts and for a range of other carnivores globally. Thus, we think it makes a valuable and critical addition to our study that uses baseline ecological data (that should be collected anyway) to evaluate reintroduction success. This will be used by GNP management to further guide decision making on future introductions.**

I hope my comments will help in focusing the manuscript on key aspect of reintroduction, thus rendering it shorter and more accessible.

**Your comments very much helped make this a better paper, and we very much appreciated your attention to such detail.**
